# Meta-Analysis of Optical Surrogates for the Characterization of Dissolved Organic Matter

**DOI:** 10.1021/acs.est.3c10627

**Published:** 2024-04-19

**Authors:** Julie A. Korak, Garrett McKay

**Affiliations:** † Department of Civil, Environmental, and Architectural Engineering, University of Colorado, Boulder, Colorado 80309-0428, United States; ‡ Environmental Engineering Program, University of Colorado, Boulder, Colorado 80303, United States; § Zachry Department of Civil & Environmental Engineering, Texas A&M University, College Station, Texas 77843, United States

**Keywords:** organic matter, optical surrogates, absorbance, fluorescence, fluorescence indices, spectral slope, absorbance ratio

## Abstract

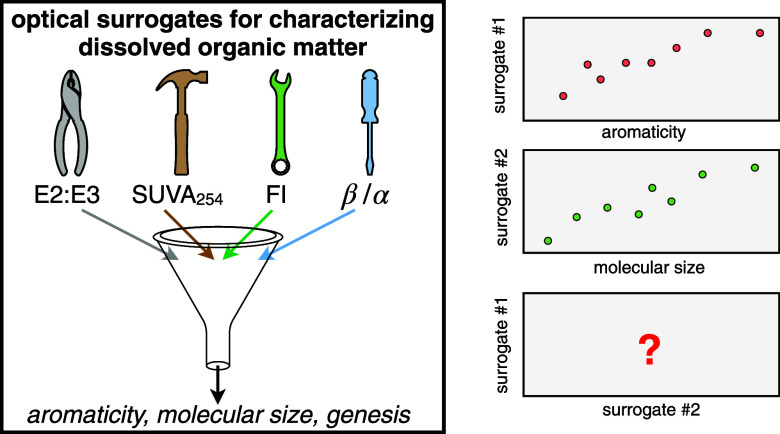

Optical surrogates, derived from absorbance and fluorescence spectra, are widely used to infer dissolved organic matter (DOM) composition (molecular weight, aromaticity) and genesis (autochthonous vs allochthonous). Despite the broad adoption of optical surrogates, several limitations exist, such as context- and sample-specific factors. These limitations create uncertainty about how compositional interpretations based on optical surrogates are generalized across contexts, specifically if there is duplicative or contradictory information in those interpretations. To explore these limitations, we performed a meta-analysis of optical surrogates for DOM from diverse sources, both from natural systems and after water treatment processes (*n* = 762). Prior to analysis, data were screened using a newly developed, standardized methodology that applies systematic quality control criteria before reporting surrogates. There was substantial overlap in surrogate values from natural and treated samples, suggesting that the gradients governing the surrogate variability can be generated in both contexts. This overlap provides justification for using optical surrogates originally developed in the context of natural systems to describe DOM changes in engineered systems, although the interpretations may change. Absorbance-based surrogates that describe the amount of spectral tailing (e.g., E2:E3 and *S*_275–295_) had a high frequency of strong correlations with one another but not to specific absorbance (SUVA_254_) or absorbance slope ratio (*S*_R_). The fluorescence index (FI) and biological index (β/α) were strongly correlated with one another and to the peak emission wavelength but not to the humification index (HIX). Although SUVA_254_ and FI have both been correlated to DOM aromaticity in prior research, there was a lack of reciprocity between these optical surrogates across this data set. Additionally, there were patterns of deviations in the wastewater subset, suggesting that effluent organic matter may not follow conventional interpretations, urging caution in the use of optical surrogates to track DOM in water reuse applications. Finally, the meta-analysis highlights that three aspects should be captured when optical spectra are used for DOM interpretation: specific absorbance, absorbance tailing, and the extent of red-shifted fluorescence. We recommend that SUVA_254_, E2:E3, and FI or β/α be prioritized in future DOM studies to capture these aspects, respectively.

## Abstract 2

A meta-analysis of optical surrogates for characterizing dissolved organic matter identifies which surrogates are likely to interrogate different pools of chromophores and fluorophores. Physicochemical properties outside the conventional interpretations of molecular weight and aromaticity also govern optical surrogates.

## 1 Introduction

Absorbance and fluorescence are two widely used techniques to characterize dissolved organic matter (DOM) in natural and engineered systems.^1−3^ DOM absorbance spectra are broad and featureless, with absorbance decaying exponentially with increasing wavelength. DOM fluorescence spectra from diverse environments consistently exhibit a characteristic set of peaks (i.e., peaks A and C), with other peaks showing greater prominence depending on the source (i.e., peaks B, T, and M).^4,5^ In addition to qualitative spectral interpretation, quantitative optical surrogates have been developed. These surrogates distill spectral shape into scalar values, with the goal of relating DOM composition (e.g., molecular weight, aromaticity) and genesis (e.g., autochthonous and allochthonous) to the optical surrogate.^6,7^ Indeed, several studies have demonstrated strong relationships between optical surrogates and DOM chemical composition, such as molecular weight or aromaticity determined by ^13^C NMR or ultrahigh resolution mass spectrometry.^6−13^ Although these studies are typically limited in context, there is growing use of optical surrogates as indirect measurements of DOM composition and genesis.^1^

Despite their convenience, few studies have considered the redundancy or mutual exclusivity of different optical surrogates. For example, three of the most studied optical surrogates are specific absorbance at 254 nm (SUVA_254_, dissolved organic carbon-normalized absorbance),^6^ E2:E3 (ratio of absorbance at 250 to 365 nm),^8,14^ and fluorescence index (FI, the ratio of emission intensities at 470 and 520 nm at a 370 nm excitation wavelength).^7,15^ Conventional interpretation is that higher SUVA_254_, lower E2:E3, and lower FI are characteristic of DOM with higher aromaticity, higher molecular weight, and from terrestrial environments.^6−8^ However, little information is available about the covariation of these optical surrogates for a large, common set of DOM samples.

The underlying basis for using optical surrogates is that the chemical structures responsible for absorbance and fluorescence signals overlap with structures that govern the DOM reactivity in natural and engineered systems. For example, the reaction of DOM with oxidants during water treatment (e.g., chlorine and ozone) decreases DOM absorbance.^16−18^ This decrease has been attributed to the oxidation of phenolic moieties, which absorb light in the ultraviolet (UV) region of the spectrum.^17^ Phenols are an example of a chromophore, which is a specific compound, or substructure of a larger molecule, that can absorb light at wavelengths of interest.^19^ DOM chromophores are composed of individual molecules containing sp^2^-hybridized carbon and, possibly, associations of small molecules that absorb and emit at lower energies (e.g., donor–acceptor complexes).^20−22^ Upon light absorption, chromophores quickly relax to the lowest vibrational level of the first excited singlet state, after which a fraction of singlet excited states radiatively relax to the ground electronic state (i.e., fluorescence). The wavelength and intensity of fluorescence are quantitatively related to the underlying relaxation pathways by the Stokes shift and fluorescence quantum yield.^23,24^

Although optical surrogates have been broadly adopted by different research communities to describe DOM physicochemical properties, several limitations are frequently not considered. First, optical surrogates were proposed in specific contexts, whether a certain geographic environment or a sample preparation method. For example, the original studies demonstrating correlations between E2:E3 and molecular size focused on a single lake water sample that had either been pH adjusted or ultrafiltered into different size fractions.^8,14^ In contrast, Weishaar et al. (2003) demonstrated a positive linear relationship between SUVA_254_ and aromaticity by ^13^C NMR for a geographically diverse set of aquatic samples, but the context was constrained to isolate materials, which was necessary for solid-state NMR analysis.^6^ These geographic and sample-specific factors are often not considered in studies that generalize interpretations to whole-water DOM. Second, studies of DOM fate in engineered systems frequently apply generalizations developed from inquiries into natural systems to infer changes in DOM physicochemical properties. However, it is not well understood whether the compositional gradients governing optical surrogates in natural systems overlap with the compositional gradients governing optical surrogates resulting from engineered treatments. Third, interpreting optical surrogates as mutually exclusive definitive indicators of DOM molecular weight, aromaticity, or genesis could lead to two adverse outcomes. Two surrogates may be duplicative of one another if they respond to the same underlying chemical characteristics, perpetuating disparate interpretation methods. Alternatively, two surrogates may lead to conflicting interpretations if they are assumed to respond to the similar underlying characteristics (e.g., biological index and fluorescence index)^7,25^ without identifying contextual limitations. Collectively, the methodology to select optical surrogates lacks cohesiveness and creates uncertainty about which optical measurements should be prioritized to describe the composition or genesis. Addressing this uncertainty is increasingly important as more *in situ* optical sensors are developed for water quality monitoring.^26−28^

To address these knowledge gaps, we performed a meta-analysis of optical surrogates used to characterize DOM. The data set (762 samples) was curated from projects conducted primarily at the University of Colorado Boulder between 2011 and 2021 that spanned a range of studies, from fundamental inquires using DOM isolates to applied treatment studies. A range of sample origins was studied, including whole-waters from across the contiguous United States, isolate materials, and secondary-treated wastewater effluent. A study by Hansen et al. (2016) evaluated correlations between optical surrogates for DOM leached from different source materials and subjected to microbial and photochemical transformations.^29^ In comparison, our study evaluates a broader context of the DOM sources and treatment processes. Since interpretations of surrogates were predominantly developed in the context of natural systems, including samples from both natural and treated contexts is imperative to evaluate if the compositional gradients governing surrogates transcend the applications in which surrogates are currently used. This meta-analysis addresses several of the key limitations about redundancy and highlights contradictions between optical surrogates, which we expect will advance how surrogates are applied to characterize DOM in natural and engineered systems.

## 2 Materials and Methods

### 2.1 Data Collection and Processing

The meta-analysis data set was curated from predominantly published articles and publicly available theses.^18,22,30−43^ Each sample was categorized using four attributes, which differentiated the data set into meaningful subsets (Table 1 and SI Text S2). Sample context categorized samples as either natural or treated, the latter referring to samples subjected to treatment processes, for example, coagulation and chemical oxidation. The Treated subset was further categorized by sample treatment. Independently, DOM fractionation differentiated Whole-Water and Isolate subsets, where the Isolate subset includes samples isolated via XAD resin^44,45^ or reverse osmosis.^46,47^ Lastly, DOM origin classifies the environmental system from which the sample was collected. One limitation in the data set is a lower representation of estuarine and marine samples compared to freshwater aquatic sources. No soil samples were included due to under-representation and low diversity in the available source materials. Table S4 shows the broad distribution of samples across these categories, indicating that conclusions derived from this study could be applicable to DOM in multiple aquatic contexts.

**Table 1:** Sample Classifications and the Total Number of Samples in each Class (Calculated for All Data)

sample context	DOM fractionation	DOM origin	sample treatment^a^
natural (203)	whole-water (572)	aquatic^b^ (626)	coagulation^c^ (108)
treated (559)	isolate (190)	wastewater (107)	ozonation^d^ (67)
		estuary (15)	granular activated carbon^e^ (178)
		stormwater (14)	coagulation and PAC^f^^g^ (40)
			pre-oxidation then coagulation^h^ (110)
			biological filtration^i^ (29)
			borohydride reduction^i^ (7)
			oxidation only (Cl_2_ or ClO_2_)^i^ (2)

a Size-fractionated samples (*n* = 81) were listed under a separate category from sample treatment.

b Constrained to freshwater samples

c Coag in figure legends

d Ozone in figure legends

e GAC in figure legends

f Coag + PAC in figure legends

g Powdered activated carbon

h Preox + Coag in figure legends

i Not included in Figures 2–4 due to small sample size

Sample processing followed best practices, including collecting and storing samples in muffled glassware, filtering with muffled glass fiber filters (0.7 μm), and refrigerating the samples until analysis. Two of the studies used 0.45 μm poly(ether sulfone) syringe filters instead of muffled glass fiber filters.^34,48^ Over 96% of samples were analyzed on the same spectrofluorometer (Fluoromax-4, Horiba), spectrophotometer (Cary-100 Bio, Agilent), and one of two organic carbon analyzers (M5310C, GE or TOC-V_CSH_, Shimadzu). A small percentage of the data set (4%) was collected with an instrument that measures fluorescence and absorbance simultaneously (Aqualog, Horiba).

For consistency, the meta-analysis started with the original absorbance spectrum (as measured), corrected fluorescence excitation–emission matrix (EEM), and dissolved organic carbon (DOC) concentration for each sample. EEMs were corrected following the method in Murphy et al. (2010),^49^ including blank subtraction, Raman normalization at excitation 350 nm, instrument-specific corrections, and corrections for primary and secondary inner filter effects. EEMs were also masked and interpolated for first- and second-order Rayleigh and Raman scattering, respectively. Standard operating procedures included regular quality control checks such as lamp scans, monochromator alignment verification, blank and duplicate analysis, and DOC check standards.

### 2.2 Calculation of Optical Surrogates

This study focuses on intrinsic (concentration-independent) surrogates (Table 2). Examples of intrinsic absorbance surrogates include specific (carbon-normalized) absorbance (e.g., SUVA_254_), absorbance spectral slopes (e.g., *S*_275–295_, *S*_300–600_), and ratios of absorbance (e.g., E2:E3). Intrinsic fluorescence surrogates include specific peak intensities (e.g., SpC), various indices (e.g., HIX, FI, β/α), and wavelengths of maximum fluorescence intensity (e.g., λ_max_(370 nm)). Figure S1 depicts the wavelength regions of select surrogates.

Optical surrogates were calculated using a consistent methodology; the methodology is described in Supporting Information Text S1 and MATLAB codes are published.^50^ Briefly, a quality control method was applied to each optical surrogate to avoid bias driven by low signal-to-noise ratios or outliers. A detection level for absorbance was determined by calculating the absorbance standard deviation for each sample above 600 nm; a threshold was set at 20 times the 95th percentile of the observed noise. This calculation resulted in an absorbance threshold of 0.005 for our study, and absorbance surrogates were not reported if the as-measured absorbance at a relevant wavelength was below this threshold. The percentage of values excluded based on this analysis was 1.8% of SUVA_254_, 24.8% of E2:E3, 3.4% of *S*_275–295_, 42.1% of *S*_350–400_, and 4.2% of *S*_300–600_.

A different approach was developed for fluorescence surrogates, identifying spectra with high noise, rather than low intensity, because the corrected fluorescence intensity is a function of the measured fluorescence intensity, lamp intensity, instrument-specific corrections, and inner filter corrections. The relative noise in a fluorescence spectrum was calculated by comparing the corrected intensities to the central tendency of a smoothed spectrum. Based on the cumulative distribution of relative noise (Figure S5), a maximum noise threshold was set at the 95th percentile unique to each excitation wavelength. The percentage of values excluded based on this analysis was 5.8% of FI, 5.5% of β/α, and 5.4% of HIX.

**Table 2:** Summary of Intrinsic Optical Surrogates^a^ Included in the Meta-Analysis

technique	surrogate	examples	references
absorbance	specific ultraviolet absorbance	SUVA_254_, SUVA_280_, SUVA_320_, SUVA_370_	(6, 11), (51)
	absorbance ratio	E2:E3, E4:E6	(8, 14), (52)
	spectral slope (nonlinear)	*S*_300–600_, *S*_300–650_, *S*_300–700_	(9, 53), (54)
	spectral slope (linear)	*S*_275–295_, *S*_350–400_	
	spectral slope ratio	*S*_R_	
fluorescence	humification index	HIX	(55, 56)
	biological index/freshness index	β/α, BIX	(13), (25), (57)
	fluorescence index	FI	(7, 15)
	maximum emission wavelength	λ_max_(254 nm)	
		λ_max_(310 nm)	
		λ_max_(370 nm)	
	specific peak intensities	SpA, SpB, SpC, SpT	(4, 38)

a Definitions and calculation methods are included in SI Text S1.

To avoid large propagated error from low DOC concentrations,^38^ carbon-normalized surrogates were not reported for samples with DOC < 0.5 mg_C_ L^–1^. To avoid statistical leverage by extreme outliers, the upper and lower 0.5 percentiles for absorbance and fluorescence surrogates were excluded from the meta-analysis (Figure S7). The specific thresholds for each optical surrogate reported herein are specific to the instruments, methods, and sample context and therefore should not be used indiscriminately. Future studies could apply the same methodology to determine study- and instrument-specific quality control thresholds.

### 2.3 Statistical Analyses

Samples passing the quality control criteria were divided into subsets using the categories in Table 1. Aggregate statistics were calculated for each subset, including the minimum, 25% quartile, mean, median, 75% quartile, maximum, and number of samples passing the quality control thresholds (*n*). These results are compiled in Tables S6–S17.

Relationships between optical surrogates were evaluated by calculating Spearman’s rho (*ρ*_*S*_) and the associated *p*-value (*p*_S_). ρ*_S_* was chosen over Pearson’s rho and *R*^2^, because ρ*_S_* identifies monotonic correlations that are not necessarily linear. Correlations between two optical surrogates were classified as strong if |*ρ*_*S*_| > 0.75 and statistically significant if *p*_S_ < 0.01. Violin plots were made using Bechtold (2016).^58^

## 3 Results

### 3.1 Overview of the Meta-Analysis Data Set

#### 3.1.1 Variability of Optical Parameters in the Meta-Analysis Data Set Compared to Literature Data

**Figure 1:**
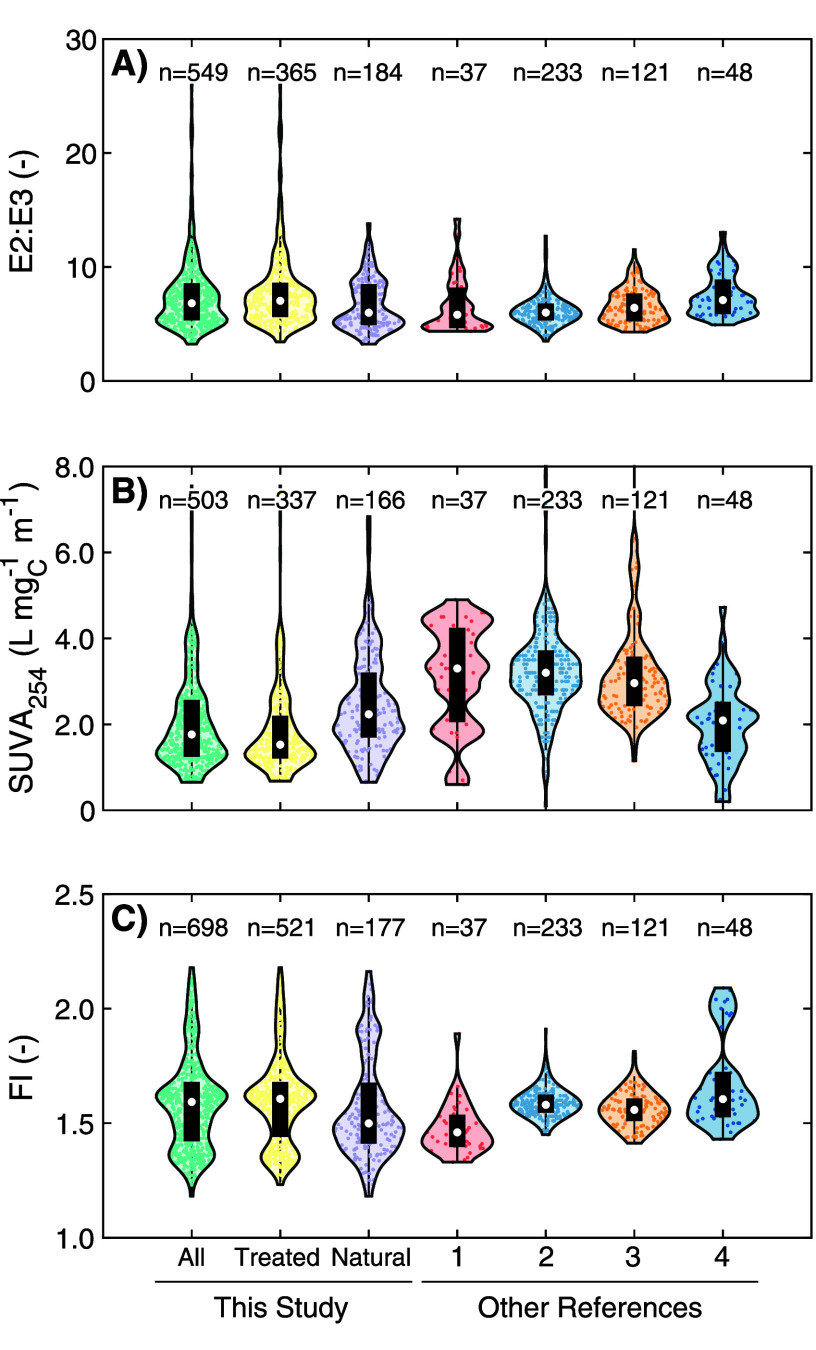
Distribution of (A) E2:E3, (B) SUVA_254_, and (C) FI values between subsets within the meta-analysis (all, treated, and natural) and other literature references (1: Kellerman et al. (2018),^59^ 2: McCabe and Arnold (2017),^60^ 3: McCabe and Arnold (2018),^61^ 4: Berg et al. (2023).^62^

The meta-analysis data set (*n* = 762) captures a breadth of optical characteristics representative of DOM from diverse natural sources (*n* = 203) and treatment scenarios (*n* = 559). Samples are well-represented between both categories, which is important for drawing conclusions for engineered systems based on surrogates developed for natural systems. Figure 1 shows that the range of values is large for three frequently used optical surrogates (i.e., SUVA_254_, E2:E3, and FI). The aggregate statistics and violin plots for all optical surrogates, documented in SI Text S2, demonstrate the breadth and depth of this data set.

The distribution of E2:E3, SUVA_254_, and FI values broadly overlapped between the Natural and Treated subsets (Figure 1). Comparing the Treated to Natural subsets, median values for E2:E3 (7.03 vs 5.99) and FI (1.61 vs 1.50) were greater, and the median SUVA_254_ (1.5 vs 2.2 L mg_C_^–1^ m^–1^) was lower in the Treated subset. Also in the Treated subset, E2:E3 ratios greater than 12 (top 5% and uncommon in natural samples) are from the Ozonation subset. Higher SUVA_254_ commonly co-occurred in samples with lower E2:E3, lower β/α, and higher HIX, consistent with prior literature.^59−61,63^ Using conventional interpretations, these differences would imply that the DOM in the Treated subset was, on average, less aromatic and of lower molecular weight than the Natural subset. A systematic shift in composition for the Treated subset aligns with sample context of common water treatment processes, which are designed to preferentially remove (e.g., coagulation, granular activated carbon (GAC))^37,41,64^ or oxidize (e.g., ozonation)^17,18,65,66^ aromatic moieties associated with SUVA_254_. SUVA_254_ values greater than 5.5 L mg_C_^–1^ m^–1^ are uncommon in aquatic systems and are usually indicative of interfering constituents.^6,67,68^ Samples in the meta-analysis data set with SUVA_254_ > 5.5 L mg_C_^–1^ m^–1^ were <3% of the data set passing the QC criteria (*n* = 6) and comprised of a >10 kDa fraction of a wastewater effluent, one sample from the Florida Everglades and a Suwannee River humic acid isolate prepared at different conditions. Collectively, these results demonstrate that there is broad overlap in the DOM compositional gradients governing optical surrogates in natural and engineered systems.

The meta-analysis data set was also compared to peer-reviewed data sets that included a similar breadth of optical surrogates with contextually diverse samples. Only studies that tabulated optical surrogates were considered (i.e., values were not extracted from figures). Figure 1 compares the meta-analysis data set to four reference data sets. Kellerman et al. (2018), Reference 1, characterized DOM isolates (hydrophobic organic acids (HPOA), fulvic acids, and natural organic matter) for diverse aquatic ecosystems around the world.^59^ McCabe and Arnold (2017), Reference 2, curated a data set of stormwater samples across a gradient of land use (vegetated to developed), incorporating urban-impacted DOM.^60^ McCabe and Arnold (2018), Reference 3, includes samples from wetlands in Minnesota under a range of hydrologic conditions.^61^ Finally, Berg et al. (2023), Reference 4, includes a wide range of freshwater systems and wastewater samples.^62^

The range of values in the meta-analysis data set is as wide or wider than the reference data sets for E2:E3, SUVA_254_, and FI (Figure 1) and seven other optical surrogates compared to seven additional data sets (SI Text S3).^9,63,69−73^ Exceptions are attributable to sample origins that are not well-represented in the meta-analysis (e.g., marine DOM). The large range of values indicates that trends observed in the meta-analysis may be applicable to DOM across large compositional gradients.

#### 3.1.2 Optical Surrogate Interferences

It is well-known that DOM optical surrogates can be impacted by co-occurring inorganic constituents such as nitrate, nitrite, and iron (Fe) species. These species absorb light at UV wavelengths, which could confound absorbance-based surrogates if the concentration of interfering species is high enough.^67,74^ In addition, metals such as iron quench DOM fluorescence and impact fluorescence-based surrogates.^67^

The analysis presented herein does not correct optical surrogates for potential interference by nitrate, nitrite, or iron. Most of the meta-analysis studies did not measure these species, either because meaningful concentrations were not expected given the source water context or because their presence was not aligned with the study objective. The choice not to correct optical surrogates based on potential interferences is justified for the following reasons. First, such corrections are not standard practice in the literature, including three of the four references described above, and a goal of the meta-analysis was to seek correlations under a broad range of cases, aligning with the way interpretations have been generalized across applications. Second, in the case of iron, there is no universal correction factor that is independent of the DOM composition, iron redox states, or other sample-specific factors. Finally, prior research and our analysis (see SI Text S2) identified that the Wastewater subset would be most susceptible to iron or nitrate interferences. However, at the nitrate and iron concentrations typical for wastewater, the expected impact to SUVA_254_ and E2:E3 is <10%. Although not possible to rule out fluorescence quenching, the expected Fe:DOC concentrations in wastewater (0.01–0.02 mg_Fe_ mg_C_^–1^) are not expected to increase FI by more than 3%.^67^

Although the context of the meta-analysis justified not correcting absorbance or fluorescence surrogates for interfering species, we caution against making this practice a generalization. For example, waters impacted by agricultural runoff may contain much higher nitrate concentrations, e.g., 30 mg-N L^–1^.^75^ Assuming a true SUVA_254_ (due to DOM) of 2.0 L mg_C_^–1^ m^–1^ and a DOC concentration of 7.0 mg_C_ L^–1^, not accounting for nitrate absorbance would lead to a 25% error (as-measured SUVA_254_ of 2.5 L mg_C_^–1^ m^–1^). Under the same conditions, E2:E3 would be similarly impacted, reporting 9.2 versus 7.5 without nitrate corrections. Applying the same approach to evaluate the interference of 0.1 mg L^–1^ total iron leads to ∼5% higher values for SUVA_254_ and E2:E3.

### 3.2 Correlation of Optical Parameters

To explore relationships between optical surrogates, correlations were evaluated across sample subsets for absorbance- and fluorescence-based surrogates. If two optical surrogates probe similar structural information, then the transitive property would suggest that the correlation between those optical surrogates should be both strong (|ρ*_s_*| > 0.75) and statistically significant (*p*_S_ < 0.01). For example, studies have independently documented that SUVA_254_ and FI are proportionally and inversely correlated to aromaticity, respectively.^6,7^ However, few studies have critically evaluated whether SUVA_254_ is strongly correlated to FI. Here, we test this transitive logic across the meta-analysis subsets using Spearman rho values, which do not assume linear relationships or normalized data.

#### 3.2.1 Correlation between Absorbance-Based Surrogates

**Figure 2:**
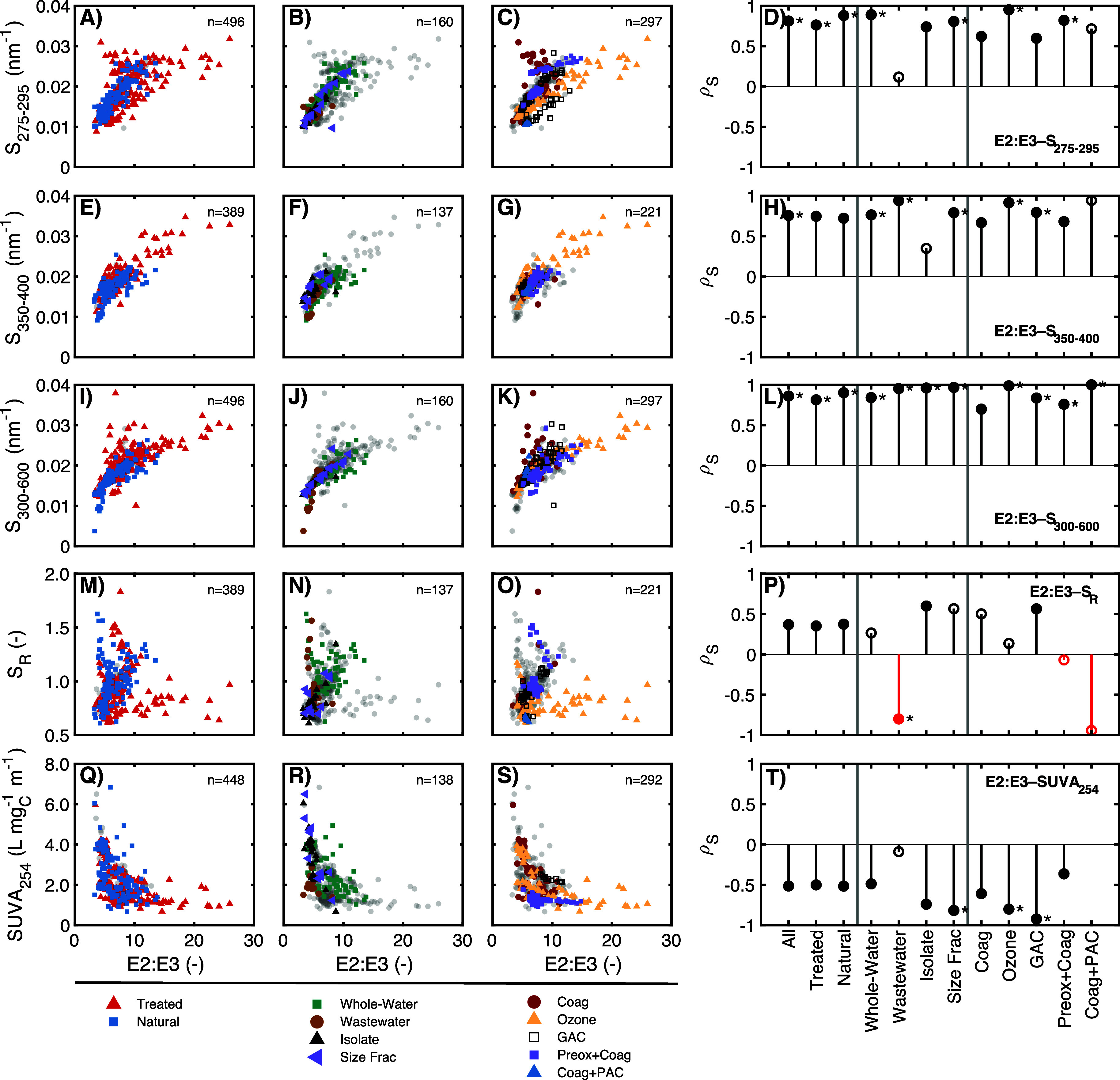
Relationships between E2:E3 and (A)–(D) the spectral slope between 275 and 295 nm (S_275–295_), (E)–(H) spectral slope between 350 and 400 nm (*S*_350–400_), (I)–(L) spectral slope between 300 and 600 nm (*S*_300–600_), (M)–(P) the spectral slope ratio (*S*_R_), (Q)–(T) specific ultraviolet absorbance at 254 nm (SUVA_254_). The number of highlighted samples in each scatterplot is indicated by *n*. Lollipop plots in (D), (H), (L), (P), and (T) show Spearman rho value (*ρ*_*s*_) for optical surrogate correlations for specific data subsets. Closed symbols represent a statistically significant relationship (*p*_S_ < 0.01), while open symbols represent an insignificant relationship. Markers with an asterisk (*) indicate |*ρ*_*s*_|>0.75 and are shown only if *p*_S_ < 0.01. Correlations are missing for Coag + PAC due to no DOC measurements for these samples.

Absorbance-based surrogates that describe the amount of spectral tailing (e.g., E2:E3, spectral slope) had a high frequency of strong correlations with one another (Figure 2A–L). In contrast, E2:E3 and spectral slopes showed a low frequency of strong correlations to SUVA_254_ and *S*_R_ (Figures 2M–T, S17–S20 and S25).

Relationships between E2:E3 and spectral slopes were both strong and significant for 24 of the 36 subset correlations shown in Figure 2. Of the three spectral slope wavelength ranges evaluated, *S*_300–600_ had more subsets with strong and significant correlations to E2:E3 (11 of 12, Figure 2L) than did *S*_275–295_ (7 of 12, Figure 2D) or *S*_350–400_ (6 of 12, Figure 2H). The greater frequency of strong correlations between E2:E3 and both *S*_300–600_ and *S*_275–295_ compared to *S*_350–400_ may be due to greater sensitivity of *S*_275–295_ to gradients in natural and engineered environments. For example, the interquartile range for *S*_275–295_ across the entire data set was 3-fold wider than that for *S*_350–400_ (Table S7). Interestingly, although the Wastewater subset had relatively uniform *S*_275–295_ values and the correlation between E2:E3 and *S*_275–295_ was neither significant nor strong, correlations between E2:E3 and both *S*_350–400_ and *S*_300–600_ for the Wastewater subset were strong and statistically significant. This observation points to a unique characteristic of wastewater DOM composition, where the lack of correlation in the 275–295 nm range could be due to either more uniform DOM composition or interference from non-DOM chromophores (Section 3.1.2, SI Text S2 and Figure S9). For example, molecular weight fractionation of aquatic DOM has consistently shown that SUVA_254_ decreases in lower molecular weight fractions;^11,30,73,76^ however, SUVA_254_ does not appear to consistently change with molecular weight for wastewater DOM.^30,77^ Across the Natural and Treated subsets as a whole, the high frequency of strong correlations between E2:E3 and spectral slope suggests that these optical surrogates are probing similar aspects of DOM composition, especially when evaluated across a larger wavelength range (i.e., *S*_300–600_).

In contrast to E2:E3 and spectral slopes, correlations between *S*_275–295_, *S*_350–400_, *S*_300–600_, and *S*_R_ had a low frequency of being classified as strong (8 of 36), although many (26 of 36) were statistically significant (Figure S25). The high frequency of statistically significant relationships between spectral slope parameters contrasts with the conclusion of Hansen et al. (2016)^29^ that *S*_275–295_, *S*_350–400_, and *S*_R_ are tracking different DOM pools. The conclusion in Hansen et al. (2016) was based on correlations between spectral slope parameters having low coefficients of determination (*R*^2^ < 0.65), presumably from linear regressions. Linear regressions between *S*_275–295_, *S*_350–400_, and *S*_R_ from the meta-analysis data set also showed low *R*^2^ values (*R*^2^ < 0.65) (Figures S21–S24). In contrast, the same correlations had statistically significant Spearman correlations (most ρ_*s*_ > 0.5), suggesting an underlying relationship albeit nonlinear. Overall, this comparison demonstrates the advantage of Spearman’s ρ*_s_* to identify correlated surrogates.

Correlations between E2:E3 and SUVA_254_ had a low frequency of being classified as strong (3 of 11) although many (10 of 11) were statistically significant (Figure 2Q–T). For example, the entire range of SUVA_254_ values (1 to 6 L mg_C_^–1^ m^–1^) were observed at the median E2:E3 value of 6.8 (Figure 2Q). Similarly, a large range of E2:E3 values (3 to 20) was observed at SUVA_254_ values less than 2 L mg_C_^–1^ m^–1^. These results suggest that E2:E3 and SUVA_254_ do not yield redundant information across a broad range of sample environments and water treatment processes. There were, however, narrower sample contexts in which correlations may indicate redundant information. For example, the Isolate, GAC, and Ozonation subsets have |*ρ*_*s*_| values >0.74 (Figure 2T), which may be due to a larger fraction of each subset being associated with the same source water (e.g., multiple treatment levels of a single source). The strong correlation between E2:E3 and SUVA_254_ observed for the Isolate subset is consistent with results from Kellerman et al. (2018),^59^ which characterized a large set of mostly HPOA isolates (Figure S38A, ρ*_s_* = 0.837, *p*_S_ < 0.01). In contrast, weaker correlations between E2:E3 and SUVA_254_ were observed in filtered whole-waters from McCabe and Arnold^60,61^ and Berg et al.^62^ (Figure S38B–D). The lack of a transitive relationship between E2:E3 and SUVA_254_ across DOM from diverse sources and engineering treatments indicates that the often-assumed covariation in DOM molecular weight and aromaticity inferred from optical surrogates^11,33,35,36,78^ should be treated with caution.

Similar to SUVA_254_, most correlations between E2:E3 and *S*_R_ were classified as weak and many were not statistically significant (Figure 2M–P). This result is likely because the gradients in DOM composition governing E2:E3 cause *S*_275–295_ and *S*_350–400_ to change in a similar proportion, leading to a minimal net effect on *S*_R_. The Ozonation subset illustrates this trend (Figure 2O). E2:E3 values span the range of the data set (4.1 to 26), while the range of *S*_R_ values is much smaller (0.612 to 1.17) (Table S16). The lack of change in *S*_R_ for the Ozonation subset is consistent with the proportional changes in *S*_275–295_ and *S*_350–400_ for the same samples across ozone doses (Figures 2C,G, and S25C and S25K).^79^ These comparisons suggest that E2:E3, *S*_275–295_, and *S*_350–400_ are more sensitive than *S*_R_ to shifts in DOM composition. This interpretation is consistent with results from the paper detailing the genesis of this surrogate, Helms et al. (2008),^9^ where size-fractionated Suwannee River natural organic matter exhibited only an ∼4% shift in *S*_R_ between the high (3000 Da) and low (1000 Da) molecular weight fractions, whereas *S*_275–295_ and *S*_350–400_ exhibited a >40% shift.

Figure 2 also demonstrates that the trajectory of some correlations is sample- and treatment-specific. For example, although the GAC subset does not have a strong correlation between E2:E3 and *S*_275–295_ (Figure 2C,D), a closer examination of Figure 2C shows two subgroups of GAC-treated samples with internally consistent correlations. Similar slopes between subsets indicate that E2:E3 and *S*_275–295_ both increase in similar proportion, but the y-intercepts differ between source waters (i.e., freshwater aquatic vs wastewater). A similar behavior is observed for the Preox + Coag subset (Figure 2C,D), where two sources (reservoir vs canal) were oxidized with ozone, chlorine dioxide, or potassium permanganate and then coagulated with alum, ferric chloride, or aluminum chlorohydrate.^39^ The canal source was collected as a time series during flushing and only preoxidized with permanganate.^39^ For each source, there were internally consistent surrogate correlations, aligning with conventional interpretations (i.e., oxidation and coagulation selectively remove or destroy high molecular weight and more aromatic DOM molecules), but these correlations did not transcend differences in starting material composition. E2:E3 and SUVA_254_ also show sample-specific trajectories, which, although not strong for most of the data set (GAC and Ozonation subsets are exceptions, Figure 2T), are negatively correlated with most ρ*_S_* values less than −0.5.

Although it is often assumed that SUVA_254_ and spectral shape surrogates (i.e., E2:E3 and spectral slopes) yield redundant information about DOM composition, the breakdown in correlations between surrogates across the meta-analysis indicates otherwise. For treatment processes, the trajectories of optical surrogates behave in a manner internally consistent with the expected causal relationship but not across different starting compositions. This breakdown is further supported by the strong and significant relationship between E2:E3, spectral slopes, and SUVA_254_ for the Size-Fractionation subset (Figure 2D, H, L and T). A practical implication is that, across a wide range of sample types and treatments, similar information can be gained from E2:E3 and the spectral slope. In contrast, the lack of transitive relationship between E2:E3 and SUVA_254_ indicates that these optical surrogates are not duplicative.

#### 3.2.2 Correlation of Fluorescence-Based Surrogates

**Figure 3:**
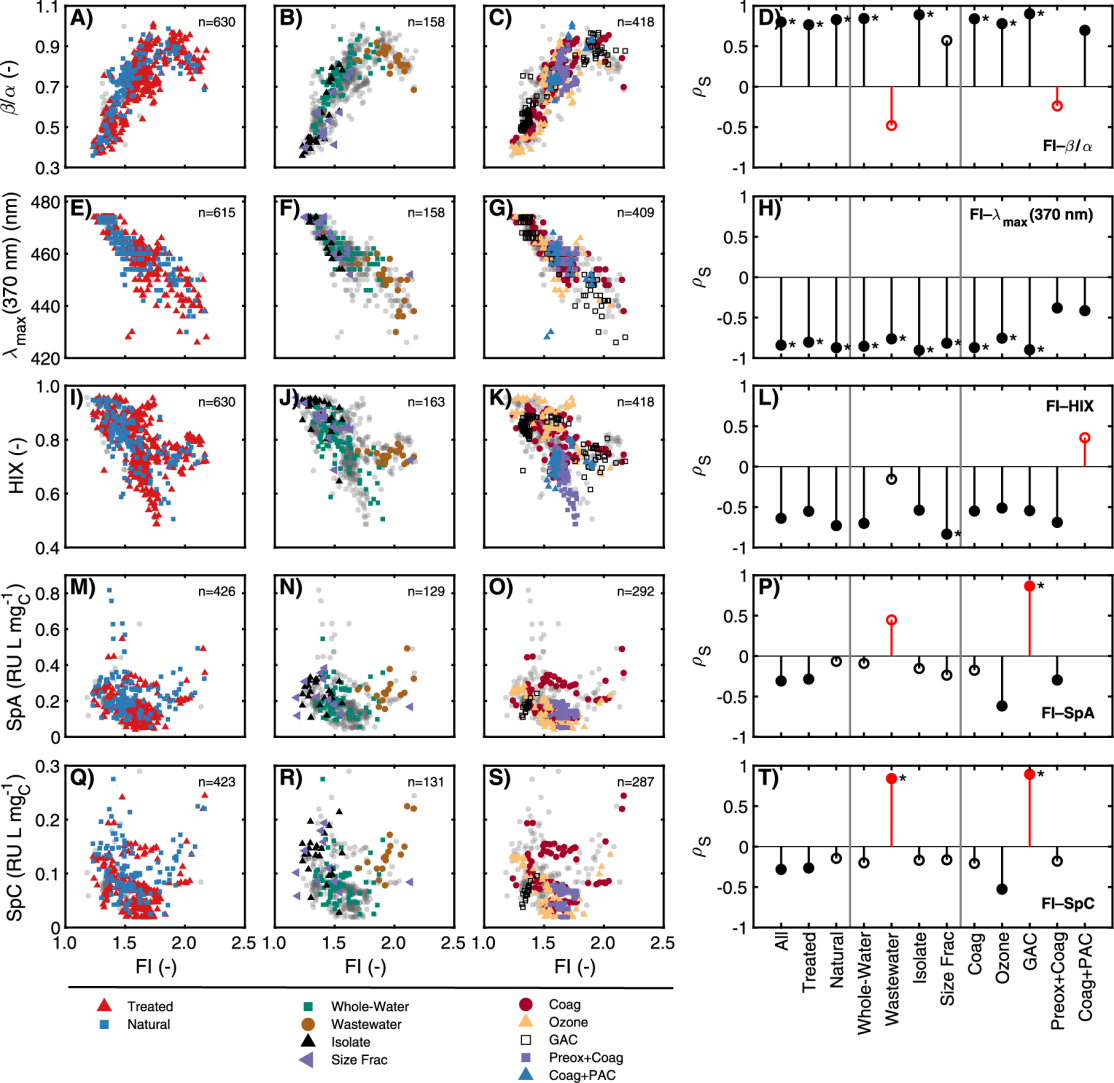
Relationships between the fluorescence index (FI) and the (A)–(D) biological index (β/α), (E)–(H) peak emission at 370 nm excitation (λ_max_(370 nm), nm), (I)–(L) humification index (HIX), (M)–(P) specific peak A (SpA, RU L mg_C_^–1^), and (Q)–(T) specific peak C (SpC, RU L mg_C_^–1^). The number of highlighted samples in each scatterplot is indicated by *n*. Lollipop plots in (D), (H), (L), (P), and (T) show Spearman rho value (*ρ*_*S*_) for optical surrogate correlations for specific data subsets. Closed symbols represent a statistically significant relationship (*p*_S_ < 0.01) while open symbols represent an insignificant relationship. Markers with an asterisk (*) indicate |*ρ*_*S*_|>0.75 and are shown only if *p*_S_ < 0.01. Correlations are missing for Coag+PAC due to no DOC measurements for these samples.

Fluorescence-based surrogates derived from ratios of emission intensities (i.e., β/α and FI) and the maximum emission wavelength (λ_max_) had a high frequency of strong correlations (Figures 3 and S17–S20). In contrast, there was a low frequency of strong correlations between fluorescence indices and specific peak intensities.

Correlations between FI and β/α were strong and significant in 8 of 12 subsets (Figure 3A–D), including both Natural and Treated subsets. The FI–β/α correlations that were not statistically significant were the Wastewater, Size-Fractionation, and Preox + Coag subsets, two of which had negative ρ*_s_* values, opposite in sign to the other subsets. The strong relationships observed between FI and β/α across multiple contexts provide strong evidence that these optical surrogates describe similar compositional aspects. This finding is consistent with prior conclusions derived from narrower contexts, such as studies focused on DOM isolates^59^ and from different geographic contexts (Figure S39).^60,61,70,80^

Correlations between FI and λ_max_(370 nm) were strong and significant for 10 of 12 subsets (Figure 3E–H). The inverse relationship between FI and λ_max_(370 nm) has been reported in prior studies,^7,38^ but none with as many samples tested under varying conditions. An inverse correlation was also observed between β/α and λ_max_(310 nm) (Figure S28). In contrast, no correlations between HIX and λ_max_(254 nm) were strong, although 8 of 12 were statistically significant (Figure S29), suggesting a fundamental difference in the relationship between the peak emission wavelength and HIX compared to FI or β/α. These results demonstrate that two of the most widely used fluorescence ratios, FI and β/α, are strongly related to the energy of the emitting species.

Only three of the correlations between FI and specific peak intensities (e.g., SpA and SpC) were strong, and less than half were statistically significant (Figure 3M–T). Specific peak intensities were poorly related to λ_max_(370 nm), λ_max_(310 nm), and λ_max_(254 nm) (Figures S27–S29). These results suggest that fluorescence indices and specific peak intensities describe different aspects of the DOM composition.

Only one of the correlations between FI and HIX was strong (Size-Fractionation subset, Figure 3I–L), but most were statistically significant and had ρ*_S_* values less than −0.5. Apart from the Size-Fractionation subset, the strongest relationships between FI and HIX were observed for the Natural (ρ*_S_* = −0.73) and Whole-Water (ρ*_S_* = −0.70) subsets. Like the Ozone and GAC subsets, the strong correlation for the Size-Fractionation subset may be identifying internally consistent correlations for samples with the same source material, modified at different levels, that do not hold across unfractionated DOM. The lack of strong correlations between HIX and either FI or β/α across the meta-analysis data set is surprising because HIX was originally developed to communicate the extent of red-shifted fluorescence,^55,56^ yet FI and β/α were the only surrogates correlated to the respective peak emission wavelengths. Although a previous study using DOM isolates independently showed a correlation between FI and aromaticity,^7^ it is noteworthy that the Isolate subset does not have a significant correlation between FI and HIX, but the Size-Fractionation subset does (Figure 3L). The lack of transitive relationship between HIX and either FI or β/α suggests that these surrogates are probing different compositional aspects, despite parallel factor analysis models commonly identifying dual-excitation components that overlap the regions for both HIX and FI.^5,81^

Several exceptions exist to the general trends described for fluorescence-based surrogates. Although the Natural and Treated subsets had strong positive correlations between FI and β/α, the Wastewater subset did not (Figure 3A–D). The Wastewater subset also deviated from the correlation between FI and HIX as observed for the Natural/Whole-Water and Isolate subsets (Figure 3J). Despite these exceptions, the correlation between FI and λ_max_(370 nm) held across the entire data set, including wastewater (Figures 3E–H). This discrepancy suggests that the underlying compositional characteristics governing correlations across excitation wavelengths (e.g., FI at 370 nm vs β/α at 310 nm) do not hold for wastewater-derived DOM.

#### 3.2.3 Correlations between Absorbance- and Fluorescence-Based Surrogates

**Figure 4:**
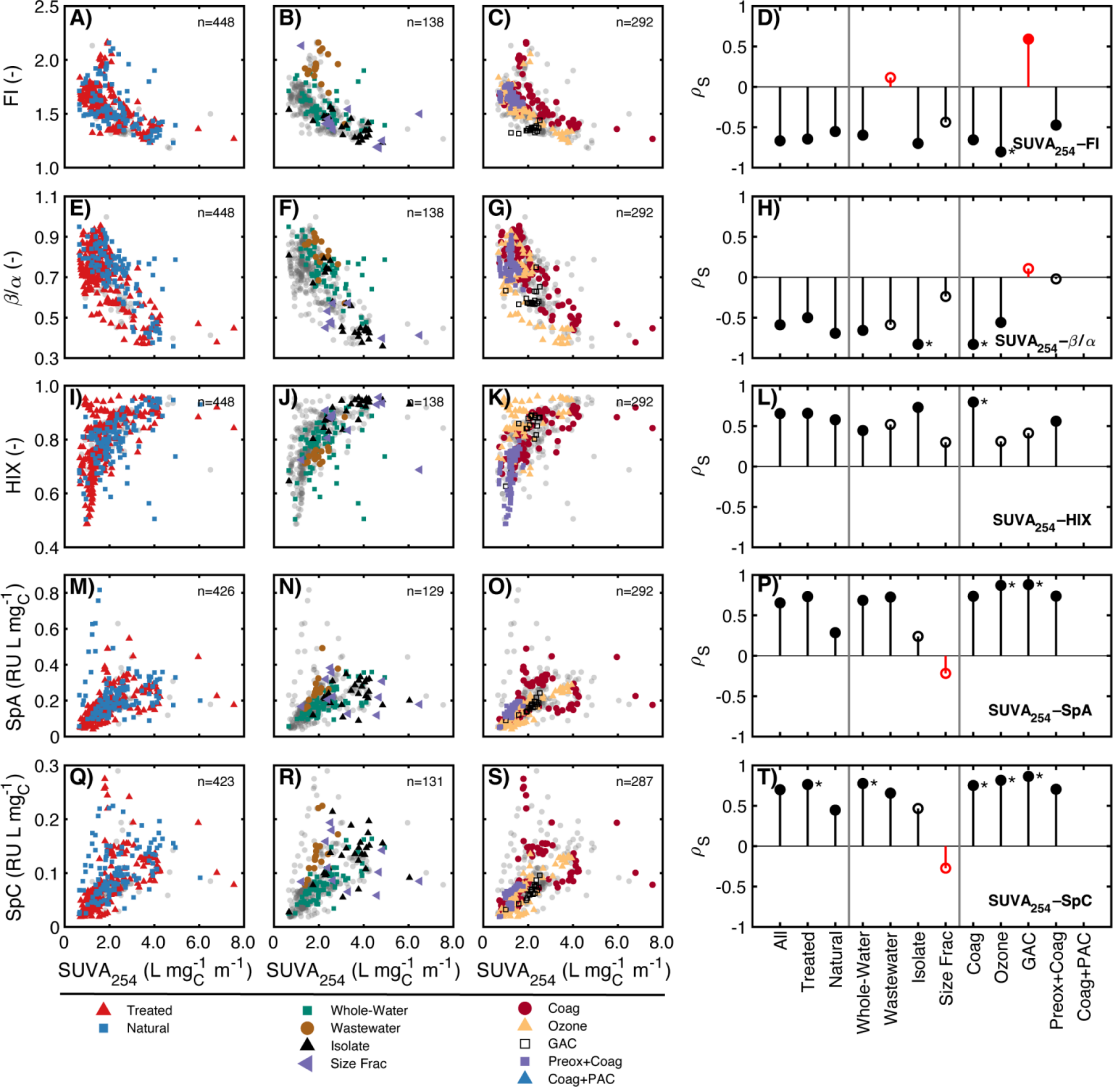
Relationships between specific ultraviolet absorbance at 254 nm (SUVA_254_) and (A)–(D) the fluorescence index (FI), (E)–(H) biological index (β/α), (I)–(L) humification index (HIX), (M)–(P) specific peak A (SpA, RU L mg_C_^–1^), and (Q)–(T) specific peak C (SpC, RU L mg_C_^–1^). The number of highlighted samples in each scatterplot is indicated by *n*. Lollipop plots in (D), (H), (L), (P), and (T) show Spearman rho value (*ρ*_*S*_) for optical surrogate correlations for specific data subsets. Closed symbols represent a statistically significant relationship (p_S_ < 0.01) while open symbols represent an insignificant relationship. Markers with an asterisk (*) indicate |*ρ*_*S*_| > 0.75 and are shown only if p_S_ < 0.01. Correlations are missing for Coag+PAC due to no DOC measurements for these samples.

Comparing absorbance- and fluorescence-based surrogates, correlations between carbon-normalized surrogates generally had larger |ρ*_s_*| values than relationships between those describing spectral shape (Figures 4 and S17–S20). In addition, fewer correlations were classified as strong compared to those within absorbance-based (Section 3.2.1) or fluorescence-based surrogates (Section 3.2.2).

Over half of the correlations between SUVA_254_ and fluorescence-based surrogates were statistically significant, but only a small fraction (11 of 55) were classified as strong. Strong and significant correlations to SUVA_254_ were observed at a higher frequency (7 of 22) for specific peak intensities (SpA and SpC, Figure 4P,T) than for fluorescence ratios (FI, HIX, β/α; 4 of 33). The greater frequency of strong correlations between SUVA_254_ and specific peak intensities may be attributed to the similarity in the underlying equations.^22^ SUVA_254_ is carbon-normalized absorbance, and the specific fluorescence intensity is proportional to the product of carbon-normalized absorbance and apparent fluorescence quantum yield. This finding aligns with extrinsic correlations, showing that higher absorbance usually leads to higher fluorescence (SI Text S4.4).

Literature studies have separately demonstrated the utility of SUVA_254_ and FI as surrogates for aromaticity in isolates.^6,7^ However, the transitive property did not hold for these surrogates more broadly, with only 1 of 11 correlations classified as strong (Ozonation subset, Figure 4D), although many correlations (9 of 11) were statistically significant. Notably, the correlation between SUVA_254_ and FI for the GAC subset had the opposite sign (ρ*_s_* = 0.591). Shimabuku et al. (2017) discusses that GAC fractionates DOM in a manner that deviates from the expected correlation, suggesting that FI may be a better indicator of molecular size and SUVA_254_ a better indicator of aromaticity.^41^ The outlier behavior of the GAC subset suggests there are limitations to using optical surrogate-composition interpretations across applications; aromaticity and molecular size are often thought to covary in environmental systems,^11,45,82^ but some treatment applications may be exceptions.

Several of the subsets offer explanations for the lack of correlation between FI and SUVA_254_. For example, the Wastewater subset has a systematically higher FI compared to the Whole-Water subset but similar SUVA_254_ values (Figure 4B, Tables S7 and S13). The GAC subset has a significant positive correlation between FI and SUVA_254_, but the slope is notably shallow. Changes in FI for the GAC subset are small compared to changes in SUVA_254_, especially when compared to other physicochemical processes like coagulation. The inverse relationship between SUVA_254_ and FI in the Coagulation subset is significant with a steep slope but not classified as strong (ρ*_S_* = −0.65). Overall, these examples illustrate that SUVA_254_ and FI are likely probing similar but not completely overlapping aspects of DOM composition. Since SUVA_254_ and FI were proposed as surrogates for aromaticity using HPOA or fulvic acid isolates, one possible explanation is that other structural moieties not recovered during isolation impact FI and SUVA_254_.^6,7^

Correlations between SUVA_254_ and β/α were moderately stronger for Natural/Whole-Waters and Natural/Isolate subsets than relationships between SUVA_254_ and FI (Figure 4D,H). This trend was corroborated by results from prior studies (Figure S38); SUVA_254_ exhibited a significant correlation to β/α in three of the four literature references (Berg et al. 2023 did not report β/α),^59−62^ whereas only the Kellerman et al. (2018) study (isolates) showed a significant relationship between SUVA_254_ and FI.^59^ Considering the Treated subset, β/α and FI correlations with SUVA_254_ depended on sample context (Figure 4D,H). β/α values for the Wastewater subset had a negative but nonsignificant correlation to SUVA_254_ and fell within the spread of the larger data set, including the Natural/Whole-Water and Natural/Isolate subsets. In contrast, FI values for the Wastewater subset were not correlated to SUVA_254_ and systematically higher than the Natural/Whole-water subset. These results indicate that β/α and SUVA_254_ may be providing similar compositional information, with greater overlap than FI, especially for untreated aquatic DOM. However, unlike FI or SUVA_254_, there is a lack of direct comparison of β/α to DOM aromaticity (e.g., via FT-ICR MS or ^13^C NMR), representing an opportunity for future research.

Correlations between SUVA_254_ and HIX were heavily influenced by regions of high HIX sensitivity (HIX < 0.7) dominated by surface water samples treated by preoxidation then coagulation (Figure 4K). The two HIX regions are separated by SUVA_254_ values near 2 L mg_C_^–1^ m^–1^, below which HIX declined precipitously and above which there is a decreasing sensitivity of HIX to increasing SUVA_254_. This trend aligns with prior reports showing that HIX, as reported on the 0–1 scale, loses sensitivity at the upper end.^83^ Interestingly, the entire range of SUVA_254_ values is observed for samples having a HIX greater than 0.8. The lack of correlation between SUVA_254_ and HIX contrasts with a prior study showing that HIX, *S*_275–295_, and SUVA_254_ were all associated with aromatic-rich formulas in van Krevelen space for DOM samples from the Florida Everglades.^80^

## 4 Environmental Implications

Optical surrogates have been widely used to study DOM composition and fate in natural and engineered systems, but the current state of knowledge is limited by (1) the potential misapplication of optical surrogates in unjustified contexts, (2) the disparate studies investigating treated or natural samples but often not both, and (3) whether optical surrogates thought to describe the same compositional information are transitive. Results from the meta-analysis presented here lead to three implications that address these knowledge gaps.

First, there is substantial overlap in the range of surrogate values (especially fluorescence-based surrogates) for both natural and treated samples, with natural samples spanning similar or slightly smaller ranges. This overlap implies that the DOM compositional gradients that govern the variability in optical surrogates can be generated in both natural and engineered systems. These data provide justification for applying optical surrogates, originally developed in the context of natural systems, to engineered treatment systems. However, the interpretation may not be appropriate (e.g., coagulation increases *β/α* but “freshness” does not change). A pattern of deviations in the Wastewater subset suggests that effluent organic matter may not follow conventional interpretations, which suggests that additional research is needed to develop interpretations applicable to the growing field of water reuse.

Second, there is a lack of reciprocity between optical surrogates proposed to describe the same property, such as molecular size (SUVA_254_ and E2:E3)^8,73^ or aromaticity (SUVA_254_ and FI).^6,7^ This observation implies that one or more other DOM physicochemical properties influence optical surrogates apart from aromaticity or molecular weight. Possible explanations include the amount of aliphatic carbon acting as “spacers” between chromophoric groups,^84^ DOM free radical content,^85^ and the abundance of charge-transfer interactions.^21,86,87^ Future research is needed to explore these explanations.

Finally, the meta-analysis data set shows that carbon-normalized optical surrogates tend to correlate well to one another but not to surrogates derived from ratios of intensities. This difference implies that the compositional characteristics governing carbon-normalized surrogates may have less overlap with those that govern the spectral shape. As a consequence, there is still value in measuring DOC concentrations in studies intended to use optical analyses to characterize DOM composition, fate, and reactivity.

In addition to addressing these knowledge gaps, the meta-analysis leads us to make several recommendations about which optical surrogates should be prioritized in future work. These recommendations are based on the newly developed workflow for assessing data quality prior to optical surrogate reporting, correlations between optical surrogates, and the expected ease of measurement in different contexts (laboratory- vs field-based studies):

- **Specific absorbance**: The lack of strong and significant correlations between specific absorbance and spectral shape surrogates highlights that both should be measured in DOM studies. We recommend continued prioritization of SUVA_254_ in laboratory studies, with special attention paid to potentially interfering concentrations of iron and nitrate. Field measurement of SUVA_254_ may be possible if online organic carbon analyzers are available.
- **Absorbance spectral tailing**: While several slope-related surrogates are available, we recommend either E2:E3 or *S*_300–600_. E2:E3 requires measurement at only two wavelengths, is more amenable to field sensors, and avoids use of nonlinear solving methods. We recommend *S*_300–600_ over *S*_275–295_ or *S*_350–400_ for two reasons. First, *S*_300–600_ exhibited tight correlations to E2:E3 and thus likely describes similar characteristics. Second, *S*_300–600_ is less sensitive to absorption quality control cutoffs compared to *S*_275–295_ and *S*_350–400_ since its calculation does not rely on linear regression fitting.
- **The extent of red-shifted fluorescence**: Most relationships between fluorescence- and absorbance-based surrogates had a low frequency of strong correlations. Overall, this suggests that fluorescence surrogates add value and potentially describe unique compositional characteristics. Based on the meta-analysis, we suggest prioritization of FI and *β/α* in future DOM studies. Both indices are shown, across a broad range of samples, to be correlated to the peak emission maximum at their respective excitation wavelengths (i.e., red-shifted fluorescence). HIX is more likely to be impacted by low-fluorescing samples at low emission wavelengths and more challenging to implement in field sensors due to inner filter effects. Comparatively, FI and *β/α* use discrete wavelengths, without integration, and FI would be less susceptible to biases introduced by inner filtering. In situ measurements of FI have already been implemented using different cutoff filters,^88^ and technology is expected to improve using charge-coupled devices.
- **Field sensors**: In the context of using optical surrogates for low-resource data acquisition or in situ sensors, we suggest that practitioners strategically select optical surrogates not impacted by potentially interfering constituents. For example, studies in high-nitrate agricultural contexts could consider using fluorescence-based surrogates at higher excitation wavelengths such as FI. Nitrate is not expected to quench DOM fluorescence but could increase inner filter effects. Interferences from iron may be more challenging to address, given its broad UV absorption band and fluorescence quenching. Given the challenge of optical interference, there is an opportunity to develop and implement DOM characterization methods not subject to these interferences.

This study shows examples of where optical surrogate correlations do not align across sample subsets, highlighting that no single surrogate can completely describe all aspects of the DOM composition. Taken as a whole, these examples point to the need for future work to better understand how DOM composition impacts optical surrogates. Paramount are continued efforts that pair optical surrogates with independent measures of composition, both those that have been used traditionally (i.e., aromaticity and molecular weight) and those that describe new aspects of composition (e.g., the extent of interacting chromophores).

## Data Availability

The codes associated with the quality control methodology for optical surrogates will be openly available in Zenodo at doi.org/10.5281/zenodo.10895766 and the linked GitHub repository.

## Supporting Information Available

Supporting tables and figures; definitions and calculation methods of optical surrogates, including quality control criteria; overview of the meta-analysis data set and comparison to literature data; supplementary correlation analyses; comparison between different spectral slope wavelength ranges (PDF)
