# Supplementary material for: Meta-Analysis of Optical Surrogates for the Characterization of Dissolved Organic Matter
Source: Environ Sci Technol. 2024 Apr 19;58(17):7380–92. doi: 10.1021/acs.est.3c10627 (PMC11064222; doi:10.1021/acs.est.3c10627)
Supplement: Supplementary file 1 — es3c10627_si_001.pdf [file es3c10627_si_001.pdf]

Supporting Information for

Meta-Analysis of Optical Surrogates for the Characterization of Dissolved Organic Matter

Julie A. Korak[a,b*] and Garrett McKay[c*]

[a]Department of Civil, Environmental, and Architectural Engineering and [b]Environmental Engineering Program, University of Colorado, Boulder, CO 80303

[c]Zachry Department of Civil & Environmental Engineering, Texas A&M University, College Station, TX 77843

***Author contact information**

Julie A. Korak
E-mail: Julie.Korak@colorado.edu;
Orcid ID: https://orcid.org/0000-0001-9355-2426
Phone: 303.735.4895

Garrett McKay
E-mail: gmckay@tamu.edu
Orcid ID: https://orcid.org/0000-0002-6529-0892
Phone: 979.458.6540

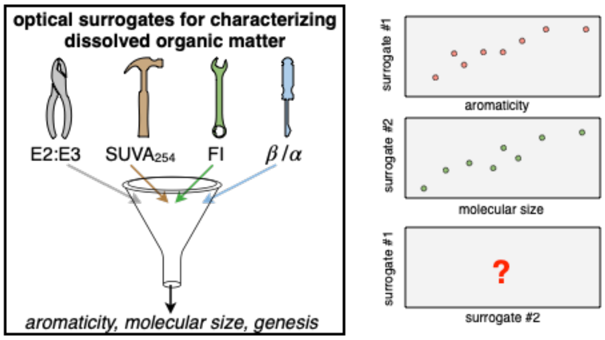

Number of pages: 69

Number of text sections: 5

Number of tables: 17

Number of figures: 39

Table of Contents

List of Tables 3

List of Figures 4

S 1 Optical Surrogate Definitions 6

S1.1 Absorbance Surrogates 6

S1.2 Fluorescence Surrogates 10

S 2 Overview of Dataset 17

S 3 Comparison to Literature Data 39

S 4 Correlation Analysis 44

S4.1 Optical surrogate correlations within the primary dataset 44

S4.2 Correlations between $S_{275-295}$, $S_{350-400}$, and S_R 49

S4.3 Additional correlations between intrinsic optical surrogates 50

S4.4 Additional correlations between extrinsic optical surrogates 58

S4.5 Literature data 61

S 5 References 64

List of Tables

Table S1. Definitions and quality control criteria for absorbance-based optical surrogates. 7

Table S2. Definition and quality control criteria for fluorescence-based optical surrogates 11

Table S3. Calculation method for CV_{RMSD} for each fluorescence-based optical surrogate 13

Table S4. Index of studies contributing absorbance spectra and EEMs to meta-analysis. 18

Table S5. Index of studies contributing absorbance spectra and EEMs to meta-analysis. 19

Table S6. Summary statistics of extrinsic optical surrogates differentiating whole-waters from isolates 22

Table S7. Summary statistics of intrinsic absorbance surrogates differentiating whole-waters from isolates 23

Table S8. Summary statistics of intrinsic fluorescence surrogates differentiating whole-waters from isolates 24

Table S9. Summary statistics of extrinsic optical surrogates differentiating treated and natural samples 25

Table S10. Summary statistics of intrinsic absorbance surrogates differentiating treated and natural samples 26

Table S11. Summary statistics of intrinsic fluorescence surrogates differentiating treated and natural samples 27

Table S12. Summary statistics of extrinsic optical surrogates differentiating aquatic sources... 28

Table S13. Summary statistics of intrinsic absorbance surrogates differentiating aquatic sources 29

Table S14. Summary statistics of intrinsic fluorescence surrogates differentiating aquatic sources 30

Table S15. Summary statistics of extrinsic optical surrogates differentiating treatment perturbation 31

Table S16. Summary statistics of intrinsic absorbance surrogates differentiating treatment perturbation 33

Table S17. Summary statistics of intrinsic fluorescence surrogates differentiating treatment perturbation 35

List of Figures

Figure S1. Schematic of optical surrogates overlaid on example spectra. 6

Figure S2. Cumulative distribution of background noise for absorbance spectra (n=612). 8

Figure S3. Comparison of two E2:E3 calculation methods. 9

Figure S4. Impact of QC criteria on spectral slope calculations. 10

Figure S5. Cumulative distribution of $CVRMSD$ for each fluorescence based optical metric. 13

Figure S6. Example of samples retained and eliminated based on $CVRMSD$ 14

Figure S7. Summary of samples retained after screening for high relative noise ($CVRMSD$) and eliminating the upper and lower 0.5 percentile. 15

Figure S8. Comparison of BIX to β/α. 16

Figure S9. Calculation of absorbance at 254 nm (UV_{254}) as a function of iron (III) and nitrate concentrations. 37

Figure S10. Comparison of $SUVA_{254}$ values between study subsets (Treated and Natural) and literature references. 40

Figure S11. Comparison of E2:E3 values between study subsets (Treated and Natural) and literature references. 40

Figure S12. Comparison of $S_{275-295}$ values between study subsets (Treated and Natural) and literature references. 41

Figure S13 Comparison of S_R values between study subsets (Treated and Natural) and literature references. 41

Figure S14 Comparison of HIX values between study subsets (Treated and Natural) and literature references. 42

Figure S15 Comparison of β/α values between study subsets (Treated and Natural) and literature references. 42

Figure S16 Comparison of FI values between study subsets (Treated and Natural) and literature references. 43

Figure S17. Heatmap of Spearman rho values (ρS) for all samples in dataset. 44

Figure S18. Heatmap of Spearman rho values (ρS) for all Natural samples in dataset. 45

Figure S19. Heatmap of Spearman rho values (ρS) for all Treated samples in dataset. 45

Figure S20. Heatmap of Spearman rho values (ρS) for all Isolate (Natural) samples in dataset. 46

Figure S21. Heatmap of coefficient of determination (R^2) values for all samples in dataset. 47

Figure S22. Heatmap of coefficient of determination (R^2) values for all Natural samples in dataset. 47

Figure S23. Heatmap of coefficient of determination (R^2) values for all Treated samples in dataset. 48

Figure S24. Heatmap of coefficient of determination (R^2) values for Isolate (Natural) samples in dataset. 48

Figure S25. Relationships between the spectral slope between 275-295 nm ($S_{275-295}$) and other absorbance-based surrogates. 49

Figure S26. Relationships between the specific ultraviolet absorbance at 254 nm ($SUVA_{254}$) and other absorbance-based surrogates. 50

Figure S27. Relationships between the wavelength of maximum emission at 370 nm excitation ($\lambda max370nm$) and fluorescence surrogates. 51

Figure S28. Relationships between the wavelength of maximum emission at 310 nm excitation ($\lambda max310nm$) and fluorescence surrogates. 52

Figure S29. Relationships between the wavelength of maximum emission at 254 nm excitation ($\lambda max254nm$) and fluorescence surrogates. 53

Figure S30. Relationships between specific peak intensities........ 54

Figure S31. Relationships between the specific ultraviolet absorbance (SUVA) at different wavelengths. 55

Figure S32. Relationships between E2:E3 and fluorescence surrogates. 56

Figure S33. Relationships between the specific ultraviolet absorbance (SUVA) at 370 nm and fluorescence surrogates........ 57

Figure S34. Relationships between UV absorbance at different wavelengths. 58

Figure S35. Relationship between UV absorbance at 254 nm and extrinsic fluorescence surrogates. 59

Figure S36. Relationships between fluorescence peak intensities. 60

Figure S37. Relationship between literature-derived spectral slope between 275-295 ($S_{275-295}$) and other absorbance-based optical surrogates........ 61

Figure S38. Relationship between literature-derived specific ultraviolet absorbance (SUVA) at 254 nm and other optical surrogates. 62

Figure S39. Relationship between literature-derived fluorescence index (FI) and other fluorescence-based surrogates........ 63

S 1 Optical Surrogate Definitions

This section documents the methods to calculate the optical surrogates, including an explicit definition for each surrogate and how quality control (QC) criteria were applied to the dataset. Visual representations of common surrogates are shown in **Figure S1**. Importantly, a general methodology for developing quality control thresholds is presented in this section. Quality control thresholds were defined based on spectral noise. Recognizing that there may also be outliers in the dataset that are unrelated to spectral noise, the upper and lower 0.5% of calculated values for each optical surrogate were excluded from correlations. The specific quality control thresholds for each optical surrogate are specific to the instruments, methods, and sample context. Another study could apply the same methodology but identify different quantitative thresholds.

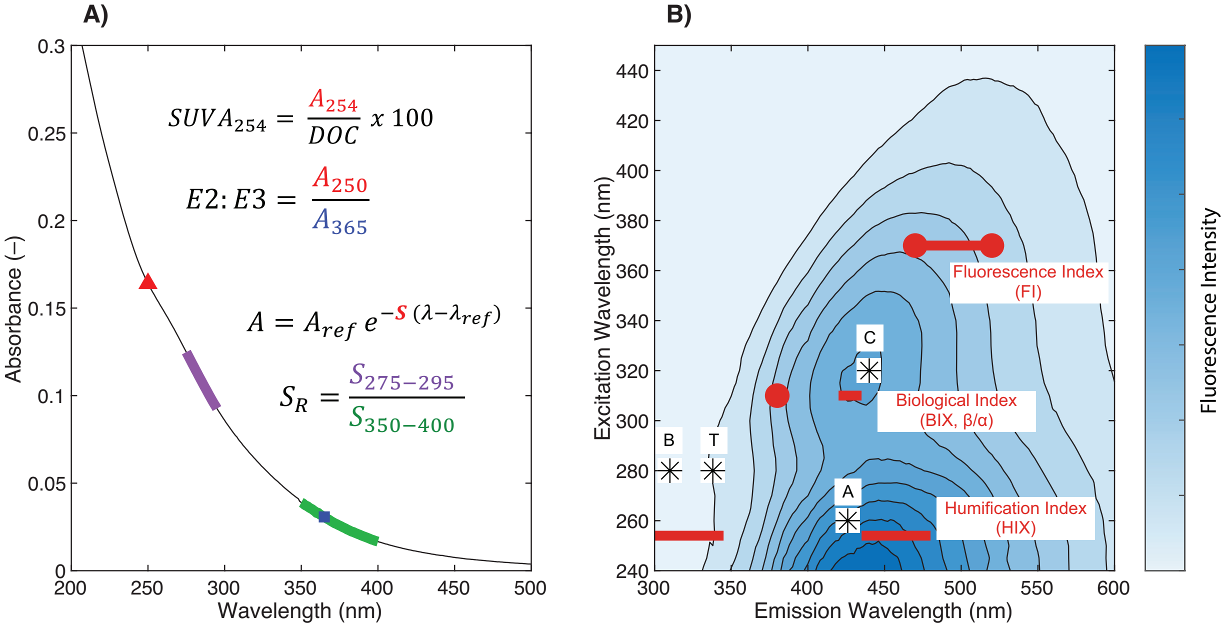

Figure S1. Schematic of optical surrogates overlaid on example spectra. A) Visual representation of $SUVA_{254}$, E2:E3, spectral slope in two regions, and slope ratio (S_R). B) Annotations of wavelengths used to calculate fluorescence indices (humification, biological, and fluorescence) and the location of wavelengths used for peak picking.

S1.1 Absorbance Surrogates

Absorbance-based optical surrogates were calculated according to **Table S1**, which includes specific ultraviolet absorbance at various wavelengths, absorbance ratios, spectral slopes, and the spectral slope ratio. Absorbance spectra were baseline corrected and normalized to a 1 cm pathlength. The baseline was determined on a sample-by-sample basis by averaging absorbance values between 650 and 700 nm, where the dissolved organic matter (DOM) absorbance is near zero with high relative noise. Quality control criteria were applied to each sample as described in this section.

Table S1. Definitions and quality control criteria for absorbance-based optical surrogates. All absorbance quality control (QC) criteria are based on absorbance as-measured at the cuvette pathlength.

Metric	Examples	Calculation	QC Criteria	References
UV_i	UV_{254}<br>UV_{280}<br>UV_{320}<br>UV_{370}	Absorbance at wavelength *i* (A_i) normalized to a pathlength of 1 cm and baseline corrected	$A_i > 0.005$	Shapiro (1957)[1]
$SUVA_i$	$SUVA_{254}$<br>$SUVA_{280}$<br>$SUVA_{320}$<br>$SUVA_{370}$	$SUVA_i = \frac{A_i}{DOC} \times 100$	$A_i > 0.005$<br>DOC > 0.5 mg/L	Traina et al. (1990)[2], Chin et al. (1994)[3], Peuravuori and Pihlaja (1997),[4] Weishaar (2004)[5]
$E_i{:}E_j$	E2:E3<br><br>E4:E6	$E2{:}E3 = \frac{A_{250\,nm}}{A_{365\,nm}}$<br><br>$E4{:}E6 = \frac{A_{465\,nm}}{A_{665\,nm}}$	$A_{365\,nm} > 0.005$<br><br>$A_{665\,nm} > 0.005$	De Haan (1983)[6]
$S_{i\text{-}j}$	$S_{300\text{-}600}$<br>$S_{300\text{-}650}$<br>$S_{300\text{-}700}$	**Non-linear form:**<br>$A_\lambda = A_{\lambda_{ref}} e^{-S(\lambda-\lambda_{ref})}$<br>where<br>S is the spectral slope<br>λ_{ref} is the reference wavelength of 350 nm		Stedmon et al. (2000)[7]
	$S_{275\text{-}295}$<br>$S_{350\text{-}400}$	**Linear form:**<br>$\log\left(\frac{2.303\,A_\lambda}{0.01\,\frac{m}{cm}}\right) = S \cdot \lambda + b$<br>where<br>b is the intercept (not reported)	$A_{295\,nm} > 0.005$<br>$A_{400\,nm} > 0.005$	Helms 2008[8]
S_R	S_R	$S_R = \frac{S_{275-295}}{S_{350-400}}$	$A_{400\,nm} > 0.005$	Helms 2008[8]

Absorbance Quality Control. Low optical densities approaching baseline noise could impact quantitation of absorbance surrogates. This issue has been recognized previously for E4:E6, where the optical density at 665 nm is usually low.[9] Thus, surrogates derived from absorbance at a single wavelength (e.g., $SUVA_{254}$) or a ratio of two wavelengths (e.g., E2:E3) were calculated only if the as-measured absorbance at all relevant wavelengths exceeded a quality control threshold. To address this concern, a quality control threshold was determined based on an analysis of noise at the long wavelengths (>600 nm), which are used to baseline correct the spectrum. For each absorbance spectra in this dataset, the standard deviation of the baseline signal was calculated across a 25 to 50 nm range at wavelengths above 600 nm. The 95th percentile for background noise for this dataset was 0.00028 (**Figure S2**). The quality control threshold for calculating absorbance-based surrogates is approximately 20 times the background noise, yielding a 0.005 threshold. This minimum absorbance criterion is applied to the as-measured absorbance, accounting for different cuvette pathlengths. Samples with absorbance >1.5 (n=9) were removed due to a high likelihood of non-DOM chromophores (e.g., nitrate) and the upper limit of the linear operating range of the spectrophotometers. Quality control criteria are instrument-specific; this approach could be applied by other researchers to determine appropriate absorbance criteria for other datasets.

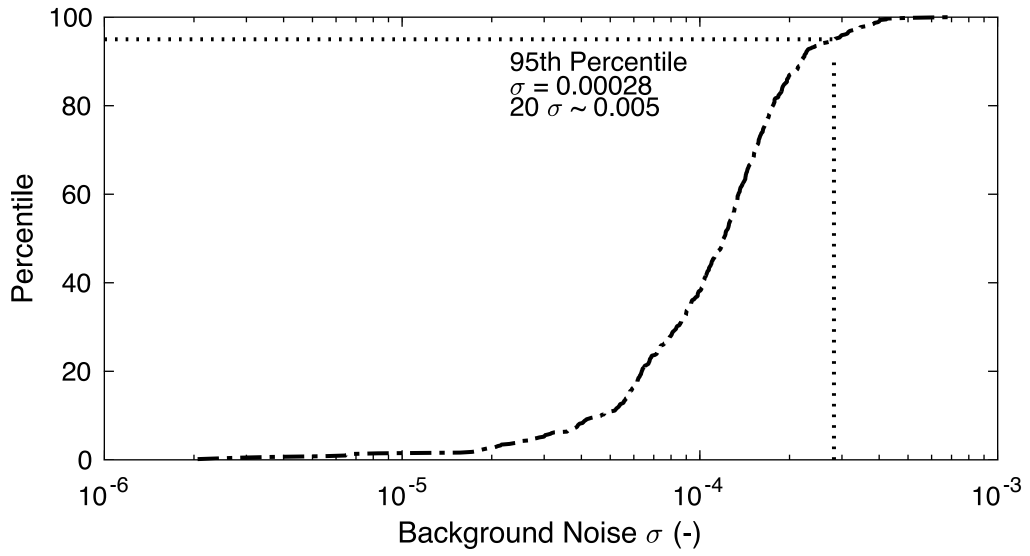

Figure S2. Cumulative distribution of background noise for absorbance spectra (n=612). Horizontal line indicates the 95th percentile for background noise.

Specific Ultraviolet Absorbance and Dissolved Organic Carbon. Absorbance at four wavelengths (254, 280, 320, and 370 nm) was extracted from spectra, multiplied by 100 (to convert cm^{-1} to m^{-1}), and divided by the dissolved organic carbon (DOC) concentration to calculate the specific ultraviolet absorbance (SUVA) in units of L mg_C^{-1} m^{-1}. A minimum DOC criterion was also applied (DOC > 0.5 mg_C/L) to avoid measurements close to method reporting limits imparting disproportionately high propagated error. A threshold of >0.5 mg_C/L was chosen to be greater than the acceptable blank concentration (0.35 mg_C/L) of EPA Method 415.3 and more than 3 times the historical observed background concentrations on the carbon analyzers (M5310C, GE or TOC-V_{CSH}, Shimadzu) at CU Boulder.

Absorbance Ratios. Two different absorbance ratios were calculated. The first, E2:E3, was calculated from the ratio of absorbances 250 nm to 365 nm, following the approach in the earliest known studies.[6,10] However, since some studies use absorbance at 254 nm in the numerator,[11-13] both approaches were calculated. There is an average bias of 7% (slope = 0.933) between the two methods (**Figure S3**). Although the supplemental MATLAB code calculates both methods, only the former method (A_{250}/A_{365}) is presented in the main manuscript. Additionally, E4:E6 was calculated. For quality control, the ratio was only reported if the absorbance at the longer wavelength (i.e., lower absorbance) exceeded 0.005.

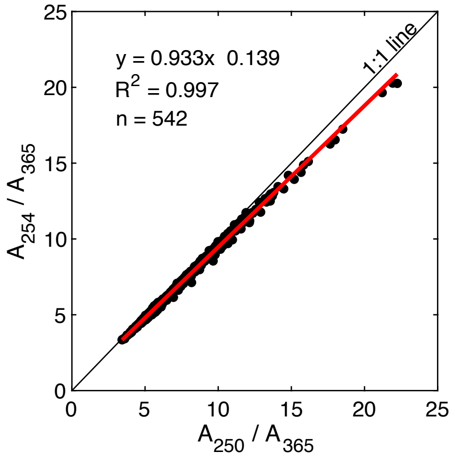

Figure S3. Comparison of two E2:E3 calculation methods. Solid black line is 1:1 line. The red line shows a linear regression relating both calculation methods.

Spectral slope. Spectral slope (S) was calculated between a defined wavelength range (i–j) following two approaches. $S_{275\text{-}295}$ and $S_{350\text{-}400}$ were calculated using the natural-log linearized absorption coefficients following previous studies.[8,14,15] The spectral slope ratio (S_R) was calculated from the ratio of $S_{275\text{-}295}$ to $S_{350\text{-}400}$. For quality control, S was only reported if the as-measured absorbance at the maximum wavelength in each wavelength range (e.g., 295 or 400 nm, respectively) exceeded 0.005.

Spectral slopes across broader wavelength ranges ($S_{300\text{-}600}$, $S_{300\text{-}650}$, and $S_{300\text{-}700}$) were calculated by fitting a non-linear exponential decay function using an iterative solver in MATLAB (fminsearch). Spectral slope was calculated using three different upper limits for the wavelength range based on previous studies and instrument settings for data in this meta-analysis. Previous studies have reported both 300–700 nm[8,14] and 300–650 nm.[16,17] $S_{300\text{-}600}$ was also included because some absorbance spectra in this dataset were only measured up to 600 nm.

Figure S4C shows $S_{300\text{-}600}$ and $S_{300\text{-}700}$ are generally in good agreement. However, Twardowski et al. (2004) showed that the calculated spectral slope depends on the starting and ending wavelengths and that low absorbing samples with elevated relative noise can also impact spectral slope calculations.[18] As a quality control strategy, $S_{i\text{–}j}$ was only reported if the as-measured absorbance at the minimum wavelength (i.e., 300 nm) exceeded the quality control threshold (0.005). This approach guaranteed that at least one absorbance measurement within the wavelength range exceeded the quality control threshold.

Another quality control criterion that was considered for $S_{i\text{–}j}$, but ultimately not implemented, related to the fraction of absorbance values in each spectra that exceeded the quality control threshold (0.005). **Figure S4A** shows that the dataset has a continuous and near uniform distribution of samples based on the fraction of each absorbance spectrum above 0.005. There is no clear discontinuity in the dataset. It was ultimately decided not to adopt a threshold based on the fraction of each absorbance spectra that needs to exceed 0.005, because it would bias the dataset to samples with lower spectral slopes (**Figure S4B**). Since 70% of samples were measured using a 1 cm cuvette, low as-measured absorbance (<0.005) in the visible range (>400 nm) is expected for many aquatic environments. **Figure S4C** shows that there is good agreement between spectral slopes regardless of the fractional threshold for $S_{300\text{–}700}$. Bias between $S_{300\text{–}600}$ and $S_{300\text{–}700}$ is introduced when the fraction of the spectra above 0.005 exceeds 0.7, which is a small portion of the dataset (<30%). **Figure S4D** shows that if a quality control criterion that limits

the reported values were applied, correlations with other optical properties would be biased to a narrow range representing a narrow range of potential underlying chemical characteristics. Therefore, a liberal quality control criterion was applied requiring only the absorbance at 300 nm to exceed 0.005.

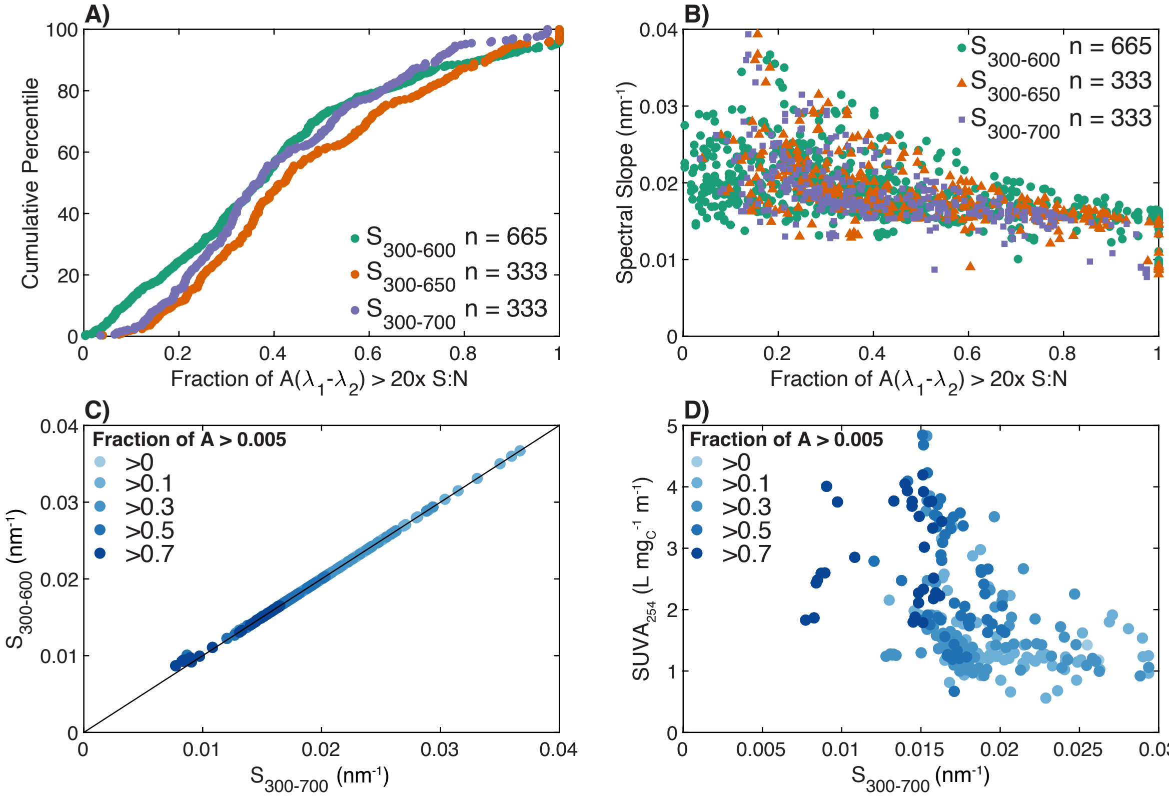

Figure S4. Impact of QC criteria on spectral slope calculations: A) Cumulative distribution of the fraction of spectra >0.005 for $S_{300–700}$, B) Spectral slope as a function of the fraction of absorbance spectra >0.005 as measured, C) Comparison of $S_{300–600}$ vs. $S_{300–700}$ plotted by the fraction of spectra above 0.005 threshold, and D) $SUVA_{254}$ vs. $S_{300–700}$ plotted by the fraction of spectra above 0.005 threshold.

S1.2 Fluorescence Surrogates

The calculation method for each fluorescence-based optical surrogate is summarized in **Table S2**. All fluorescence surrogate calculations used corrected fluorescence intensities extracted from excitation-emission matrices (EEMs) at specific wavelength combinations. If the data acquisition parameters did not match the emission wavelengths of the surrogate, a substitute wavelength within 1 nm was identified, if available. An example where a substitution was made is the biological index (β/α), because emission intensity was measured using even wavelength increments but the metric searches for an odd wavelength (435 nm). As another example, non-integer emission wavelengths may be measured on spectrofluorometers that use charge-coupled device (CCD) detectors (e.g., Horiba Aqualog) but, for practical purposes, data are typically interpolated to integer wavelengths in post-processing. Specific details on the calculation of each optical surrogate and quality control approaches are documented in this section.

Table S2. Definition and quality control criteria for fluorescence-based optical surrogates

Metric	Examples	Example Calculation	Ref.
Humification Index	HIX	$HIX = \frac{\sum_{\lambda_{em,3}}^{\lambda_{em,4}} I(254\,nm\,,\ \lambda_{em})}{\sum_{\lambda_{em,1}}^{\lambda_{em,2}} I(254\,nm\,,\lambda_{em}) + \sum_{\lambda_{em,3}}^{\lambda_{em,4}} I(254\,nm\,,\lambda_{em})}$<br>where<br>$\lambda_{em,1} = 300\,nm$<br>$\lambda_{em,2} = 345\,nm$<br>$\lambda_{em,3} = 435\,nm$<br>$\lambda_{em,4} = 480\,nm$	Ohno 2002[13]<br>Zsolnay et al. (1999)[19]
Biological/ Freshness Index	β/α<br><br>BIX	$\beta/\alpha = \frac{I(310\,nm,\ 380\,nm)}{\max\left(I(310\,nm,\ 420-435\,nm)\right)}$<br><br>$BIX = \frac{I(310\,nm,\ 380\,nm)}{I(310\,nm,\ 430\,nm)}$	Parlanti et al. (2000)[17]<br>Wilson and Xenopoulos (2009)[20]<br>Huguet et al. (2009)[21]
Fluorescence Index	FI	$FI = \frac{I(370\,nm,\ 470\,nm)}{I(370\,nm,\ 520\,nm)}$	Cory and McKnight (2010)[22]<br>McKnight et al. (2001) [23]
Maximum emission wavelength	$\lambda_{em,max}$ at λ_{ex} = 254 nm<br><br>$\lambda_{em,max}$ at λ_{ex} = 310 nm<br><br>$\lambda_{em,max}$ at λ_{ex} = 370 nm	$\lambda_{max}(254nm) = \max(I(254\,nm, 300-\ 478\,nm)$<br><br>$\lambda_{max}(310nm) = \max(I(310\,nm, 310\,-\ 590\,nm)$<br><br>$\lambda_{max}(370nm) = \max(I(370\,nm, 370\,-\ 710\,nm)$	Cory and McKnight (2010)[22]<br>McKnight et al. (2001)[23])<br><br>Parlanti et al. (2000)[17]<br>Wilson and Xenopoulos (2009)[20]
Specific Peak Intensities	Sp. Peak A	$SpA = \frac{I(260\,nm,\ 426\,nm)}{DOC}$	Alberts and Takács (2004)[24]<br>Jaffé et al. (2004)[25]<br>Hudson et al. (2007)[26]<br>Korak et al. (2014)[27]
	Sp. Peak B	$SpB = \frac{I(280\,nm,\ 310\,nm)}{DOC}$	
	Sp. Peak C	$SpC = \frac{I(320\,nm,\ 440\,nm)}{DOC}$	
	Sp. Peak T	$SpT = \frac{I(280\,nm,\ 338\,nm)}{DOC}$	
where<br>λ_{ex} is the excitation wavelength in nm<br>λ_{em} is the emission wavelength in nm<br>$I(\lambda_{ex}, \lambda_{em})$ is the corrected fluorescence intensity at λ_{ex} and λ_{em} in Raman units (RU)<br>DOC is the dissolved organic carbon concentration in mg_C/L			

Fluorescence Quality Control. Quality control criteria for fluorescence data compared the instrumental noise relative to the corrected signal intensity in wavelength regions relevant for each optical surrogate. This approach contrasts with the method used for absorbance data, which followed a more traditional assessment of signal-to-noise. However, a signal-to-noise approach cannot be applied to *corrected* fluorescence intensities, because the corrected fluorescence intensity is a function of the raw fluorescence signal, wavelength-dependent instrument-specific correction factors, and the sample- and wavelength-specific inner filter correction (IFC) factor. For example, the raw fluorescence intensity for two samples could be identical, but the corrected fluorescence intensity for one sample could be higher due to higher sample absorbance and larger IFC factors. Setting an intensity-based quality control threshold is not possible unless the raw fluorescence data is available. However, the typical workflow on instruments that simultaneously measure fluorescence and absorbance (e.g., Horiba Aqualog) exports corrected

fluorescence data, which incorporates instrument-specific correction factors, blank subtraction, Raman normalization, and IFC factors. Therefore, the approach applied in this work assessed the relative noise using the corrected fluorescence EEMs, not the raw intensities, to be compatible with a range of workflows. Relative noise was assessed using the coefficient of variance based on the root mean square deviation (CV_{RMSD}), which is the ratio of the root mean square deviation (RMSD) to the mean fluorescence intensity. This section details the calculation method and quality control criteria for fluorescence based optical surrogates.

For each fluorescence surrogate, an emission spectrum at the relevant excitation and emission wavelengths was smoothed using a locally weighted scatterplot smoothing technique (LOWESS). The range of emission wavelengths bracketed the specific wavelength(s) needed to calculate the optical surrogate. The smoothing span for each parameter was 0.2, except for Peaks B and T where the span was increased to 0.3 due to the smaller emission wavelength range (**Table S3**). By comparing the measured data to the smoothed spectra, the RMSD was calculated using Equation S1. The coefficient of variation based on the RMSD (Equation S2) compares the RMSD to the mean fluorescence intensity of the emission spectra.

$$RMSD = \sqrt{\frac{\sum_{\lambda_{em,1}}^{\lambda_{em,2}} \left(I(\lambda_{ex},\lambda_{em}) - I_{smooth}(\lambda_{ex},\lambda_{em})\right)^2}{n}} \qquad \text{Equation S1}$$

$$CV_{RMSD} = \frac{RMSD}{mean\left(I(\lambda_{ex}, \lambda_{em,1} - \lambda_{em,2})\right)} \qquad \text{Equation S2}$$

where,

$RMSD$ is the root mean square deviation in RU
λ_{ex} is the excitation wavelength in nm
$\boldsymbol{\lambda_{em,1} - \lambda_{em,2}}$ is the emission wavelength range in nm
$I(\lambda_{ex}, \lambda_{em})$ is the corrected fluorescence intensity at λ_{ex} and λ_{em} in RU
$I_{smooth}(\lambda_{ex}, \lambda_{em})$ is the smoothed fluorescence intensity at λ_{ex} and λ_{em} in RU
n is the number of fluorescence intensity observations in the RMSD calculation
CV_{RMSD} is the coefficient of variation based on the RMSD
$mean(\;\;)$ is the mean fluorescence intensity of the emission spectra

Table S3. Calculation method for CV_{RMSD} for each fluorescence-based optical surrogate

**Optical Property**	λ_{ex} **(nm)**	$\lambda_{em,1}-\lambda_{em,2}$ **(nm)**	**LOWESS span**	**QC Criteria**
HIX	254	300–480	0.2	CV_{RMSD} < 0.04
β/α<br>BIX<br>$\lambda_{em,max}$ at λ_{ex} = 310 nm	310	380–435	0.2	CV_{RMSD} < 0.008
FI<br>$\lambda_{em,max}$ at λ_{ex} = 370 nm	370	470–520	0.2	CV_{RMSD} < 0.009
SpA	260	376–476	0.2	CV_{RMSD} < 0.02<br>DOC > 0.5 mg/L
SpB	280	300–320	0.3	CV_{RMSD} < 0.16<br>DOC > 0.5 mg/L
SpC	320	390–490	0.2	CV_{RMSD} < 0.008<br>DOC > 0.5 mg/L
SpT	280	328–348	0.3	CV_{RMSD} < 0.02<br>DOC > 0.5 mg/L

By plotting the cumulative distribution of CV_{RMSD} values for all samples (**Figure S5**), the 95th percentile was used to define the upper limit for acceptable, relative spectral noise. For cases where several surrogates are calculated using fluorescence data in the same wavelength region (e.g., BIX and β/α), the same CV_{RMSD} was set as a threshold for each surrogate. In general, CV_{RMSD} increased as excitation wavelength decreased. Relative noise was greatest for surrogates calculated at excitation wavelengths ≤280 nm, which includes humification index (HIX) and specific peak intensities SpA, SpB, and SpT. Although EEMs typically show higher corrected fluorescence intensities in the Peak A region compared to Peak C, the raw intensity measured on a spectrofluorometer is usually lower for Peak A due to primary inner filtering and lower lamp intensity. The IFC procedure preferentially increases the fluorescence intensity at lower excitation wavelengths to correct for increased sample absorbance. The CV_{RMSD} values were greatest for Peak B, which is due to low raw signal in many samples, the greatest IFCs, and proximity to the Raman peak of water.[27] Examples of spectra for retained and rejected samples are shown in **Figure S6**.

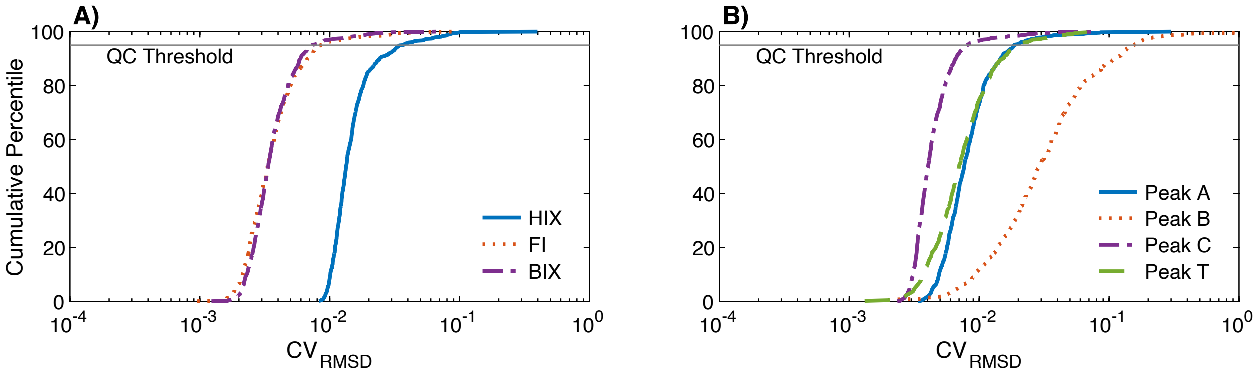

Figure S5. Cumulative distribution of CV_{RMSD} for each fluorescence based optical metric. The horizontal line identifies the 95th percentile, which defined the upper bound of acceptable noise for each optical surrogate.

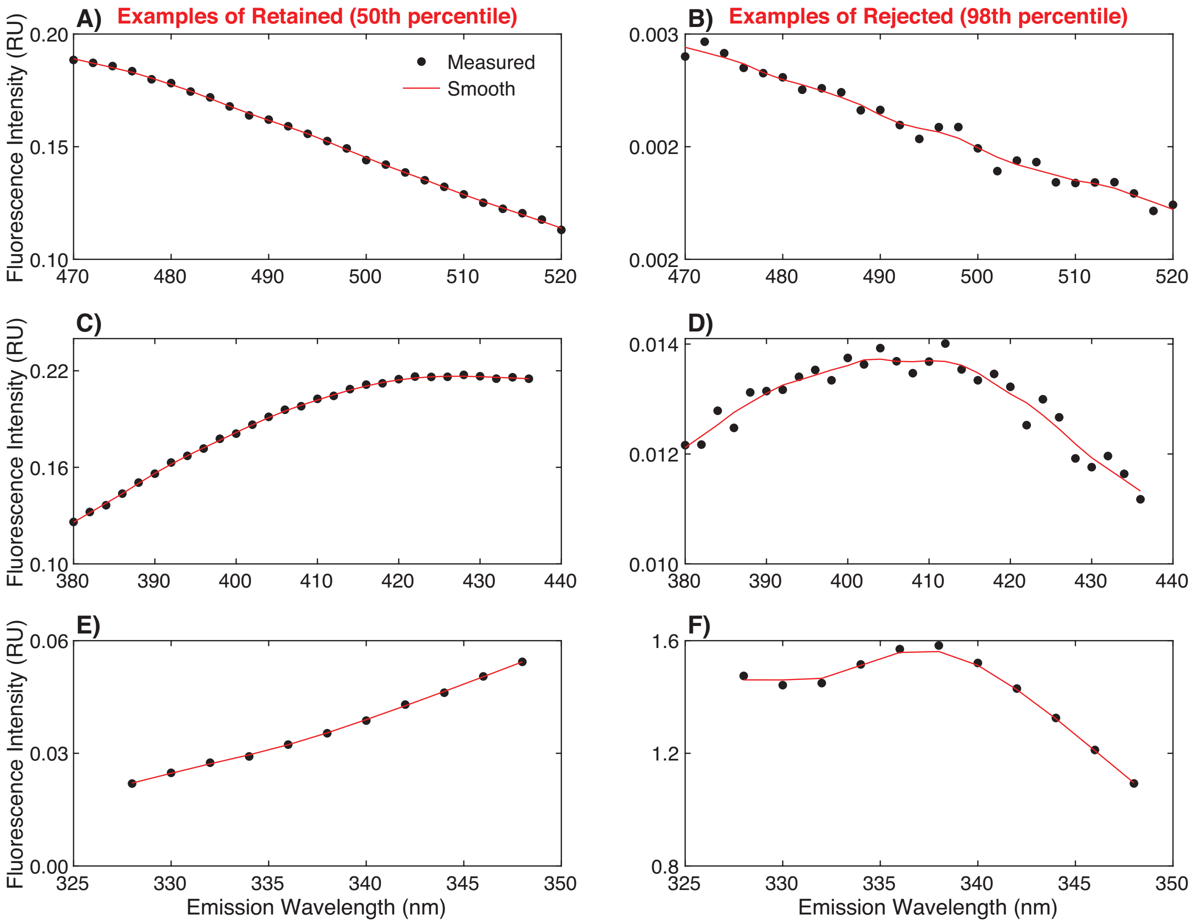

Figure S6. Example of samples retained and eliminated based on CV_{RMSD} for A-B) fluorescence index (FI), C-D) biological/freshness index (β/α), and E-F) specific peak T (SpT) intensity. Subplots A, C, E are examples of samples with a CV_{RMSD} near the 50th percentile of all samples, which would be retained. Subplots B, D, and F are examples of samples at the 98th percentile, which would be rejected.

The CV_{RMSD} thresholds, defined in **Table S3,** were used to reject fluorescence surrogates where the underlying spectra had high relative noise. After samples with high relative noise were rejected, the upper and lower 0.5% of calculated values for each fluorescence surrogate were also rejected to avoid potential outliers leveraging correlations. **Figure S7** shows the distribution of samples that were rejected or retained for each fluorescence surrogate.

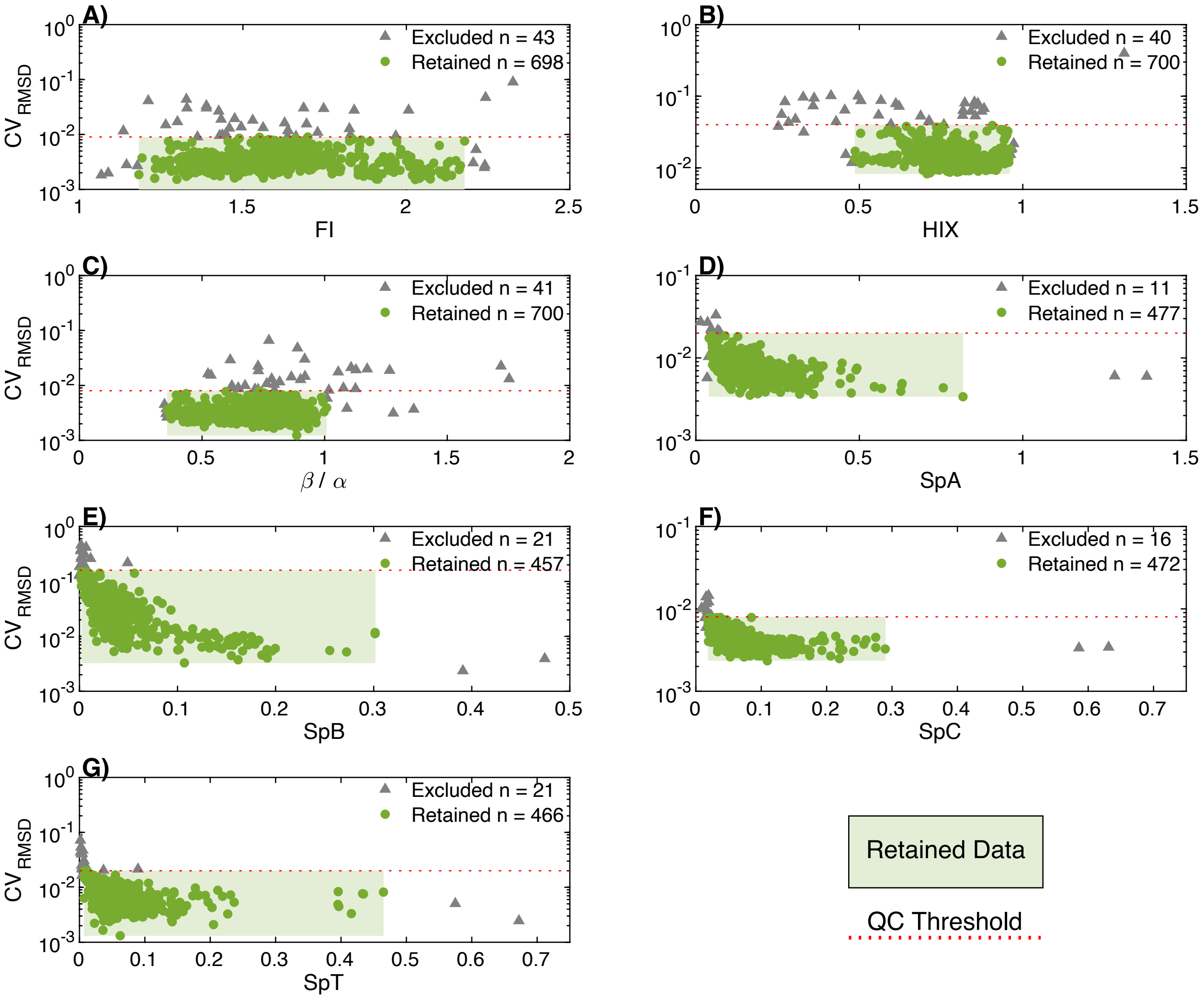

Figure S7. Summary of samples retained after screening for high relative noise (CV_{RMSD}) and eliminating the upper and lower 0.5 percentile.

Humification Index. The humification index (HIX) was calculated following the approach in Ohno (2002),[28] which calculates the ratio of two integrated areas under an emission spectrum. Of the two approaches in Ohno (2002), the approach that includes both integrated areas in the denominator was adopted, because it is less susceptible to noise when fluorescence intensity is low at emission wavelengths between 300 and 345 nm. Many fluorescence EEMs are not measured at an excitation wavelength of 254 nm. In these cases, HIX was calculated using a two-dimensional interpolation using adjacent excitation wavelengths (e.g., 250 and 260 nm) applying the *griddata* function with cubic interpolation, similar to Zepp et al. (2004).[29] The QC criterion rejecting samples with high noise eliminated samples with a broad range of HIX values (**Figure S7B**), including most HIX values <0.5. Therefore, applying the QC criterion decreased the distribution of HIX values in the retained dataset.

Biological/Freshness Index. Also referred to as the freshness index, beta peak relative to alpha peak (β/α), or biological index (BIX), this surrogate has two calculation methods based on excitation at 310 nm.[30,31] Both approaches extract the fluorescence intensity at emission 380 nm for the numerator. β/α, originally proposed by Parlanti et al. (2000)[32] and defined more explicitly by Wilson and Xenopoulos (2009),[20] uses the maximum fluorescence intensity within a range of emission wavelengths (420–435 nm) in the denominator.[20] Alternatively, BIX uses a fixed emission wavelength (430 nm) in the denominator.[21,33] The quality control criterion of rejecting samples with high noise eliminated samples with a broad range of β/α values (**Figure S7C**), including most β/α values >1 that were systematically greater than the retained data. Therefore, similar to HIX, applying the QC criterion decreases the distribution of β/α values in the retained dataset.

Both β/α and BIX were calculated and showed good agreement with each other (**Figure S8**). Data fall near the 1:1 line up to about 0.8. At higher values for each index, there was a systematic bias where BIX was greater than β/α. If a spectrum is blue-shifted, the β/α method uses the maximum value, which lies closer to the lower bound of the range (i.e., 420 nm). However, BIX uses a fixed wavelength in the denominator near the upper bound of the emission range for β/α, which increases BIX relative to β/α. Only β/α was used in further analysis.

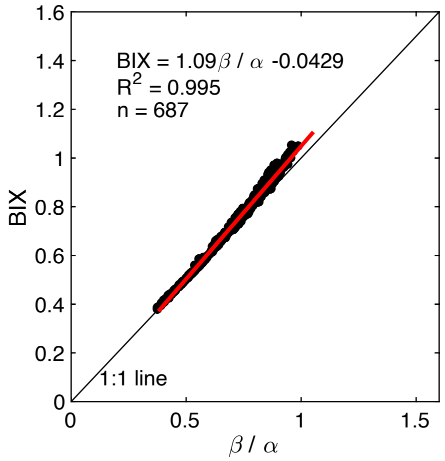

Figure S8. Comparison of BIX to β/α. Solid black line is 1:1 line. The red line shows a linear regression relating both metrics.

Fluorescence Index. The Cory and McKnight (2010)[22] approach was used to calculate the fluorescence index (FI). When FI was originally proposed in McKnight et al. (2001),[23] FI calculated the ratio of intensities at emission wavelengths of 450 nm to 500 nm at an excitation of 370 nm. However, in 2010, the emission wavelengths were revised to 470 and 520 nm due to a red-shift in the emission spectra when instrument-specific correction factors were applied. Since all spectra in this study applied correction factors, the Cory and McKnight (2010) definition is used. Unlike HIX and β/α, the quality control criterion rejected samples uniformly across the range of observed FI values (**Figure S7A**).

Maximum Emission Wavelength. Wavelengths of maximum fluorescence intensity indicate how red- or blue-shifted the fluorescence spectrum is at a given excitation wavelength. Although less commonly reported than fluorescence surrogates like FI, the fluorescence emission maximum wavelength has been shown to correlate with FI.[23,27] Therefore, the wavelength of maximum emission was calculated at 254, 310, and 370 nm excitation, because emission spectra at these

excitation wavelengths were used to calculate HIX, β/α, and FI, respectively. The same respective quality control criteria were used to reject noisy samples. Note that the emission intensity vector at 254 nm excitation was interpolated data, as described for HIX. The supplemental MATLAB code constrains the search region between the 1st and 2nd Rayleigh scatter lines based on the excitation wavelength. The analytical uncertainty in reporting peak emission wavelength depends on the instrument bandpass. The standard operating procedure for the data included in this meta-analysis used a bandpass of 5 nm, and previous work assessed a 95% confidence interval of $\pm$6 nm using this procedure and instrument.[27]

Specific Peak Intensity. Fluorescence peak intensities could be calculated using two approaches in the conventionally defined A, B, C, and T regions.[27] The first approach would define fixed wavelengths in the center of regions commonly assigned to each peak.[30,34] Alternatively, an algorithmic approach would find the maximum intensity within a prescribed wavelength region.[27] The fixed wavelength approach was used to calculate the carbon-normalized specific peak intensities, as defined in **Table S2**. The supplemental MATLAB code also includes a function (Eval_Peaks.m) that could calculate peak intensities using an algorithmic approach[27]. Although algorithmic peak intensities are not presented in this study, others could use that approach to calculate specific peak intensities and the excitation-emission wavelengths at which the fluorescence maxima occur. **Figure S7D-G** shows that the QC criteria systematically rejected samples with low specific peak intensities.

S 2 Overview of Dataset

Most of the meta-analysis dataset was curated from published articles and publicly available theses. **Table S4** show that the studies used a broad range of starting materials (e.g., whole-water vs. isolate, freshwater aquatic environments vs. wastewater effluent vs. stormwaters) and some applied a range of treatment processes (e.g., coagulation, ozonation, adsorption). Each sample was categorized based on pre-defined sample attributes (Table 1 of main manuscript). **Table S4** reports the total number of samples from each study with the associated sample attributes, which are assigned individually to each sample. For example, a study with ozonation would have some samples designated as Treated with a paired control sample designated as Natural.

Natural samples are defined as DOM collected or isolated from a natural environment without any additional physicochemical transformations beyond standard isolation methods (e.g., XAD fractionation, ultrafiltration, or reverse osmosis). Samples designated as Treated were subjected to a perturbation through the addition of a chemical (e.g., oxidant, reductant) or physicochemical separation process (e.g., coagulation, activated carbon, or biofiltration). Samples with the designation Treated have control samples that are untreated and categorized accordingly for data analysis. Origin describes if the DOM sample came from a natural aquatic system (e.g., surface water, groundwater, estuary, or marine environment) compared to anthropogenic systems (e.g., wastewater effluent or stormwater runoff). Whole-Water versus Isolate samples signal that an isolation process was conducted between sample collection and the analysis of optical properties. For example, any study that uses material from the International Humic Substances Society (IHSS) has the designation of Isolate. Although collected from a natural aquatic ecosystem, reference material is isolated using either reverse osmosis or XAD resin. Finally, Size Fractionation denotes if ultrafiltration was used, and if so, the nominal size fractions based on membrane cutoffs. Size Fractionated samples were not designated as Treated unless another treatment process was applied (e.g., coagulation, granular active carbon (GAC), or borohydride reduction) after fractionation.

Table S4. Index of studies contributing absorbance spectra and EEMs to meta-analysis.

Source	n	Natural vs. Treated	Origin	Whole-Water vs. Isolate	Treatment	Size Fractionation
Mostafa et al. (2014)[35]	8	Natural	Aquatic<br>Wastewater Effluent	Isolate<br>Whole-Water	–	<1k<br>1k-10k<br>>10k
McKay et al. (2018)[36]	10	Natural	Aquatic	Isolate	–	–
Shimabuku et al. (2017)[37]	23	Treated	Aquatic	Whole-Water	GAC	–
Shimabuku et al. (2017)[37]	48	Treated	Aquatic	Isolate	GAC	<1k<br>>1k
Shimabuku et al. (2017)[37]	59	Treated	Aquatic<br>Wastewater Effluent	Whole-Water	GAC<br>Chlorination	–
Shimabuku (unpublished)	29	Treated	Aquatic<br>Wastewater Effluent	Whole-Water	Biological Filtration	–
Li and McKay (2021)[38]	5	Natural	Aquatic	Whole-Water<br>Isolate	Borohydride	–
Korak et al. (2015)[39]	98	Treated	Aquatic	Whole-Water	Coagulation	–
Korak et al. (2014)[40]	18	Treated	Aquatic	Isolate	Coagulation	–
McKay et al. (2018)[41]	9	Natural	Aquatic	Isolate	–	<1k<br><5k<br>>5k
McKay et al. (2017)[42]	21	Natural	Aquatic<br>Estuary	Whole-Water	–	–
McKay et al. (2016)[43]	17	Treated	Aquatic<br>Wastewater Effluent	Whole-Water<br>Isolate	Borohydride<br>Coagulation	<5k<br>>5k
Önnby et al. (2018)[44]<br>Leresche et al. (2019)[45]	71	Treated	Aquatic<br>Wastewater Effluent	Whole-Water<br>Isolate	Ozonation	–
Shimabuku et al. (2014)[46]	131	Treated	Aquatic	Whole-Water<br>Isolate	Coagulation + PAC<br>Coagulation<br>GAC	<1k<br>>1k
Hohner (2011)[47]	28	Natural	Aquatic	Whole-Water	–	–
Shimabuku (2017)[48]	14	Natural	Stormwater	Whole-Water	–	–
Korak et al. (2015)[49]	5	Treated	Aquatic	Whole-Water	Ozonation	–
Korak (unpublished)	31	Natural	Aquatic<br>Wastewater Effluent	Whole-Water	–	–
Kaplan (2020)[50]	119	Treated	Aquatic	Whole-Water	Ozonation, Chlorine dioxide<br>Pre-oxidation+Coagulation	–
Allen et al. (2023)[51]	18	Natural	Aquatic	Isolate	pH adjusted	–

Table S5. Index of studies contributing absorbance spectra and EEMs to meta-analysis.

Source	Origin	n	Description
Mostafa et al. (2014)[35]	Aquatic<br>Wastewater Effluent	8	Suwannee River natural organic matter and secondary treated municipal wastewater effluent (Boulder, Colorado) were size fractionated into distinct molecular weight fractions: <1 kDa, 1-10 kDa, >10 kDa, and bulk
McKay et al. (2018)[36]	Aquatic	10	Thirteen humic substance and RO/ED isolates were studied, including samples from the IHSS and the USGS (provided by George Aiken). Soil samples included ESHA and PPHA (and PPFA), but excluded Leonardite HA (a low-grade coal). Aquatic samples spanned a range of environments (as judged by 0.7 $\leq SUVA_{254} \leq$ 4.9). IHSS samples were either NOM, FA, or HA, while USGS samples were HPOA.
Shimabuku et al. (2017) [37]	Aquatic	23	Of the three waters in this study, this subset is a drinking water sampled after alum coagulation (Betasso Water Treatment Plant, Boulder, Colorado). This facility treats a non-wastewater-impacted source water.
Shimabuku et al. (2017) [37]	Aquatic	48	Of the three waters in this study, this subset was collected from Big Elk Meadows (Colorado), a mountain reservoir with high DOC (~15 mg_C/L), low alkalinity, and low conductivity. The Big Elk Meadows sample was fractionated using a 1 kDa membrane and tangential flow ultrafiltration.
Shimabuku et al. (2017)[37]	Aquatic<br>Wastewater Effluent	59	Of the three waters in this study, this subset is a tertiary treated wastewater collected from Boulder, Colorado prior to UV disinfection. The water was treated by GAC and includes one chlorinated sample.
Shimabuku (unpublished)	Aquatic<br>Wastewater Effluent	29	Three whole water samples were treated with biological filtration. There were two surface waters and one tertiary treated wastewater effluent. The surface waters included Barker Reservoir (Nederland, Colorado) and Wonderland Lake (Boulder, Colorado). Both can be considered as a forested watershed, with minimal urban impact. The wastewater sample is from Boulder, Colorado.
Li and McKay (2021)[38]	Aquatic	7	Humic substance and RO/ED isolates from the IHSS, including both aquatic and soil environments.
Korak et al. (2015)[39]	Aquatic	98	22 surface waters with a wide range of $SUVA_{254}$ (1.0 to 4.0 L mg_C^{-1} m^{-1}). Source waters included several in Colorado, and fewer in diverse locations such as Lake Mead, Nevada; Danville, Kentucky; Red River, North Dakota; Red Lake, Minnesota; and Lake Erie, Ohio.
Korak et al. (2014)[40]	Aquatic	18	IHSS isolates from the Suwannee River, including the fulvic acid, humic acid, and RO/ED isolate.
McKay et al. (2018)[41]	Aquatic	9	Sample set overlaps with McKay et al. (2018)[36]
McKay et al. (2017)[42]	Aquatic<br>Estuary	21	Whole water samples from the Florida Everglades Long Term Ecological Research Network. Samples range from freshwater to marine, as determined by spatiotemporal factors described in the publication.
McKay et al. (2016)[43]	Aquatic<br>Wastewater Effluent	17	Suwannee River fulvic acid was ultrafiltered with a 5 kDa cutoff into <5 kDa, >5 kDa, and bulk fractions and subsequently reduced with sodium borohydride. The treated wastewater effluent was coagulated with varying doses of alum (0-120 mg/L)
Önnby et al. (2018)[44]<br>Leresche et al. (2019)[45]	Aquatic<br>Wastewater Effluent	71	Suwannee River fulvic acid and Pony Lake fulvic acid were reacted with ozone at doses ranging from 0 to 1 mmol O_3 mmol C^{-1} at three pH values: 3, 7, or 9. In addition, whole water samples included a surface water from the San Juan River (near Farmington, NM) and a second treated wastewater effluent (Boulder, Colorado) reacted with ozone (same dosages as isolates) at ambient pH. t-Butanol was spiked prior to ozonation.
Shimabuku et al. (2014)[46]	Aquatic	131	Surface waters were collected from Colorado and New York, with a range of $SUVA_{254}$ from 1.4 to 2.5 L mg_C^{-1} m^{-1}. One of the samples (Big Elk Meadows, Colorado) was ultrafiltered into <1 kDa and >1 kDa size fractions.
Hohner (2011)[47]	Aquatic	28	Surface water were collected from reservoirs across Colorado, USA, with $SUVA_{254}$ ranging from 1.1 to 3.3 L mg_C^{-1} m^{-1}.

Table S5 continued

**Source**	**Origin**	**n**	**Description**
Shimabuku (2017)[48]	Stormwater	14	Six different stormwater samples were collected, including two parking lot outfalls, one mixed outfall (parking lot, building roofs, roads, and lawns), one effluent from a retention basin that received mixed runoff, one industrially-influenced runoff, and one neighborhood (suburban) runoff. Stormwaters had $SUVA_{254}$ values ranging from 1.3 to 3.1 L mg_C^{-1} m^{-1}.
Korak et al. (2015)[49]	Aquatic	5	Colorado River water was sampled from Lake Mead in Las Vegas, Nevada. Three samples were oxidized with ozone.
Korak (unpublished)	Aquatic<br>Wastewater Effluent	31	Four samples were collected from the drinking and wastewater facilities in Boulder, Colorado. Wastewater samples include both primary and secondary treated effluents. Drinking water samples were collected after coagulation and after chlorination. Drinking water and wastewater samples were blended in varying proportions to simulate various *de facto* reuse scenarios. Waters are classified as aquatic or wastewater by the majority contribution.
Kaplan (2020)[50]	Aquatic	119	Surface water samples were collected from a reservoir in the Denver, Colorado metropolitan area and a canal supplying water during a spring flushing event. The canal samples were pre-oxidized with permanganate followed by coagulation. The reservoir samples were pre-oxidized with permanganate, ozone, or chlorine dioxide prior to coagulation. Coagulation used alum, ferric chloride, or aluminum chlorohydrate.
Allen et al. (2023)[51]	Aquatic	18	Several humic substance and RO/ED isolates (from IHSS) were subjected to pH titrations ($2 \leq pH \leq 12$). Samples included aquatic samples (SRFA, SRHA, MRNOM) and soil isolates (ESHA, PPHA, PPFA).

Table S6 through **Table S17** document the summary statistics based on the categorical variables in Table 1 of the main text. Both extrinsic and intrinsic optical surrogates are reported. Extrinsic optical surrogates depend on DOC concentration, such as absorbance and fluorescence intensity. Intrinsic optical surrogates measure differences in composition, and summary data report absorbance- and fluorescence-based surrogates.

Differences in number of observations reported for each optical surrogate depend on either the scope of the original study or the impact of the quality control criteria. For example, the study that collected from an estuary only measured absorbance and DOC;[42] therefore, fluorescence surrogates are not reported. Many treatment-focused studies analyzed absorbance up to 600 nm, so the sample size for $S_{300\text{-}600}$ (n=722) is more than double the sample size of $S_{300\text{-}650}$ or $S_{300\text{-}700}$ (n=335). Additionally, DOC was not measured for the studies with coagulation combined with powdered activated carbon (PAC) or biological filtration. Therefore, carbon-normalized optical surrogates are not reported. The sample size for E4:E6 (n=10) is much smaller than E2:E3 (n=549), because either absorbance was not measured above 600 nm, or the absorbance was below the quality control threshold (0.005) at 665 nm.

Table S6. Summary statistics of extrinsic optical surrogates differentiating whole-waters from isolates

Subset	Statistic	UV_{254} (cm^{-1})	UV_{280} (cm^{-1})	UV_{320} (cm^{-1})	UV_{370} (cm^{-1})	Peak A (RU)	Peak B (RU)	Peak C (RU)	Peak T (RU)	OFI (RU nm^2)
All	n	729	725	693	539	698	693	695	693	693
	Min	0.0089	0.0058	0.0018	0.0011	0.0667	0.0057	0.0294	0.0252	164
	25%	0.0271	0.0188	0.0096	0.0060	0.285	0.062	0.121	0.081	3515
	Mean	0.0790	0.0574	0.0373	0.0204	0.760	0.196	0.448	0.234	12422
	Median	0.0581	0.0396	0.0191	0.0098	0.501	0.0935	0.229	0.120	6565
	75%	0.0939	0.0678	0.0379	0.0182	0.884	0.193	0.391	0.264	11845
	Max	0.582	0.436	0.856	0.395	19.0	3.12	13.0	5.16	356242
Whole Water	n	557	554	522	384	527	538	521	536	510
	Min	0.0089	0.0058	0.0018	0.0011	0.0667	0.0093	0.0294	0.0252	389
	25%	0.0231	0.0167	0.0077	0.0052	0.243	0.0693	0.104	0.0873	2866
	Mean	0.0679	0.0494	0.0258	0.0134	0.654	0.189	0.301	0.245	8893
	Median	0.0450	0.0331	0.0164	0.0085	0.433	0.101	0.204	0.127	5741
	75%	0.0868	0.0619	0.0323	0.0170	0.883	0.232	0.384	0.333	11215
	Max	0.512	0.361	0.237	0.110	4.14	2.98	1.85	4.38	65809
Isolate	n	172	171	171	155	171	155	174	157	183
	Min	0.0098	0.0058	0.0072	0.0020	0.0754	0.0057	0.0492	0.0265	164
	25%	0.0639	0.0401	0.0175	0.0080	0.481	0.044	0.204	0.058	5725
	Mean	0.1150	0.0836	0.0723	0.0378	1.09	0.217	0.889	0.195	22256
	Median	0.0827	0.0582	0.0286	0.0131	0.655	0.0657	0.296	0.0907	8205
	75%	0.1096	0.0834	0.0532	0.0282	0.882	0.103	0.452	0.155	12489
	Max	0.582	0.436	0.856	0.395	19.0	3.12	13.0	5.16	356242

Table S7. Summary statistics of intrinsic absorbance surrogates differentiating whole-waters from isolates

Subset	Statistic	$SUVA_{254}$	$SUVA_{280}$	$SUVA_{320}$	$SUVA_{370}$	E2:E3	E4:E6	$S_{300-700}$	$S_{300-650}$	$S_{300-600}$	$S_{275-295}$	$S_{350-400}$	S_R
		($L\ mg_c^{-1}\ m^{-1}$)				(–)	(–)	(nm^{-1})	(nm^{-1})	(nm^{-1})	(nm^{-1})	(nm^{-1})	(–)
All	n	503	501	488	394	549	10	335	335	722	728	419	419
	Min	0.651	0.360	0.115	0.035	3.23	1.50	0.00319	0.00335	0.00260	-0.00002	0.0092	0.610
	25%	1.27	0.88	0.366	0.159	5.40	1.67	0.0160	0.0160	0.0164	0.0146	0.0166	0.785
	Mean	2.04	1.46	0.730	0.348	7.43	3.79	0.0190	0.0190	0.0197	0.0189	0.0184	0.929
	Median	1.76	1.21	0.538	0.243	6.82	1.78	0.0175	0.0175	0.0185	0.0183	0.0184	0.919
	75%	2.54	1.82	0.901	0.439	8.48	6.51	0.0215	0.0215	0.0219	0.0229	0.0198	1.02
	Max	7.55	6.12	4.10	2.26	25.9	8.43	0.0445	0.0445	0.0473	0.0357	0.0347	1.83
Whole Water	n	362	362	356	274	400	10	235	235	543	552	286	284
	Min	0.651	0.360	0.115	0.046	3.30	1.50	0.00319	0.00335	0.00374	-0.00002	0.0092	0.619
	25%	1.24	0.847	0.339	0.151	5.56	1.67	0.0165	0.0165	0.0168	0.0152	0.0165	0.871
	Mean	1.79	1.28	0.605	0.282	7.38	3.79	0.0193	0.0193	0.0200	0.0192	0.0179	0.982
	Median	1.55	1.09	0.494	0.201	6.83	1.78	0.0177	0.0177	0.0186	0.0185	0.0183	0.950
	75%	2.12	1.51	0.724	0.349	8.60	6.51	0.0219	0.0219	0.0220	0.0233	0.0195	1.06
	Max	6.83	5.26	3.51	1.72	21.9	8.43	0.0445	0.0445	0.0473	0.0326	0.0258	1.83
Isolate	n	141	139	132	120	149	0	100	100	179	176	133	135
	Min	0.669	0.401	0.143	0.035	3.23		0.01268	0.01268	0.00260	0.00997	0.0134	0.610
	25%	1.74	1.15	0.506	0.250	4.72		0.0154	0.0154	0.0154	0.0131	0.0168	0.726
	Mean	2.66	1.93	1.07	0.498	7.57		0.0182	0.0182	0.0189	0.0179	0.0196	0.815
	Median	2.58	1.80	0.88	0.358	6.72		0.0165	0.0165	0.0180	0.0166	0.0188	0.783
	75%	3.51	2.60	1.56	0.744	8.36		0.0194	0.0194	0.0211	0.0209	0.0205	0.857
	Max	7.55	6.12	4.10	2.26	25.9		0.0315	0.0315	0.0422	0.0357	0.0347	1.35

Table S8. Summary statistics of intrinsic fluorescence surrogates differentiating whole-waters from isolates

Subset	Statistic	FI (-)	$\lambda_{em,max}$ at λ_{ex} = 370 nm (nm)	HIX (-)	β/α (-)	$\lambda_{em,max}$ at λ_{ex} = 310 nm (nm)	Sp. Peak A (RU L mg_c^{-1})	Sp. Peak B (RU L mg_c^{-1})	Sp. Peak C (RU L mg_c^{-1})	Sp. Peak T (RU L mg_c^{-1})
All	n	698	680	700	700	698	477	457	472	466
	Min	1.18	426	0.486	0.36	394	0.0406	0.0019	0.0185	0.0071
	25%	1.42	456	0.724	0.60	414	0.121	0.0226	0.0507	0.0322
	Mean	1.59	460	0.788	0.71	421	0.186	0.0496	0.0829	0.0652
	Median	1.59	460	0.793	0.74	418	0.171	0.0363	0.0728	0.0496
	75%	1.68	466	0.864	0.81	426	0.219	0.0592	0.100	0.0765
	Max	2.18	474	0.960	1.01	460	0.817	0.301	0.290	0.465
Whole Water	n	525	518	535	525	524	339	340	335	337
	Min	1.31	426	0.486	0.41	394	0.0406	0.0049	0.0185	0.0097
	25%	1.50	454	0.710	0.69	414	0.117	0.0295	0.0469	0.0417
	Mean	1.64	459	0.760	0.75	418	0.178	0.0585	0.0778	0.0728
	Median	1.62	460	0.756	0.76	416	0.157	0.0438	0.0687	0.0579
	75%	1.73	462	0.825	0.84	424	0.198	0.0667	0.0863	0.0890
	Max	2.18	474	0.923	1.01	460	0.817	0.301	0.275	0.416
Isolate	n	173	162	165	175	174	138	117	137	129
	Min	1.18	444	0.599	0.36	396	0.0454	0.0019	0.0193	0.0071
	25%	1.34	460	0.847	0.45	418	0.138	0.0114	0.0619	0.0194
	Mean	1.44	465	0.880	0.59	429	0.206	0.0239	0.0955	0.0455
	Median	1.43	466	0.886	0.57	428	0.208	0.0201	0.0845	0.0287
	75%	1.54	472	0.928	0.70	438	0.254	0.0254	0.128	0.0430
	Max	1.79	474	0.960	1.00	460	0.629	0.255	0.290	0.465

Table S9. Summary statistics of extrinsic optical surrogates differentiating treated and natural samples

Subset	Statistic	UV_{254} (cm^{-1})	UV_{280} (cm^{-1})	UV_{320} (cm^{-1})	UV_{370} (cm^{-1})	Peak A (RU)	Peak B (RU)	Peak C (RU)	Peak T (RU)	OFI (RU nm^2)
All	n	729	725	693	539	698	693	695	693	693
	Min	0.0089	0.0058	0.0018	0.0011	0.0667	0.0057	0.0294	0.0252	164
	25%	0.0271	0.0188	0.0096	0.0060	0.285	0.0621	0.121	0.0813	3515
	Mean	0.0790	0.0574	0.0373	0.0204	0.760	0.196	0.448	0.234	12422
	Median	0.0581	0.0396	0.0191	0.0098	0.501	0.0935	0.229	0.120	6565
	75%	0.0939	0.0678	0.0379	0.0182	0.884	0.193	0.391	0.264	11845
	Max	0.582	0.436	0.856	0.395	19.0	3.12	13.0	5.16	356242
Natural	n	201	200	201	182	178	174	180	181	182
	Min	0.0100	0.0068	0.0020	0.0013	0.1036	0.0093	0.0316	0.0425	1041
	25%	0.0595	0.0406	0.0173	0.0081	0.502	0.0846	0.217	0.110	6400
	Mean	0.1156	0.0848	0.0451	0.0216	0.999	0.238	0.442	0.329	13458
	Median	0.0941	0.0680	0.0332	0.0155	0.855	0.128	0.363	0.179	11687
	75%	0.1365	0.1037	0.0559	0.0261	1.175	0.282	0.576	0.379	15830
	Max	0.512	0.361	0.237	0.110	4.14	2.98	1.83	4.38	65809
Treated	n	528	525	492	357	520	519	515	512	511
	Min	0.0089	0.0058	0.0018	0.0011	0.0667	0.0057	0.0294	0.0252	164
	25%	0.0230	0.0166	0.0078	0.0041	0.239	0.0564	0.103	0.0753	2756
	Mean	0.0651	0.0470	0.0341	0.0198	0.678	0.181	0.450	0.200	12053
	Median	0.0454	0.0329	0.0158	0.0079	0.432	0.087	0.197	0.110	5444
	75%	0.0791	0.0566	0.0283	0.0146	0.701	0.139	0.319	0.183	8957
	Max	0.582	0.436	0.856	0.395	19.0	3.12	13.0	5.16	356242

Table S10. Summary statistics of intrinsic absorbance surrogates differentiating treated and natural samples

Subset	Statistic	$SUVA_{254}$	$SUVA_{280}$	$SUVA_{320}$	$SUVA_{370}$	E2:E3	E4:E6	$S_{300\text{-}700}$	$S_{300\text{-}650}$	$S_{300\text{-}600}$	$S_{275\text{-}295}$	$S_{350\text{-}400}$	S_R
			(L mg_c^{-1} m^{-1})			(–)	(–)	(nm^{-1})	(nm^{-1})	(nm^{-1})	(nm^{-1})	(nm^{-1})	(–)
All	n	503	501	488	394	549	10	335	335	722	728	419	419
	Min	0.651	0.360	0.115	0.035	3.23	1.50	0.00319	0.00335	0.00260	-0.00002	0.0092	0.610
	25%	1.27	0.88	0.366	0.159	5.40	1.67	0.0160	0.0160	0.0164	0.0146	0.0166	0.785
	Mean	2.04	1.46	0.730	0.348	7.43	3.79	0.0190	0.0190	0.0197	0.0189	0.0184	0.929
	Median	1.76	1.21	0.538	0.243	6.82	1.78	0.0175	0.0175	0.0185	0.0183	0.0184	0.919
	75%	2.54	1.82	0.901	0.439	8.48	6.51	0.0215	0.0215	0.0219	0.0229	0.0198	1.02
	Max	7.55	6.12	4.10	2.26	25.9	8.43	0.0445	0.0445	0.0473	0.0357	0.0347	1.83
Natural	n	166	166	167	151	184	8	101	101	202	203	152	152
	Min	0.651	0.401	0.157	0.077	3.23	1.50	0.00319	0.00335	0.00374	0.00631	0.0092	0.610
	25%	1.71	1.19	0.519	0.219	4.98	1.65	0.0151	0.0151	0.0155	0.0135	0.0154	0.798
	Mean	2.46	1.81	0.949	0.450	6.69	2.97	0.0168	0.0168	0.0178	0.0174	0.0171	0.977
	Median	2.23	1.63	0.804	0.373	5.99	1.73	0.0160	0.0160	0.0171	0.0169	0.0174	0.961
	75%	3.18	2.35	1.17	0.576	8.41	3.53	0.0188	0.0188	0.0199	0.0209	0.0190	1.10
	Max	6.83	5.26	3.51	1.77	13.8	8.43	0.0445	0.0445	0.0445	0.0326	0.0254	1.63
Treated	n	337	335	321	243	365	2	234	234	520	525	267	267
	Min	0.678	0.360	0.115	0.035	3.43	6.51	0.00868	0.00902	0.00260	-0.00002	0.0113	0.612
	25%	1.23	0.84	0.331	0.142	5.69	6.51	0.0169	0.0169	0.0169	0.0149	0.0174	0.782
	Mean	1.83	1.29	0.616	0.285	7.80	7.08	0.0199	0.0199	0.0205	0.0194	0.0192	0.901
	Median	1.53	1.06	0.469	0.195	7.03	7.08	0.0181	0.0181	0.0191	0.0187	0.0188	0.898
	75%	2.18	1.54	0.762	0.319	8.54	7.64	0.0230	0.0230	0.0233	0.0239	0.0201	0.97
	Max	7.55	6.12	4.10	2.26	25.9	7.64	0.0394	0.0394	0.0473	0.0357	0.0347	1.83

Table S11. Summary statistics of intrinsic fluorescence surrogates differentiating treated and natural samples

Subset	Statistic	FI (-)	$\lambda_{em,max}$ at λ_{ex} = 370 nm (nm)	HIX (-)	β/α (-)	$\lambda_{em,max}$ at λ_{ex} = 310 nm (nm)	Sp. Peak A (RU L mg_c^{-1})	Sp. Peak B (RU L mg_c^{-1})	Sp. Peak C (RU L mg_c^{-1})	Sp. Peak T (RU L mg_c^{-1})
All	n	698	680	700	700	698	477	457	472	466
	Min	1.18	426	0.486	0.36	394	0.0406	0.0019	0.0185	0.0071
	25%	1.42	456	0.724	0.60	414	0.121	0.0226	0.0507	0.0322
	Mean	1.59	460	0.788	0.71	421	0.186	0.0496	0.0829	0.0652
	Median	1.59	460	0.793	0.74	418	0.171	0.0363	0.0728	0.0496
	75%	1.68	466	0.864	0.81	426	0.219	0.0592	0.100	0.0765
	Max	2.18	474	0.960	1.01	460	0.817	0.301	0.290	0.465
Natural	n	177	172	179	172	173	143	139	145	146
	Min	1.18	430	0.504	0.36	396	0.0569	0.0025	0.0246	0.0077
	25%	1.41	455	0.742	0.59	414	0.167	0.0260	0.0700	0.0363
	Mean	1.57	460	0.804	0.71	422	0.237	0.0539	0.104	0.0735
	Median	1.50	460	0.809	0.74	420	0.209	0.0399	0.0954	0.0513
	75%	1.67	466	0.872	0.81	426	0.277	0.0668	0.135	0.0945
	Max	2.16	474	0.957	0.99	460	0.817	0.301	0.275	0.416
Treated	n	521	508	521	528	525	334	318	327	320
	Min	1.23	426	0.486	0.37	394	0.0406	0.0019	0.0185	0.0071
	25%	1.44	456	0.718	0.60	414	0.110	0.0213	0.0435	0.0305
	Mean	1.60	460	0.783	0.71	421	0.165	0.0477	0.0734	0.0615
	Median	1.61	460	0.778	0.73	418	0.154	0.0343	0.0677	0.0487
	75%	1.68	466	0.860	0.82	426	0.197	0.0549	0.0818	0.0714
	Max	2.18	474	0.960	1.01	460	0.629	0.301	0.290	0.465

Table S12. Summary statistics of extrinsic optical surrogates differentiating aquatic sources

Subset	Statistic	UV_{254} (cm^{-1})	UV_{280} (cm^{-1})	UV_{320} (cm^{-1})	UV_{370} (cm^{-1})	Peak A (RU)	Peak B (RU)	Peak C (RU)	Peak T (RU)	OFI (RU nm^2)
All	n	729	725	693	539	698	693	695	693	693
	Min	0.0089	0.0058	0.0018	0.0011	0.0667	0.0057	0.0294	0.0252	164
	25%	0.0271	0.0188	0.0096	0.0060	0.285	0.0621	0.121	0.0813	3515
	Mean	0.0790	0.0574	0.0373	0.0204	0.760	0.196	0.448	0.234	12422
	Median	0.0581	0.0396	0.0191	0.0098	0.501	0.0935	0.229	0.120	6565
	75%	0.0939	0.0678	0.0379	0.0182	0.884	0.193	0.391	0.264	11845
	Max	0.582	0.436	0.856	0.395	19.0	3.12	13.0	5.16	356242
Aquatic	n	603	599	569	453	591	577	588	577	611
	Min	0.0089	0.0058	0.0018	0.0011	0.0667	0.0057	0.0294	0.0252	164
	25%	0.0257	0.0182	0.0086	0.0054	0.273	0.0586	0.113	0.0776	3326
	Mean	0.0792	0.0573	0.0382	0.0209	0.704	0.183	0.440	0.207	11861
	Median	0.0598	0.0401	0.0189	0.0101	0.475	0.0880	0.213	0.110	6086
	75%	0.0949	0.0686	0.0378	0.0180	0.772	0.128	0.343	0.183	10027
	Max	0.582	0.436	0.856	0.395	19.0	3.12	13.0	5.16	356242
Wastewater Effluent	n	99	99	95	62	93	102	93	102	68
	Min	0.0091	0.0069	0.0055	0.0012	0.0968	0.0093	0.0371	0.0252	495
	25%	0.0292	0.0220	0.0123	0.0067	0.415	0.0962	0.211	0.141	6837
	Mean	0.0568	0.0435	0.0233	0.0123	0.908	0.243	0.468	0.351	15029
	Median	0.0439	0.0347	0.0190	0.0090	0.697	0.198	0.357	0.262	11377
	75%	0.0761	0.0544	0.0293	0.0168	1.242	0.393	0.636	0.558	25894
	Max	0.280	0.224	0.110	0.058	3.72	0.734	1.69	1.12	65809
Estuary	n	13	13	15	13	0	0	0	0	0
	Min	0.0512	0.0322	0.0119	0.0070					
	25%	0.158	0.105	0.0501	0.0234					
	Mean	0.266	0.191	0.110	0.0543					
	Median	0.227	0.159	0.120	0.0576					
	75%	0.399	0.296	0.161	0.0733					
	Max	0.488	0.361	0.237	0.110					
Stormwater	n	14	14	14	11	14	14	14	14	14
	Min	0.0280	0.0177	0.0078	0.0060	0.889	0.147	0.305	0.151	12042
	25%	0.0424	0.0302	0.0149	0.0064	1.14	0.204	0.425	0.392	14152
	Mean	0.0544	0.0367	0.0178	0.0080	2.16	0.370	0.647	0.483	24214
	Median	0.0561	0.0364	0.0185	0.0080	2.08	0.326	0.615	0.497	22471
	75%	0.0684	0.0427	0.0210	0.0093	3.13	0.439	0.853	0.594	33419
	Max	0.0780	0.0512	0.0252	0.0110	4.09	0.998	1.10	0.741	44515

Table S13. Summary statistics of intrinsic absorbance surrogates differentiating aquatic sources

Subset	Statistic	$SUVA_{254}$	$SUVA_{280}$	$SUVA_{320}$	$SUVA_{370}$	E2:E3	E4:E6	$S_{300-700}$	$S_{300-650}$	$S_{300-600}$	$S_{275-295}$	$S_{350-400}$	S_R
		($L\ mg_c^{-1}\ m^{-1}$)				(–)	(–)	(nm^{-1})	(nm^{-1})	(nm^{-1})	(nm^{-1})	(nm^{-1})	(–)
All	n	503	501	488	394	549	10	335	335	722	728	419	419
	Min	0.651	0.360	0.115	0.035	3.23	1.50	0.00319	0.00335	0.00260	-0.00002	0.0092	0.610
	25%	1.27	0.88	0.366	0.159	5.40	1.67	0.0160	0.0160	0.0164	0.0146	0.0166	0.785
	Mean	2.04	1.46	0.730	0.348	7.43	3.79	0.0190	0.0190	0.0197	0.0189	0.0184	0.929
	Median	1.76	1.21	0.538	0.243	6.82	1.78	0.0175	0.0175	0.0185	0.0183	0.0184	0.919
	75%	2.54	1.82	0.901	0.439	8.48	6.51	0.0215	0.0215	0.0219	0.0229	0.0198	1.02
	Max	7.55	6.12	4.10	2.26	25.9	8.43	0.0445	0.0445	0.0473	0.0357	0.0347	1.83
Aquatic	n	441	439	424	345	459	4	260	260	599	606	369	371
	Min	0.651	0.360	0.115	0.035	3.23	1.67	0.00847	0.00876	0.00260	0.00631	0.0092	0.610
	25%	1.27	0.88	0.362	0.157	5.43	1.72	0.0161	0.0161	0.0164	0.0156	0.0171	0.784
	Mean	2.04	1.45	0.724	0.344	7.54	2.63	0.0189	0.0189	0.0197	0.0197	0.0187	0.926
	Median	1.73	1.19	0.526	0.232	6.95	1.78	0.0175	0.0175	0.0183	0.0192	0.0185	0.916
	75%	2.58	1.82	0.878	0.418	8.57	3.53	0.0210	0.0210	0.0219	0.0237	0.0200	1.02
	Max	7.55	6.12	4.10	2.26	25.9	5.27	0.0394	0.0394	0.0473	0.0357	0.0347	1.83
Wastewater Effluent	n	34	34	35	25	66	6	60	60	94	93	35	33
	Min	0.678	0.410	0.126	0.046	3.30	1.50	0.00319	0.00335	0.00374	-0.00002	0.0097	0.641
	25%	1.77	1.39	0.657	0.327	4.83	1.62	0.0149	0.0149	0.0163	0.0120	0.0129	0.770
	Mean	2.06	1.60	0.870	0.437	6.76	4.56	0.0194	0.0194	0.0198	0.0135	0.0155	0.913
	Median	1.87	1.46	0.818	0.422	5.76	4.10	0.0186	0.0186	0.0191	0.0127	0.0155	0.844
	75%	2.11	1.69	0.939	0.472	7.99	7.64	0.0230	0.0230	0.0233	0.0150	0.0173	0.992
	Max	6.50	5.26	3.25	1.72	21.4	8.43	0.0445	0.0445	0.0445	0.0326	0.0251	1.56
Estuary	n	15	15	15	13	13	0	15	15	15	15	12	12
	Min	0.679	0.426	0.157	0.093	5.43		0.01575	0.01575	0.01575	0.01684	0.0158	0.974
	25%	1.56	1.00	0.388	0.228	5.56		0.0161	0.0161	0.0161	0.0175	0.0163	1.007
	Mean	2.73	1.96	0.940	0.459	7.06		0.0185	0.0185	0.0185	0.0207	0.0180	1.057
	Median	2.89	2.12	1.08	0.501	6.02		0.0169	0.0169	0.0169	0.0186	0.0171	1.053
	75%	3.41	2.51	1.27	0.587	8.55		0.0200	0.0200	0.0200	0.0234	0.0206	1.10
	Max	6.83	4.96	2.45	1.09	10.5		0.0241	0.0241	0.0241	0.0301	0.0216	1.19
Stormwaters	n	13	13	14	11	11	0	0	0	14	14	3	3
	Min	0.656	0.438	0.157	0.119	5.61				0.01702	0.01474	0.0173	0.849
	25%	0.90	0.65	0.298	0.128	6.45				0.0185	0.0163	0.0176	0.872
	Mean	1.13	0.76	0.356	0.160	7.41				0.0198	0.0176	0.0186	0.910
	Median	1.18	0.77	0.371	0.159	7.46				0.0200	0.0171	0.0184	0.940
	75%	1.37	0.89	0.420	0.187	8.35				0.0207	0.0183	0.0197	0.941
	Max	1.56	1.02	0.504	0.220	8.83				0.0226	0.0234	0.0201	0.941

Table S14. Summary statistics of intrinsic fluorescence surrogates differentiating aquatic sources

Subset	Statistic	FI (-)	$\lambda_{em,max}$ at λ_{ex} = 370 nm (nm)	HIX (-)	β/α (-)	$\lambda_{em,max}$ at λ_{ex} = 310 nm (nm)	Sp. Peak A (RU L mg_c^{-1})	Sp. Peak B (RU L mg_c^{-1})	Sp. Peak C (RU L mg_c^{-1})	Sp. Peak T (RU L mg_c^{-1})
All	n	698	698	700	700	694	477	457	472	466
	Min	1.18	394	0.486	0.359	402	0.0406	0.0019	0.0185	0.0071
	25%	1.42	414	0.724	0.596	414	0.121	0.0226	0.0507	0.0322
	Mean	1.59	421	0.788	0.709	422	0.186	0.0496	0.0829	0.0652
	Median	1.59	418	0.793	0.736	420	0.171	0.0363	0.0728	0.0496
	75%	1.68	426	0.864	0.814	428	0.219	0.0592	0.100	0.0765
	Max	2.18	460	0.960	1.01	468	0.817	0.301	0.290	0.465
Aquatic	n	594	590	587	591	588	431	409	426	418
	Min	1.18	394	0.486	0.359	402	0.0406	0.0019	0.0185	0.0071
	25%	1.40	416	0.722	0.571	414	0.118	0.0218	0.0475	0.0308
	Mean	1.54	422	0.793	0.690	423	0.173	0.0449	0.0774	0.0578
	Median	1.56	420	0.803	0.720	420	0.162	0.0340	0.0701	0.0478
	75%	1.64	428	0.872	0.785	428	0.209	0.0520	0.0914	0.0666
	Max	2.04	460	0.960	1.01	464	0.629	0.272	0.290	0.465
Wastewater Effluent	n	90	94	99	95	93	32	34	32	34
	Min	1.40	400	0.608	0.413	404	0.0552	0.0086	0.0238	0.0139
	25%	1.83	408	0.728	0.774	414	0.173	0.0641	0.0864	0.1074
	Mean	1.91	415	0.760	0.816	421	0.251	0.0964	0.136	0.144
	Median	1.95	414	0.754	0.828	422	0.242	0.0879	0.127	0.140
	75%	2.04	418	0.793	0.867	426	0.318	0.128	0.166	0.173
	Max	2.18	460	0.888	0.967	468	0.493	0.301	0.275	0.397
Estuary	n	0	0	0	0	0	0	0	0	0
	Min									
	25%									
	Mean									
	Median									
	75%									
	Max									
Stormwaters	n	14	14	14	14	14	14	14	14	14
	Min	1.37	442	0.599	0.555	396	0.178	0.0295	0.0609	0.0302
	25%	1.48	448	0.677	0.750	410	0.227	0.0408	0.0850	0.0784
	Mean	1.53	455	0.778	0.778	413	0.432	0.0740	0.129	0.0967
	Median	1.49	453	0.813	0.784	414	0.416	0.0652	0.123	0.0994
	75%	1.59	462	0.829	0.797	416	0.627	0.0878	0.171	0.119
	Max	1.79	472	0.884	0.958	424	0.817	0.200	0.220	0.148

Table S15. Summary statistics of extrinsic optical surrogates differentiating treatment perturbation

Subset	Statistic	UV_{254} (cm^{-1})	UV_{280} (cm^{-1})	UV_{320} (cm^{-1})	UV_{370} (cm^{-1})	Peak A (RU)	Peak B (RU)	Peak C (RU)	Peak T (RU)	OFI (RU nm^2)
All	n	729	725	693	539	698	693	695	693	693
	Min	0.0089	0.0058	0.0018	0.0011	0.0667	0.0057	0.0294	0.0252	164
	25%	0.0271	0.0188	0.0096	0.0060	0.285	0.0621	0.121	0.0813	3515
	Mean	0.0790	0.0574	0.0373	0.0204	0.760	0.196	0.448	0.234	12422
	Median	0.0581	0.0396	0.0191	0.0098	0.501	0.0935	0.229	0.120	6565
	75%	0.0939	0.0678	0.0379	0.0182	0.884	0.193	0.391	0.264	11845
	Max	0.582	0.436	0.856	0.395	19.0	3.12	13.0	5.16	356242
All Treated	n	528	525	492	357	520	519	515	512	511
	Min	0.0089	0.0058	0.0018	0.0011	0.0667	0.0057	0.0294	0.0252	164
	25%	0.0230	0.0166	0.0078	0.0041	0.239	0.0564	0.103	0.0753	2756
	Mean	0.0651	0.0470	0.0341	0.0198	0.678	0.181	0.450	0.200	12053
	Median	0.0454	0.0329	0.0158	0.0079	0.432	0.0870	0.197	0.110	5444
	75%	0.0791	0.0566	0.0283	0.0146	0.701	0.139	0.319	0.183	8957
	Max	0.582	0.436	0.856	0.395	19.0	3.12	13.0	5.16	356242
Ozonation	n	67	67	66	61	66	45	65	48	67
	Min	0.0130	0.0072	0.0032	0.0012	0.105	0.0057	0.0364	0.0297	389
	25%	0.0527	0.0322	0.0123	0.0035	0.383	0.0225	0.150	0.0464	4120
	Mean	0.0687	0.0462	0.0225	0.0093	0.540	0.0762	0.238	0.109	6841
	Median	0.0714	0.0454	0.0193	0.0085	0.561	0.0452	0.245	0.0603	7000
	75%	0.0879	0.0638	0.0332	0.0146	0.676	0.0764	0.320	0.131	8599
	Max	0.109	0.0814	0.0463	0.0231	1.05	0.495	0.469	0.556	15213
Coagulation	n	108	108	103	51	108	108	108	107	108
	Min	0.0138	0.0078	0.0050	0.0053	0.115	0.0106	0.0481	0.0446	1614
	25%	0.0261	0.0184	0.0075	0.0072	0.228	0.0655	0.0985	0.0868	3157
	Mean	0.0772	0.0559	0.0290	0.0217	0.654	0.122	0.311	0.170	8997
	Median	0.0424	0.0293	0.0134	0.0152	0.459	0.0882	0.229	0.117	6623
	75%	0.0963	0.0661	0.0318	0.0328	0.792	0.149	0.369	0.221	10963
	Max	0.289	0.218	0.123	0.0590	3.30	0.375	1.48	0.507	42669
Pre-Oxidation then Coagulation	n	109	107	106	96	110	110	105	110	110
	Min	0.0098	0.0058	0.0018	0.0011	0.0704	0.0241	0.0294	0.0308	941
	25%	0.0193	0.0132	0.0053	0.0024	0.194	0.0739	0.0693	0.0873	2471
	Mean	0.0264	0.0193	0.0088	0.0039	0.298	0.204	0.129	0.162	4115
	Median	0.0220	0.0157	0.0073	0.0031	0.278	0.0933	0.113	0.111	3690
	75%	0.0296	0.0226	0.0104	0.0042	0.342	0.479	0.168	0.336	5191
	Max	0.0801	0.0616	0.0334	0.0153	0.763	0.617	0.337	0.393	10604

Table S15 continued

Subset	Statistic	UV_{254} (cm^{-1})	UV_{280} (cm^{-1})	UV_{320} (cm^{-1})	UV_{370} (cm^{-1})	Peak A (RU)	Peak B (RU)	Peak C (RU)	Peak T (RU)	OFI (RU nm^2)
Coagulation and PAC	n	40	40	21	6	38	37	38	39	40
	Min	0.0089	0.0068	0.0054	0.0128	0.0667	0.0222	0.0306	0.0309	854
	25%	0.0132	0.0097	0.0066	0.0147	0.105	0.0421	0.0511	0.0506	1399
	Mean	0.0280	0.0207	0.0158	0.0154	0.265	0.125	0.136	0.150	3912
	Median	0.0175	0.0129	0.0076	0.0150	0.134	0.0621	0.0646	0.0643	1869
	75%	0.0231	0.0168	0.0338	0.0173	0.214	0.0852	0.0987	0.0812	2556
	Max	0.0955	0.0747	0.0424	0.0174	1.08	0.523	0.575	0.708	17631
Biological Filtration	n	29	29	29	19	29	28	29	28	29
	Min	0.0221	0.0142	0.0060	0.0061	0.406	0.0180	0.162	0.0702	5057
	25%	0.0267	0.0193	0.0104	0.0070	0.530	0.0848	0.221	0.127	6609
	Mean	0.0416	0.0313	0.0169	0.0090	1.17	0.262	0.629	0.420	17451
	Median	0.0432	0.0329	0.0182	0.0072	1.10	0.247	0.540	0.371	15760
	75%	0.0453	0.0342	0.0191	0.0084	1.82	0.442	1.056	0.731	28312
	Max	0.0867	0.0722	0.0472	0.0236	2.22	0.608	1.34	0.832	34938
Borohydride Reduction	n	6	6	7	7	7	7	7	7	7
	Min	0.0662	0.0454	0.0223	0.0074	1.50	0.113	0.664	0.164	16626
	25%	0.0791	0.0563	0.0309	0.0114	1.63	0.156	0.721	0.213	17723
	Mean	0.2106	0.1539	0.0969	0.0442	5.19	0.566	3.211	0.960	73647
	Median	0.1190	0.0873	0.0467	0.0193	2.801	0.346	1.846	0.760	48005
	75%	0.4295	0.3003	0.1747	0.0897	9.79	0.940	6.158	1.568	137449
	Max	0.451	0.347	0.220	0.104	11.9	1.28	7.92	2.21	172016
GAC	n	159	158	144	101	151	168	148	171	134
	Min	0.0090	0.0058	0.0051	0.0050	0.0739	0.0097	0.0371	0.0252	164
	25%	0.0458	0.0321	0.0139	0.0077	0.360	0.0562	0.162	0.0815	4063
	Mean	0.0648	0.0463	0.0231	0.0110	0.535	0.107	0.238	0.185	5977
	Median	0.0663	0.0476	0.0215	0.0104	0.471	0.0840	0.213	0.114	5718
	75%	0.0900	0.0623	0.0289	0.0132	0.732	0.109	0.291	0.173	7622
	Max	0.134	0.113	0.0554	0.0205	1.44	0.580	0.718	1.59	19087

Table S16. Summary statistics of intrinsic absorbance surrogates differentiating treatment perturbation

Subset	Statistic	$SUVA_{254}$	$SUVA_{280}$	$SUVA_{320}$	$SUVA_{370}$	E2:E3	E4:E6	$S_{300-700}$	$S_{300-650}$	$S_{300-600}$	$S_{275-295}$	$S_{350-400}$	S_R
		($L\ mg_c^{-1}\ m^{-1}$)				(–)	(–)	(nm^{-1})	(nm^{-1})	(nm^{-1})	(nm^{-1})	(nm^{-1})	(–)
All	n	503	501	488	394	549	10	335	335	722	728	419	419
	Min	0.651	0.360	0.115	0.035	3.23	1.50	0.00319	0.00335	0.00260	-0.00002	0.0092	0.610
	25%	1.27	0.88	0.366	0.159	5.40	1.67	0.0160	0.0160	0.0164	0.0146	0.0166	0.785
	Mean	2.04	1.46	0.730	0.348	7.43	3.79	0.0190	0.0190	0.0197	0.0189	0.0184	0.929
	Median	1.76	1.21	0.538	0.243	6.82	1.78	0.0175	0.0175	0.0185	0.0183	0.0184	0.919
	75%	2.54	1.82	0.901	0.439	8.48	6.51	0.0215	0.0215	0.0219	0.0229	0.0198	1.02
	Max	7.55	6.12	4.10	2.26	25.9	8.43	0.0445	0.0445	0.0473	0.0357	0.0347	1.83
All Treated	n	337	335	321	243	365	2	234	234	520	525	267	267
	Min	0.678	0.360	0.115	0.035	3.43	6.51	0.00868	0.00902	0.00260	-0.00002	0.0113	0.612
	25%	1.23	0.84	0.331	0.142	5.69	6.51	0.0169	0.0169	0.0169	0.0149	0.0174	0.782
	Mean	1.83	1.29	0.616	0.285	7.80	7.08	0.0199	0.0199	0.0205	0.0194	0.0192	0.901
	Median	1.53	1.06	0.469	0.195	7.03	7.08	0.0181	0.0181	0.0191	0.0187	0.0188	0.898
	75%	2.18	1.54	0.762	0.319	8.54	7.64	0.0230	0.0230	0.0233	0.0239	0.0201	0.972
	Max	7.55	6.12	4.10	2.26	25.9	7.64	0.0394	0.0394	0.0473	0.0357	0.0347	1.83
Ozonation	n	66	66	66	59	59	2	66	66	66	67	53	55
	Min	0.678	0.394	0.126	0.035	4.06	6.51	0.01208	0.01211	0.01219	0.01223	0.0122	0.612
	25%	1.18	0.71	0.244	0.082	5.50	6.51	0.0171	0.0171	0.0171	0.0142	0.0184	0.715
	Mean	1.92	1.32	0.655	0.289	10.5	7.08	0.0219	0.0219	0.0219	0.0195	0.0223	0.790
	Median	1.68	1.08	0.468	0.203	7.83	7.08	0.0212	0.0212	0.0212	0.0180	0.0213	0.784
	75%	2.25	1.70	0.916	0.450	14.6	7.64	0.0258	0.0258	0.0258	0.0237	0.0260	0.841
	Max	3.94	2.99	1.85	0.923	25.9	7.64	0.0394	0.0394	0.0394	0.0319	0.0347	1.17
Coagulation	n	98	98	94	50	58	0	7	7	105	108	28	28
	Min	0.719	0.478	0.157	0.092	3.43		0.01491	0.01491	0.01278	0.01044	0.0131	0.721
	25%	1.44	0.97	0.337	0.215	5.18		0.0163	0.0163	0.0168	0.0152	0.0162	0.773
	Mean	2.24	1.63	0.843	0.521	6.71		0.0172	0.0172	0.0218	0.0202	0.0175	0.888
	Median	1.78	1.32	0.628	0.382	6.43		0.0172	0.0172	0.0197	0.0200	0.0183	0.814
	75%	2.76	1.96	0.983	0.734	8.10		0.0183	0.0183	0.0241	0.0251	0.0192	0.906
	Max	7.55	6.12	4.10	2.26	11.2		0.0192	0.0192	0.0473	0.0313	0.0212	1.83
Pre-Oxidation then Coagulation	n	109	109	108	98	98	0	110	110	110	110	83	83
	Min	0.698	0.360	0.115	0.069	5.07		0.01280	0.01286	0.01295	0.01508	0.0155	0.860
	25%	1.15	0.79	0.313	0.134	6.67		0.0171	0.0171	0.0171	0.0179	0.0182	0.932
	Mean	1.22	0.87	0.384	0.165	7.86		0.0191	0.0191	0.0191	0.0210	0.0190	1.02
	Median	1.23	0.87	0.392	0.172	7.25		0.0175	0.0175	0.0175	0.0186	0.0190	0.957
	75%	1.35	1.00	0.465	0.192	8.10		0.0209	0.0209	0.0209	0.0244	0.0200	0.990
	Max	1.79	1.37	0.745	0.34	14.8		0.0317	0.0317	0.0317	0.0293	0.0223	1.52

Table S16. continued

Subset	Statistic	$SUVA_{254}$	$SUVA_{280}$	$SUVA_{320}$	$SUVA_{370}$	E2:E3	E4:E6	$S_{300\text{-}700}$	$S_{300\text{-}650}$	$S_{300\text{-}600}$	$S_{275\text{-}295}$	$S_{350\text{-}400}$	S_R
		($L\ mg_c^{-1}\ m^{-1}$)				(–)	(–)	(nm^{-1})	(nm^{-1})	(nm^{-1})	(nm^{-1})	(nm^{-1})	(–)
Coagulation and PAC	n	0	0	0	0	6	0	0	0	36	39	6	6
	Min					5.48				0.01375	0.01051	0.0156	0.619
	25%					5.49				0.0175	0.0193	0.0159	0.634
	Mean					5.71				0.0197	0.0214	0.0165	0.647
	Median					5.67				0.0191	0.0218	0.0164	0.646
	75%					5.85				0.0221	0.0252	0.0170	0.659
	Max					6.10				0.0254	0.0312	0.0174	0.676
Biological Filtration	n	0	0	0	0	19	0	0	0	29	29	4	4
	Min					3.61				0.01251	0.00883	0.0113	0.711
	25%					4.97				0.0168	0.0122	0.0136	0.715
	Mean					5.49				0.0183	0.0155	0.0148	0.732
	Median					5.56				0.0181	0.0144	0.0159	0.718
	75%					6.01				0.0194	0.0176	0.0161	0.750
	Max					6.48				0.0234	0.0252	0.0164	0.782
Borohydride Reduction	n	3	3	3	3	7	0	7	7	7	7	6	6
	Min	2.374	1.629	0.799	0.266	4.22		0.01376	0.01376	0.01374	0.01512	0.0141	0.770
	25%	2.50	1.73	0.857	0.294	5.95		0.0169	0.0169	0.0169	0.0158	0.0175	0.776
	Mean	2.74	1.93	0.967	0.347	6.62		0.0178	0.0178	0.0178	0.0172	0.0193	0.901
	Median	2.88	2.04	1.029	0.377	6.92		0.0187	0.0187	0.0187	0.0176	0.0193	0.909
	75%	2.95	2.10	1.062	0.394	7.66		0.0191	0.0191	0.0191	0.0176	0.0226	0.970
	Max	2.98	2.12	1.07	0.399	8.00		0.0193	0.0193	0.0193	0.0197	0.0229	1.07
GAC	n	60	58	49	32	108	0	25	25	148	146	68	66
	Min	0.749	0.479	0.287	0.153	4.75		0.00868	0.00902	0.00260	-0.00002	0.0157	0.640
	25%	1.79	1.16	0.480	0.254	5.72		0.0205	0.0205	0.0172	0.0149	0.0174	0.850
	Mean	2.10	1.43	0.624	0.276	7.71		0.0231	0.0231	0.0213	0.0188	0.0185	0.925
	Median	2.19	1.48	0.657	0.291	7.87		0.0219	0.0219	0.0202	0.0184	0.0185	0.917
	75%	2.59	1.78	0.778	0.308	8.97		0.0237	0.0237	0.0241	0.0224	0.0197	1.03
	Max	2.97	2.09	0.898	0.347	12.9		0.0367	0.0367	0.0422	0.0357	0.0209	1.15

Table S17. Summary statistics of intrinsic fluorescence surrogates differentiating treatment perturbation

Subset	Statistic	FI (-)	$\lambda_{em,max}$ at λ_{ex} = 370 nm (nm)	HIX (-)	β/α (-)	$\lambda_{em,max}$ at λ_{ex} = 310 nm (nm)	Sp. Peak A (RU L mg_c^{-1})	Sp. Peak B (RU L mg_c^{-1})	Sp. Peak C (RU L mg_c^{-1})	Sp. Peak T (RU L mg_c^{-1})
All	n	698	680	700	700	698	477	457	472	466
	Min	1.18	426	0.486	0.359	394	0.0406	0.0019	0.0185	0.0071
	25%	1.42	456	0.724	0.596	414	0.121	0.0226	0.0507	0.0322
	Mean	1.59	460	0.788	0.709	421	0.186	0.0496	0.0829	0.0652
	Median	1.59	460	0.793	0.736	418	0.171	0.0363	0.0728	0.0496
	75%	1.68	466	0.864	0.814	426	0.219	0.0592	0.100	0.0765
	Max	2.18	474	0.960	1.01	460	0.817	0.301	0.290	0.465
All Treated	n	521	508	521	528	525	334	318	327	320
	Min	1.23	426	0.486	0.369	394	0.0406	0.0019	0.0185	0.0071
	25%	1.44	456	0.718	0.599	414	0.110	0.0213	0.0435	0.0305
	Mean	1.60	460	0.783	0.709	421	0.165	0.0477	0.0734	0.0615
	Median	1.61	460	0.778	0.735	418	0.154	0.0343	0.0677	0.0487
	75%	1.68	466	0.860	0.815	426	0.197	0.0549	0.0818	0.0714
	Max	2.18	474	0.960	1.01	460	0.629	0.301	0.290	0.465
Ozonation	n	66	63	62	64	63	65	46	63	49
	Min	1.23	440	0.671	0.369	408	0.0406	0.0019	0.0190	0.0071
	25%	1.46	460	0.838	0.462	422	0.079	0.0065	0.0315	0.0142
	Mean	1.55	461	0.868	0.644	431	0.148	0.0204	0.0670	0.0283
	Median	1.53	462	0.888	0.687	434	0.131	0.0098	0.0588	0.0187
	75%	1.64	464	0.928	0.766	440	0.210	0.0207	0.0978	0.0259
	Max	2.05	474	0.960	0.927	460	0.288	0.130	0.135	0.164
Coagulation	n	104	108	108	108	105	98	99	98	98
	Min	1.24	438	0.653	0.378	404	0.0662	0.0098	0.0275	0.0194
	25%	1.51	454	0.734	0.664	414	0.107	0.0246	0.0491	0.0383
	Mean	1.61	459	0.795	0.739	420	0.194	0.0429	0.0954	0.0603
	Median	1.62	460	0.772	0.769	418	0.157	0.0348	0.0823	0.0464
	75%	1.67	462	0.874	0.857	424	0.286	0.0436	0.135	0.0589
	Max	2.17	474	0.925	0.955	452	0.490	0.301	0.275	0.395
Pre-Oxidation then Coagulation	n	108	108	109	110	110	110	110	107	110
	Min	1.55	452	0.486	0.636	404	0.0494	0.0154	0.0185	0.0196
	25%	1.63	458	0.641	0.725	414	0.117	0.0441	0.0425	0.0533
	Mean	1.66	460	0.679	0.766	415	0.137	0.0762	0.0560	0.0683
	Median	1.65	460	0.688	0.757	416	0.139	0.0549	0.0634	0.0654
	75%	1.69	462	0.737	0.796	416	0.171	0.107	0.0693	0.0837
	Max	1.78	466	0.796	0.908	426	0.198	0.196	0.0805	0.122

Table S17 continued

Subset	Statistic	FI (-)	$\lambda_{em,max}$ at λ_{ex} = 370 nm (nm)	HIX (-)	β/α (-)	$\lambda_{em,max}$ at λ_{ex} = 310 nm (nm)	Sp. Peak A (RU L mg_c^{-1})	Sp. Peak B (RU L mg_c^{-1})	Sp. Peak C (RU L mg_c^{-1})	Sp. Peak T (RU L mg_c^{-1})
Coagulation and PAC	n	40	40	40	37	38	0	0	0	0
	Min	1.53	428	0.617	0.628	394				
	25%	1.59	455	0.693	0.730	412				
	Mean	1.65	457	0.716	0.786	415				
	Median	1.60	460	0.716	0.739	418				
	75%	1.71	462	0.739	0.887	422				
	Max	1.90	468	0.803	1.01	426				
Biological Filtration	n	29	29	29	29	29	0	0	0	0
	Min	1.36	438	0.688	0.510	406				
	25%	1.41	442	0.793	0.557	416				
	Mean	1.78	455	0.821	0.730	421				
	Median	1.65	454	0.805	0.791	416				
	75%	2.08	465	0.844	0.828	431				
	Max	2.14	474	0.923	0.859	438				
Borohydride Reduction	n	7	7	7	7	7	3	3	3	3
	Min	1.41	452	0.758	0.500	410	0.441	0.0314	0.193	0.0456
	25%	1.47	456	0.863	0.524	419	0.467	0.035	0.205	0.0510
	Mean	1.62	462	0.878	0.608	425	0.539	0.055	0.241	0.0729
	Median	1.62	464	0.896	0.539	426	0.546	0.046	0.241	0.0674
	75%	1.75	467	0.919	0.652	428	0.609	0.078	0.278	0.0962
	Max	1.96	470	0.926	0.931	440	0.629	0.089	0.290	0.106
GAC	n	148	137	156	154	154	57	59	55	59
	Min	1.31	426	0.599	0.481	396	0.0454	0.0095	0.0258	0.0192
	25%	1.34	456	0.766	0.554	416	0.138	0.019	0.059	0.0308
	Mean	1.53	462	0.811	0.669	421	0.166	0.024	0.067	0.0779
	Median	1.39	466	0.832	0.630	423	0.179	0.023	0.070	0.0373
	75%	1.63	472	0.864	0.760	428	0.205	0.027	0.079	0.0663
	Max	2.18	474	0.901	1.00	438	0.242	0.066	0.096	0.465

Potential of nitrate and iron to contribute to optical signals in Wastewater Effluent. $SUVA_{254}$ values greater than 5.5 L mg_C^{-1} m^{-1} are rare and often suffer from non-DOM constituents.[5,52] The small proportion (<3%) of samples with $SUVA_{254}$ >5.5 L mg_C^{-1} m^{-1} in the meta-analysis dataset either used SRHA,[40] were a >10 kDa size fraction,[35] or were from tannin-dominated estuarine samples in the Florida everglades.[42] Several of the whole-water samples in the meta-analysis dataset include treated wastewater effluent containing nitrate and iron, species known to interfere with optical measurements. Wastewater samples were identified as most likely to contain high levels of these species in the meta-analysis dataset based on the sample context. We address the potential impact of nitrate and iron on optical surrogates by considering the effect of varying species concentration on the mean values of $SUVA_{254}$ and E2:E3 for Wastewater Effluent samples in the meta-analysis dataset. Known molar absorption coefficients for nitrate (NO_3^-)[53] and iron (III)[52] were used for this analysis (as opposed to direct measurement), and it was assumed that the system obeyed the Beer-Lambert Law following (Equations S3-S5):

$$UV_{254,NO3}\ (cm^{-1}) = (15\ M^{-1}\ cm^{-1})[NO_3^-] \qquad \text{Equation S3}$$

$$UV_{254,Fe}\ (cm^{-1}) = (0.0653\ L\ mg_{Fe}^{-1}\ cm^{-1})\ [Fe(III)] + 0.002 \qquad \text{Equation S4}$$

$$UV_{254,Sample}\ (cm^{-1}) = UV_{254,mean\ wastewater} + UV_{254,NO3} + UV_{254,Fe} \qquad \text{Equation S5}$$

where $[NO_3^-]$ and [Fe(III)] are the concentrations of nitrate and iron (III) in mol/L and mg/L, respectively. **Figure S9** shows that nitrate is a minor contributor to the absorbance at 254 nm at concentrations less than 10 mg/L as NO_3^- and is thus unlikely to interfere with $SUVA_{254}$ and E2:E3. However, iron (III) concentrations of 1 mg/L exert a two-fold increase in absorbance at 254 nm and is thus highly likely to interfere with $SUVA_{254}$ and E2:E3 measurements. It is important to note that the molar absorption coefficients used for iron (III) were derived from laboratory-generated iron (III) solutions[52] that may differ from conditions in a wastewater treatment plant.

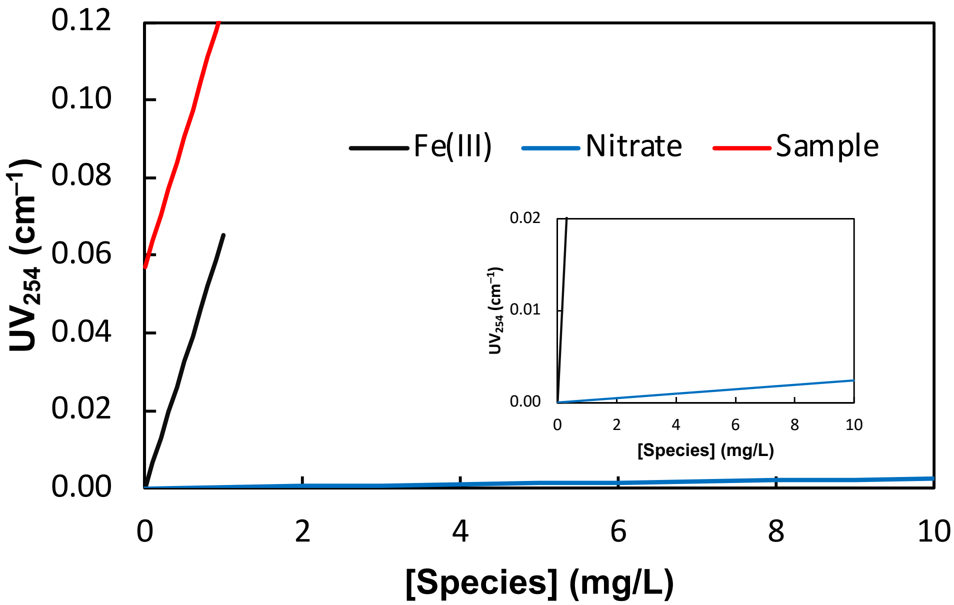

Figure S9. Calculation of absorbance at 254 nm (UV_{254}) as a function of iron (III) and nitrate concentrations. Inset shows the same calculations, but on a reduced y-axis scale.

Provided below are example calculations for assessing the impact of nitrate and iron on two absorbance-based surrogates, $SUVA_{254}$ and E2:E3, subjected to higher nitrate and iron concentrations. We focus on wastewater effluent samples since these have the most potential for spectral interference by non-DOM chromophores. Assuming a true (due to DOM) $SUVA_{254}$ of 2.0 L mg_C^{-1} cm^{-1} and a typical wastewater DOC concentration of 7 mg_C L^{-1}, this would lead to a UV_{254} of 0.14 cm^{-1}.

The absorbances at 254 nm due to two different concentrations of nitrate (ϵ_{254} = 15 $M^{-1}cm^{-1}$)[53] are

$$(30\ \text{mg-N L}^{-1})\times(1\ \text{mmol-N } 1/\ 14\ \text{mg-N})\times(1\ \text{mmol-N} / 1\text{mmol-NO}_3^-)\times (1\ \text{mol} / 1000\ \text{mmol})\times(15\ \text{L mol}^{-1}\ \text{cm}^{-1}) = \underline{0.0321\ \text{cm}^{-1}}$$

$$(10\ \text{mg-N L}^{-1})\times(1\ \text{mmol-N } 1/\ 14\ \text{mg-N})\times(1\ \text{mmol-N} / 1\text{mmol-NO}_3^-)\times (1\ \text{mol} / 1000\ \text{mmol})\times(15\ \text{L mol}^{-1}\ \text{cm}^{-1}) = \underline{0.0107\ \text{cm}^{-1}}$$

The absorbance due to 0.1 mg-Fe L^{-1} iron (ϵ_{254} = 0.0653 L mg-Fe cm^{-1})[52] is

$$(0.1\ \text{mg-Fe L}^{-1})\times(0.0653\ \text{L mg-Fe cm}^{-1}) = \underline{0.00653\ \text{cm}^{-1}}$$

The impact of these nitrate and iron concentrations on $SUVA_{254}$ are as follows:

$SUVA_{254,NO3(30)+DOC}$ = (0.14 + 0.0321) cm^{-1} / (7 mg_C L^{-1}) *100 = 2.5 L mg_C^{-1} m^{-1}
% Difference = 0.5/2*100 = 25%

$SUVA_{254,NO3(10)+DOC}$ = (0.14 + 0.0107) cm^{-1} / (7 mg_C L^{-1}) *100 = 2.2 L mg_C^{-1} m^{-1}
% Difference = 0.2/2*100 = 10%

$SUVA_{254,Fe+DOC}$ = (0.14 + 0.00653) cm^{-1} / (7 mg_C L^{-1}) *100 = 2.1 L mg_C^{-1} m^{-1}
% Difference = 0.1/2*100 = 5%

To determine the impact of nitrate and iron on E2:E3, an E2:E3 value of 7.5 was assumed, which falls within the range of values measured for wastewater effluent:

A_{370} = A_{254} / E2:E3 = 0.14 cm^{-1} / 7.5 = 0.01867 cm^{-1}

$E2{:}E3_{NO3(30)+DOC}$ = 0.1721 cm^{-1} / 0.01867 cm^{-1} = 9.22
% Difference = 2.72 / 7.5 *100 = 23%

$E2{:}E3_{NO3(10)+DOC}$ = 0.1507 cm^{-1} / 0.01867 cm^{-1} = 8.07
% Difference = 0.572 / 7.5 *100 = 7.6%

$E2{:}E3_{Fe+DOC}$ = 0.14653 cm^{-1} / 0.01867 cm^{-1} = 7.85
% Difference = 0.35 / 7.5 *100 = 4.7%

S 3 Comparison to Literature Data

Figure 1 of the main manuscript compares E2:E3, $SUVA_{254}$, and FI values from the meta-analysis dataset to four literature references: Kellerman et al. (2018),[54] McCabe and Arnold (2017),[55] McCabe and Arnold (2018)[56], and Berg et al. (2023).[57] We emphasized these datasets due to broad geographic diversity,[54] large sample sizes,[55,56] sample diversity,[57] and overlap in the optical parameters reported. For E2:E3, the median of the Natural subset (5.99, n=184) was similar to the three reference data sets (5.8–7.1). In the Natural subset, median $SUVA_{254}$ was lower and FI was higher than other references, which is likely due the higher proportion of whole-water, natural samples in the meta-analysis dataset. The Kellerman et al. (2018)[54] dataset reports hydrophobic organic acids (HPOA) isolates exclusively, and the HPOA fraction commonly has a higher $SUVA_{254}$ and lower FI than the whole-water.[23,58] The dataset in McCabe and Arnold (2017),[55] geographically focused in Minnesota, was weighted towards storm flow (n=186) compared to baseflow (n=29) and snowmelt (n=18) samples. The higher composition of storm flow samples may explain why the dataset has systematically higher $SUVA_{254}$ values compared to the Natural subset.[59,60] In addition to including mostly whole-water samples (n=160), our meta-analysis dataset also includes some wastewater effluents (n=25) where median values are lower for $SUVA_{254}$ (1.9 L mg_C^{-1} m^{-1}) and higher for FI (1.9). The dataset in Berg et al. (2023)[57] also includes wastewater samples and has a median value close to the distribution in the meta-analysis.

The meta-analysis data set was also compared to peer-reviewed datasets that included raw data for a similar breadth of optical surrogates. Before comparing sample statistics, consistent calculations methods were confirmed. HIX values were not be compared against two studies (Hodgkins et al. (2016) [61] and Kellerman et al. (2018) [54]), because HIX values greater than one suggest that the calculation method did not include the fluorescence emission between 435 and 480 nm in the denominator (see **Table S2**). There was also some ambiguity in the calculation method for β/α compared to BIX, as defined in **Table S2**. For example, the drEEM toolbox[62] calculates both β/α (reported as Freshness Index) and BIX and cites Parlanti et al. (2000)[17] and Huguet et al. (2009),[21] respectively. However, Parlanti et al. (2000) defines a range of excitation wavelengths, which differs from the two common calculation methods that use only excitation 310 nm. Kellerman et al. (2018) uses the header BIX but cites Parlanti et al. (2000) in the methods, leading to ambiguity. Based on the meta-analysis, however, the two methods differ by only about 6% (**Figure S8**). Lastly, Helms et al. (2008) reports Napierian absorbance coefficient ($A = a \times 2.303$, where *a* is the Napierian absorption coefficient), which is a factor of 2.303 higher than absorbance values reported in other studies. Thus, $SUVA_{254}$ values from Helms et al. (2008) plotted in **Figure S10** have been divided by 2.303 to facilitate comparison to other datasets.

The $SUVA_{254}$ values for the other literature references are higher than both the Natural and Treated subsets from the meta-analysis dataset. $SUVA_{254}$ values from Wologo et al. (2021)[63] are not included in **Figure S10**, because the mean value was greater than 5 L mg_C^{-1} m^{-1}. High values are often due to inorganic interferences, as acknowledged by Wologo et al. (2021). The higher reported $SUVA_{254}$ values for the other studies compared to the meta-analysis are likely due to the use of isolates[64] or the narrow context of arctic permafrost.[61,65]

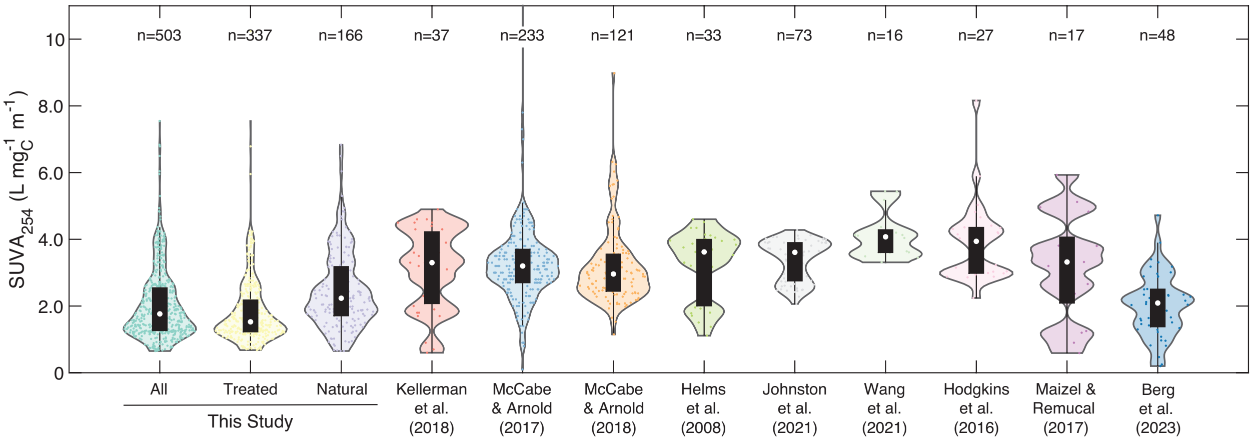

Figure S10. Comparison of $SUVA_{254}$ values between study subsets (Treated and Natural) and literature references.

In general, the literature datasets appear internally consistent with trends from the meta-analysis dataset. High $SUVA_{254}$ often co-occurs with low E2:E3, low β/α and high HIX.[56] Comparing datasets, other studies with higher median $SUVA_{254}$ values[54-57,61,64] also reported systematically lower median values for E2:E3 (**Figure S11**), $S_{275-295}$ (**Figure S12**), β/α (**Figure S15**) and higher median values for HIX (**Figure S14**). Despite higher median $SUVA_{254}$ values, several studies report similar or greater median values for FI (**Figure S16**). The dataset in Wang et al. (2021) does not match the internal consistency commonly observed in other datasets, reporting a higher median $SUVA_{254}$ than other studies (**Figure S10**), but systematically lower HIX (**Figure S14**), higher β/α (**Figure S15**), and higher FI (**Figure S16**).

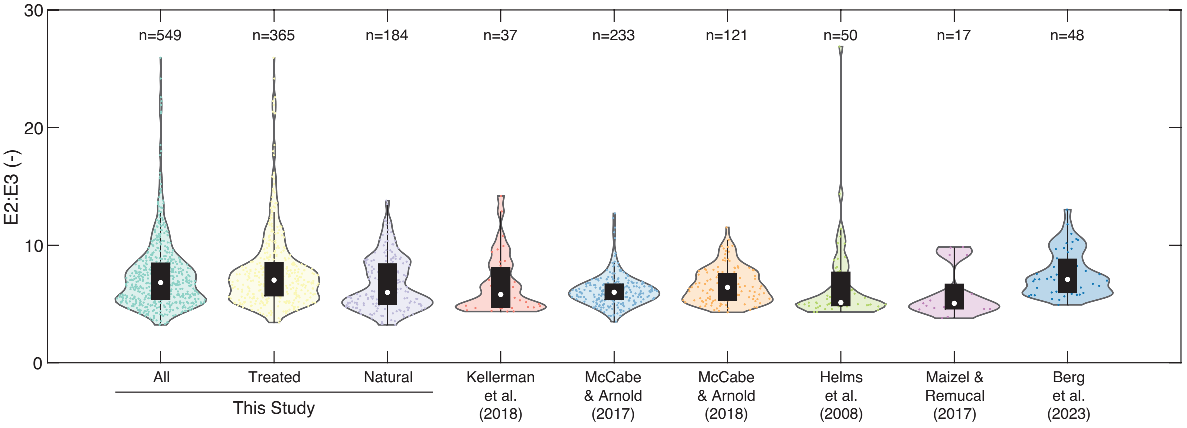

Figure S11. Comparison of E2:E3 values between study subsets (Treated and Natural) and literature references.

$S_{275-295}$ ranged between 0.01 and 0.03 nm^{-1} consistently across all studies. The range was smaller for Johnston et al. (2021)[65], Wang et al. (2021)[66], and Maizel et al. (2017)[64,67], but the sample size and context were also considerably smaller, leading to more homogeneity. Median values were systematically higher for the treated samples than natural, which aligns with the understanding of how coagulation, GAC and ozonation transform or selectively remove DOM with high molecular weight and aromaticity. Median values for the natural subset (0.017 nm^{-1}, n=203) were similar to

Helms et al. (2008)[8] (0.017 nm^{-1}, n=146) and higher than Kellerman et al. (2018)[54] (0.015, n=37) that only reported isolates.

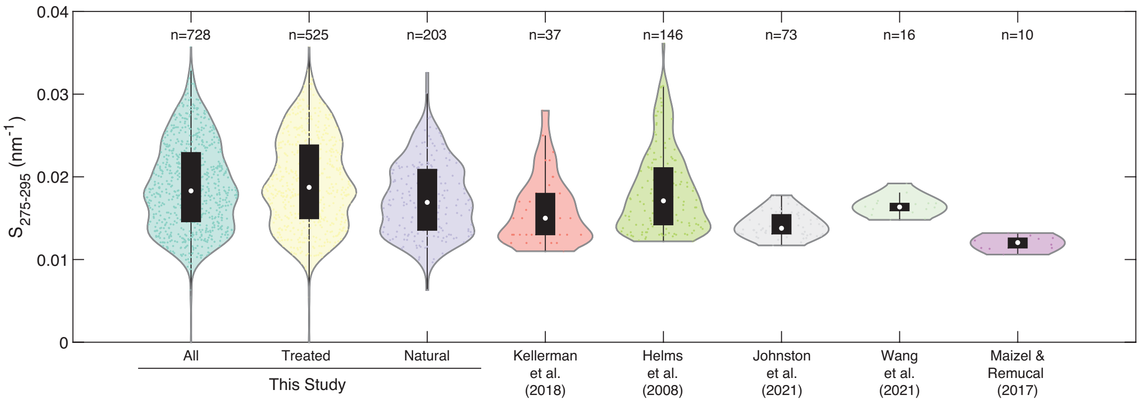

Figure S12. Comparison of $S_{275-295}$ values between study subsets (Treated and Natural) and literature references.

The median S_R values for treated samples is less than for Natural subset with a broader interquartile range (**Figure S13**). The median for Kellerman et al. (2018) (0.81, n=37) is lower than other datasets. The range and interquartile range of the Helms et al. (2008) dataset is larger than other studies, with 15% of the dataset having S_R values >1.5. The median S_R from Helms et al. (2008) is also greater than that of the meta-analysis dataset and Kellerman et al. (2018). All the S_R values for Wang et al. (2021) are greater than 1, which was not observed for any of the other studies nor the meta-analysis.

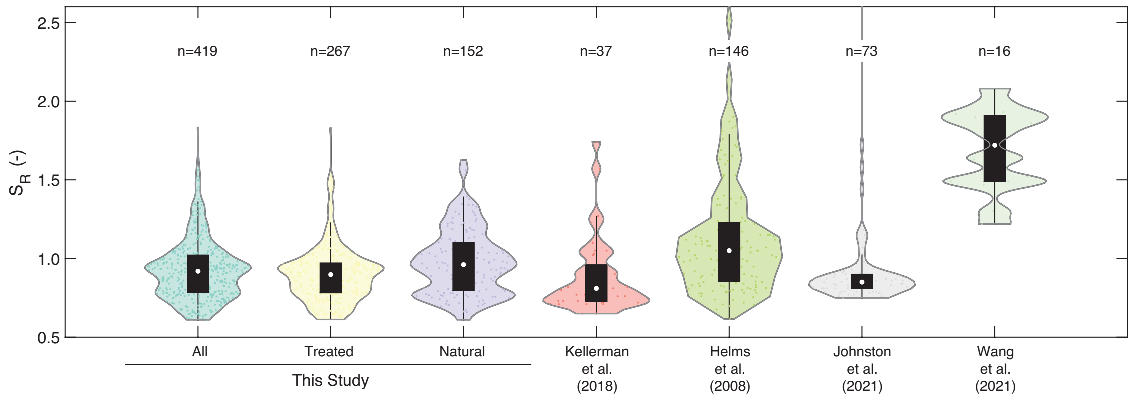

Figure S13 Comparison of S_R values between study subsets (Treated and Natural) and literature references. One sample in the Helms et al. (2008) dataset (S_R=12.8) is not shown.

HIX values in the meta-analysis were only compared to studies that reported HIX on a scale from 0 to 1 (**Table S2**). The distributions in the meta-analysis are similar with respect to interquartile range and overall range between the Treated and Natural subsets (**Figure S14**). The median value of the Natural subset (0.81, n=179) is higher than the Treated subset (0.78, n=521). The two McCabe and Arnold studies had higher median HIX values (0.88, n=354).[55,68] Higher HIX values in McCabe and Arnold (2017)[55] co-occurred with higher $SUVA_{254}$, which is consistent with

sample set dominated by stormflow. [59,60] Median HIX values (0.51, n=16) were systematically lower in the Wang et al. (2021) dataset.[66]

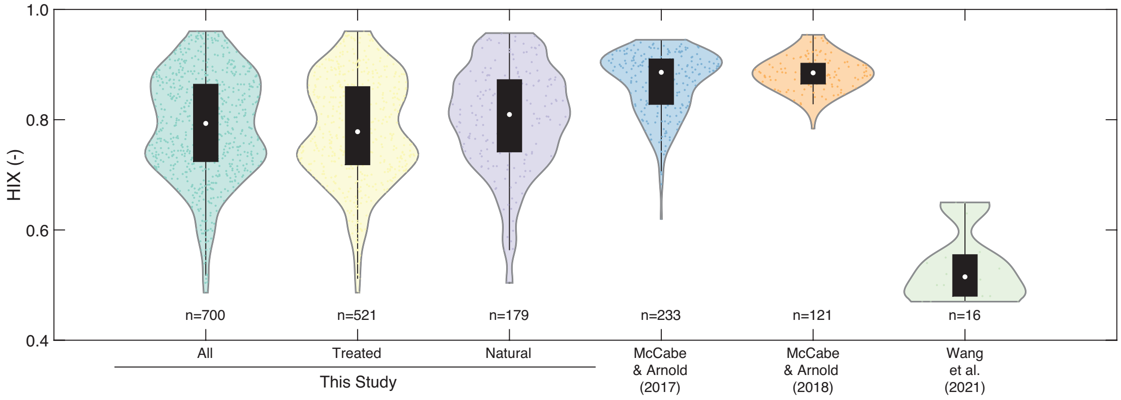

Figure S14 Comparison of HIX values between study subsets (Treated and Natural) and literature references.

The overall range of β/α values in the meta-analysis largely encompasses the range reported in other studies, except for Hodgkins et al. (2016)[61], which is lower (**Figure S15**). The interquartile range and medians value are similar across the Treated and Natural subsets in the meta-analysis. The width of the interquartile range is also similar to Kellerman et al. (2018),[54] but the median is lower in the Kellerman study (0.56), which aligns with a scope limited to isolates. The median values are similar across both McCabe and Arnold[55,68] (0.65-0.67) and Wologo studies (0.62).[63]

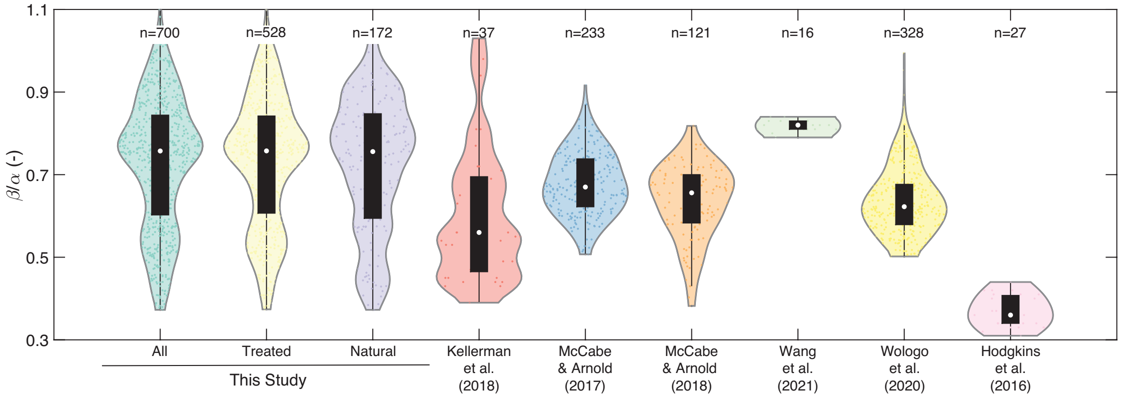

Figure S15 Comparison of β/α values between study subsets (Treated and Natural) and literature references.

The range of FI values in the meta-analysis dataset was larger than other studies (**Figure S16**), even though the central tendency (medians) were similar. The distribution of the whole data set largely reflects the distribution of the Treated subset due to many samples in this study having some treatment perturbation. There is a bimodal distribution with FI values around 1.6 and a smaller fraction around 1.4. A tail extends to samples above 2.0, which largely correspond to samples of wastewater origin (n=90) and/or biologically filtered samples (n=29). The Natural

subset had a lower median (1.5) than Treated subset. Kellerman et al. (2018) focused solely on isolates and has the lowest median FI of the literature datasets,[54] the rest of which included whole water samples. Berg et al. (2023)[57] also has a subset with high FI values (>1.9) associated with wastewater samples, which fills an underrepresented sample type in other literature datasets.

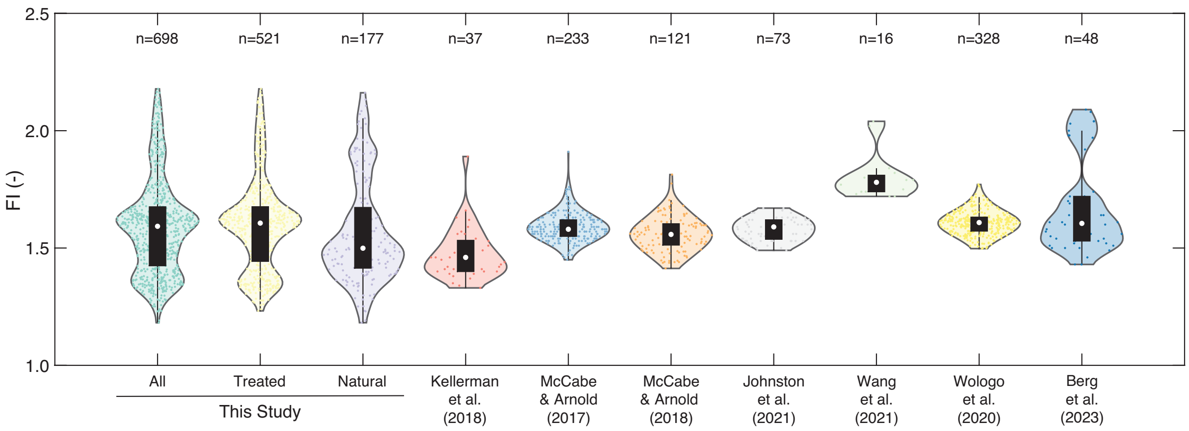

Figure S16 Comparison of FI values between study subsets (Treated and Natural) and literature references.

S 4 Correlation Analysis

S4.1 Optical surrogate correlations within the primary dataset

Correlations between optical surrogates retained after quality control screening were evaluated using the *corr* function in MATLAB. For each correlation, the Spearman correlation coefficient (ρ_S) and the p-value of the Spearman correlation coefficient (p_S) were calculated. **Figure S17** to **Figure S20** show heatmaps of ρ_S for all optical surrogates for a variety of sample types and treatments. To compare our dataset to existing literature,[69] heatmaps of the coefficient of determination (R^2) values were also generated (**Figure S21** to **Figure S24**), which evaluate a linear correlation as opposed to a monotonic one (like ρ_S). **Figure S25** to **Figure S33** display scatter plots for the meta-analysis dataset that are supplementary to the scatter plots in the main text. In these plots, ρ_S values for subsets of data are shown in the lollipop plots in the right column. To address the diverging correlations amongst subsets of data we evaluated all possible optical surrogate correlations for each data subset.

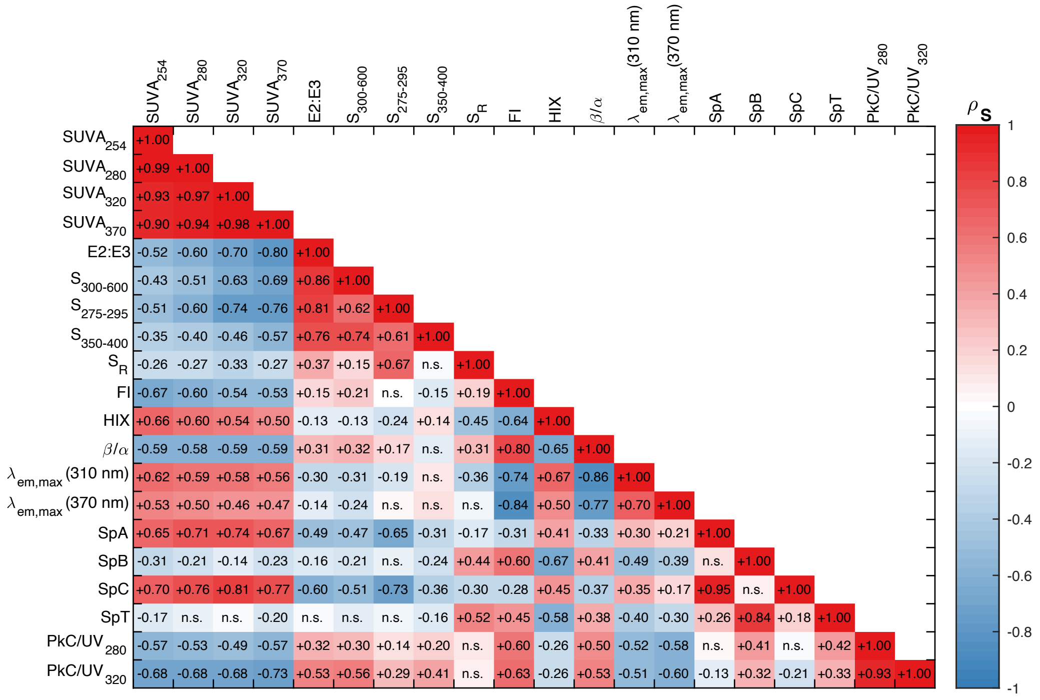

Figure S17. Heatmap of Spearman rho values (ρ_S) for all samples in dataset. ρ_S values are printed only if the corresponding p_S was less than or equal to 0.01. If p_S>0.01, the color corresponding to the ρ_S value of the sample is still included, but n.s. (not significant) is printed.

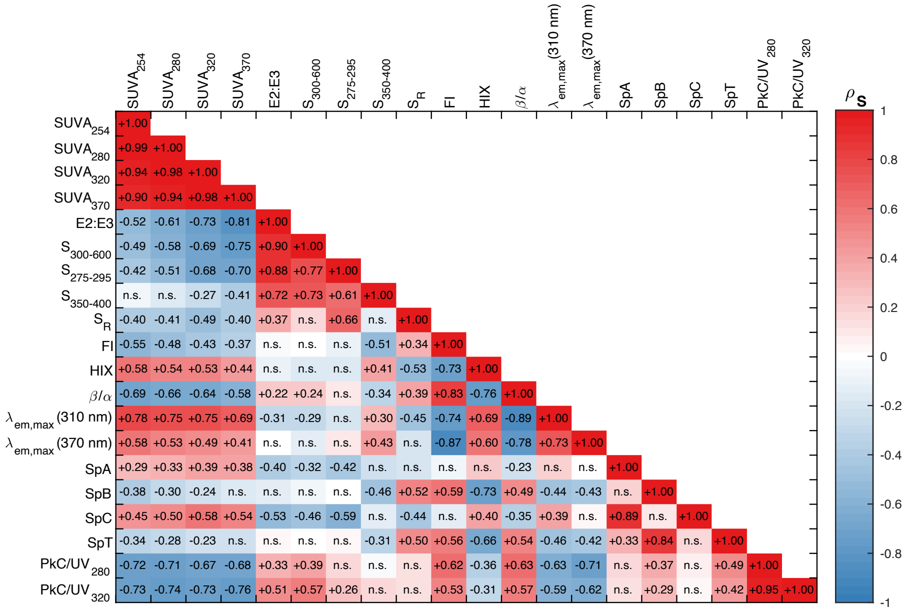

Figure S18. Heatmap of Spearman rho values (ρ_S) for all Natural samples in dataset. ρ_S values are printed only if the corresponding p_S was less than or equal to 0.01. If $p_S>0.01$, the color corresponding to the ρ_S value of the sample is still included, but n.s. (not significant) is printed.

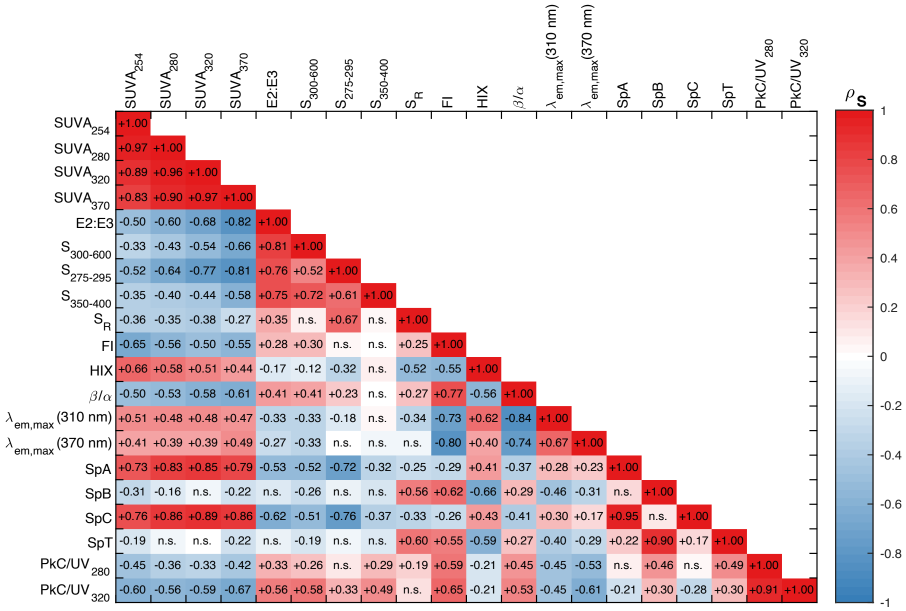

Figure S19. Heatmap of Spearman rho values (ρ_S) for all Treated samples in dataset. ρ_S values are printed only if the corresponding p_S was less than or equal to 0.01. If $p_S>0.01$, the color corresponding to the ρ_S value of the sample is still included, but n.s. (not significant) is printed.

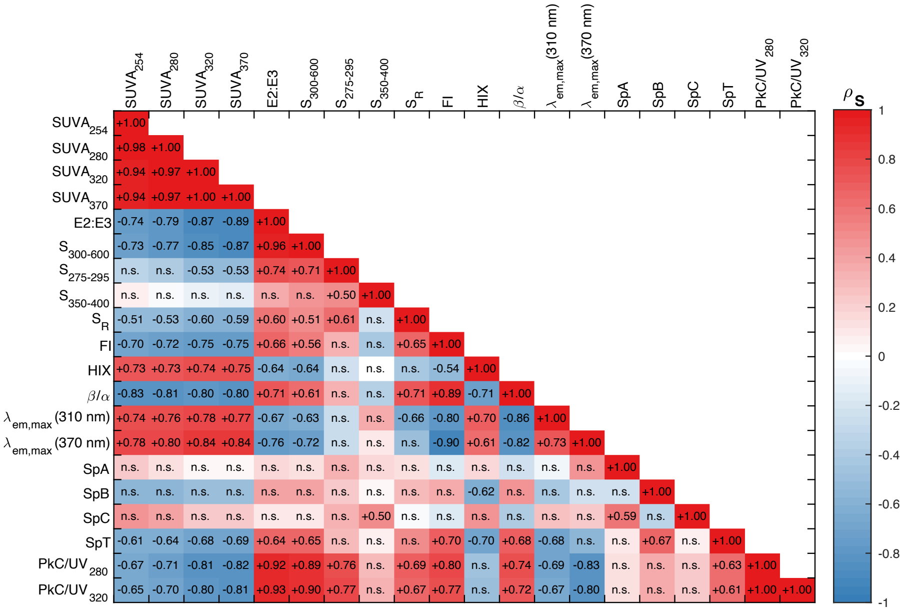

Figure S20. Heatmap of Spearman rho values (ρ_S) for all Isolate (Natural) samples in dataset. ρ_S values are printed only if the corresponding p_S was less than or equal to 0.01. If p_S>0.01, the color corresponding to the ρ_S value of the sample is still included, but n.s. (not significant) is printed.

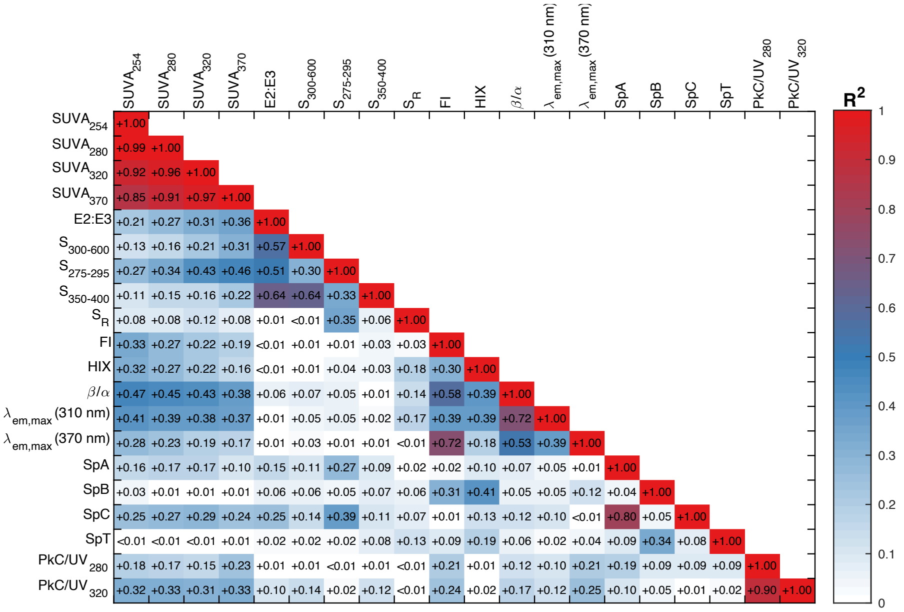

Figure S21. Heatmap of coefficient of determination (R^2) values for all samples in dataset. The color corresponds to the R^2 value, which is printed regardless of the correlation significance.

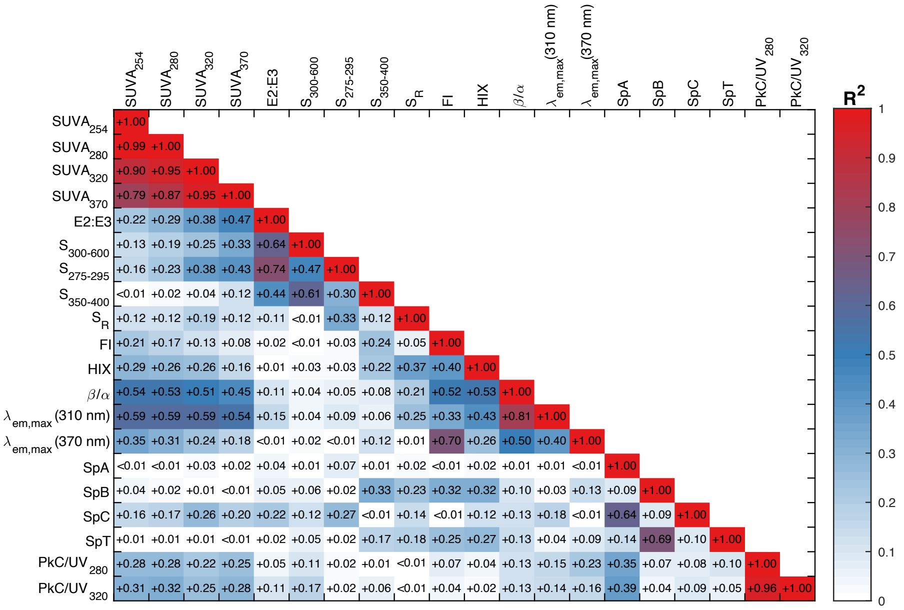

Figure S22. Heatmap of coefficient of determination (R^2) values for all Natural samples in dataset. The color corresponds to the R^2 value, which is printed regardless of the correlation significance.

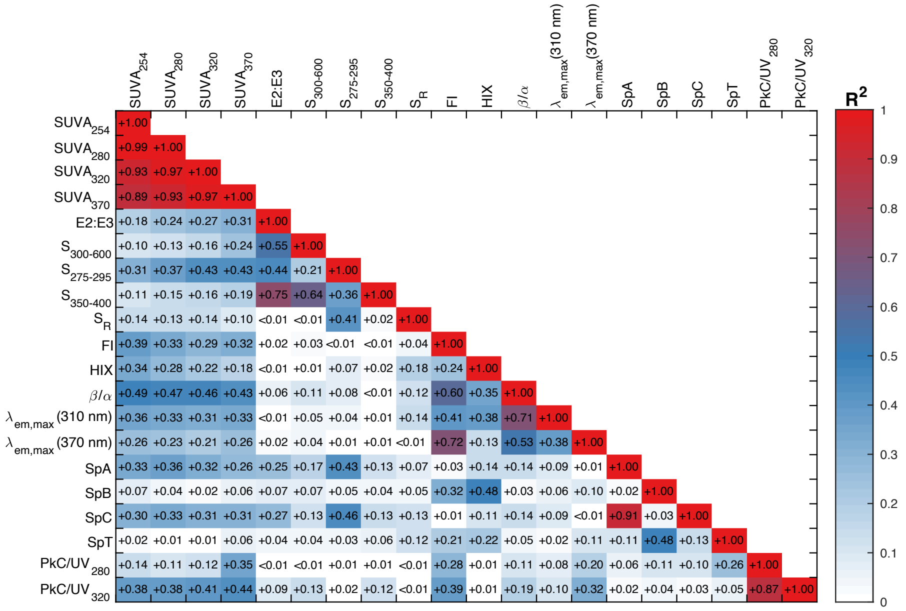

Figure S23. Heatmap of coefficient of determination (R^2) values for all Treated samples in dataset. The color corresponds to the R^2 value, which is printed regardless of the correlation significance.

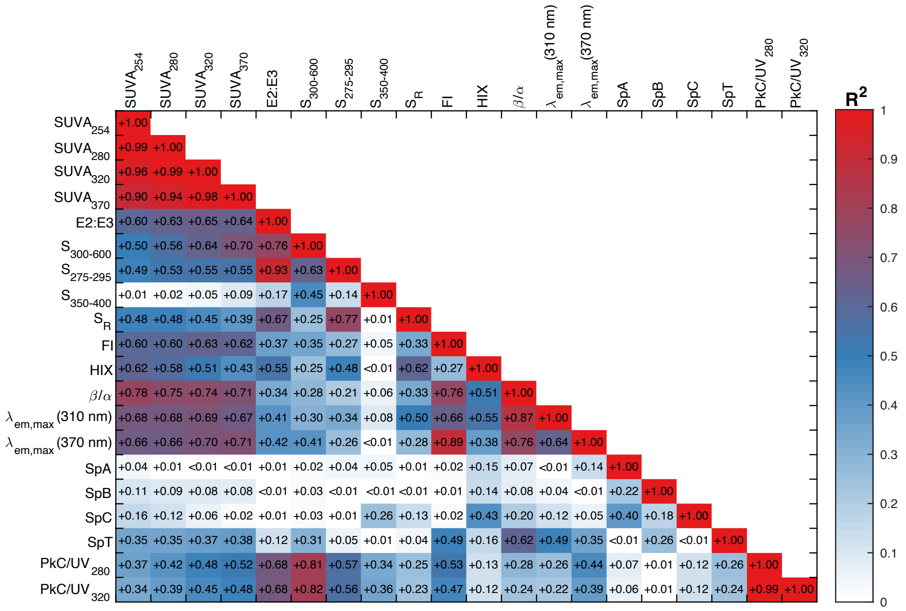

Figure S24. Heatmap of coefficient of determination (R^2) values for Isolate (Natural) samples in dataset. The color corresponds to the R^2 value, which is printed regardless of the correlation significance.

S4.2 Correlations between $S_{275-295}$, $S_{350-400}$, and S_R

The observation that E2:E3 has a higher frequency of strong correlations to $S_{300-600}$ (10 of 11) and $S_{275-295}$ (6 of 11) compared to $S_{350-400}$ (4 of 11) and S_R (1 of 11) (see Figure 2, main manuscript) begs the question of how $S_{275-295}$, $S_{300-600}$, $S_{350-400}$, and S_R are correlated to one another. This question is addressed in **Figure S25**. Across the meta-analysis dataset, $S_{275-295}$ had a higher frequency of strong and significant correlations to $S_{350-400}$ (3 of 12) and $S_{300-600}$ (4 of 12) relative to S_R (1 of 12). Although there is noticeable scatter in spectral slope across the meta-analysis dataset, individual subsets exhibit strong relationships, suggesting that spectral slopes calculated over different wavelength regions are tracking similar DOM pools.

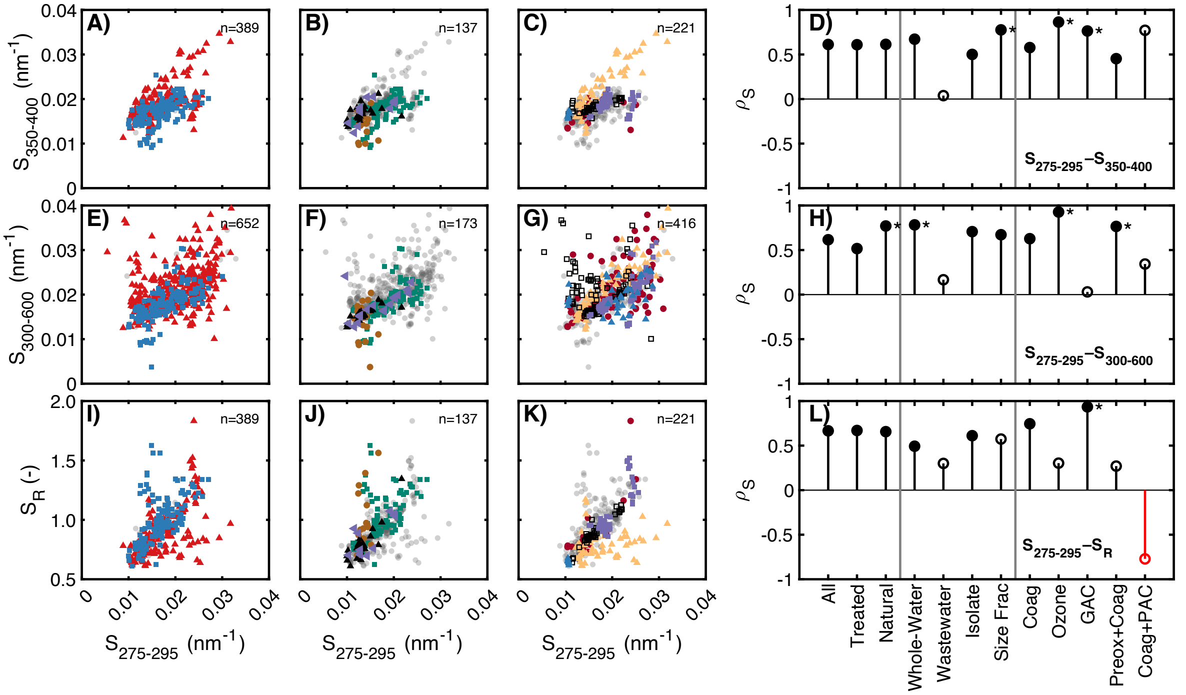

Figure S25. Relationships between the spectral slope between 275-295 nm ($S_{275-295}$) and other absorbance-based surrogates. Relationship between $S_{275-295}$ and A)–D) the spectral slope between 350-400 nm ($S_{350-400}$), E)–H) the spectral slope between 300-600 nm ($S_{300-600}$), and I)–L) the spectral slope ratio (S_R). The number of highlighted samples in each scatterplot is indicated by n. Lollipop plots in D), H), and L) show Spearman rho value (ρ_S) for optical surrogate correlations for specific data subsets. Closed symbols represent a significant relationship ($p_S<0.01$) while open symbols represent an insignificant relationship. Markers with an asterisk (*) indicate $|\rho_S|>0.75$. See **Figure S26** for symbol/color legend.

S4.3 Additional correlations between intrinsic optical surrogates

Figure S26 to **Figure S33** show results for correlations between intrinsic optical surrogates. Some SI Figures are referenced in the main manuscript but are not discussed explicitly here.

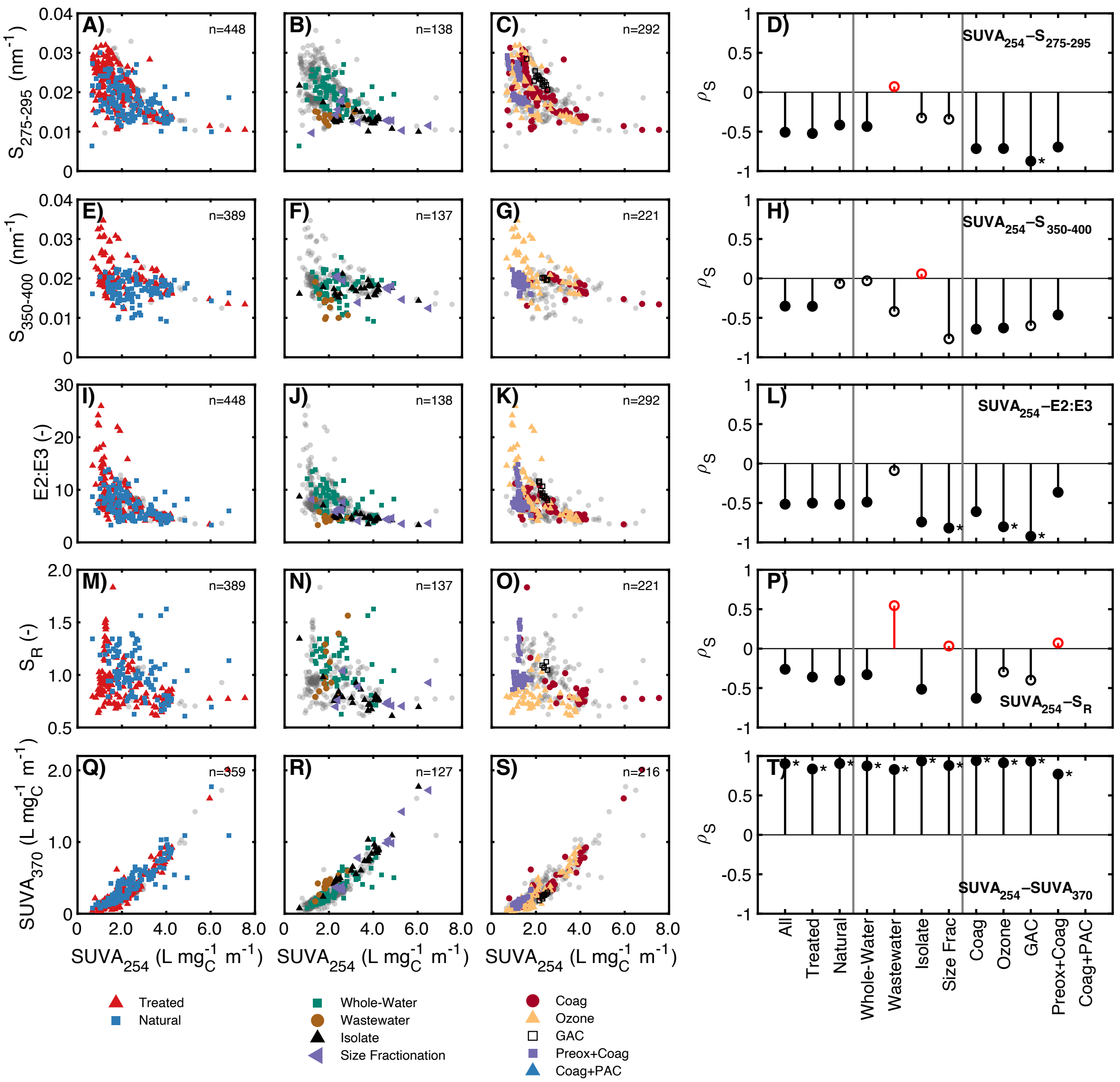

Figure S26. Relationships between the specific ultraviolet absorbance at 254 nm ($SUVA_{254}$) and other absorbance-based surrogates. Relationships between $SUVA_{254}$ and A)–D) spectral slope between 275 and 295 nm ($S_{275\text{-}295}$, nm^{-1}), E)–H) spectral slope between 350 and 400 nm ($S_{350\text{-}400}$, nm^{-1}), I)–L) E2:E3 ratio, M)–P) spectral slope ratio (S_R), and Q)–T) specific ultraviolet absorbance at 370 nm ($SUVA_{370}$). The number of highlighted samples in each scatterplot is indicated by n. Lollipop plots in D), H), L), P), and T) show Spearman rho value (ρ_S) for optical surrogate correlations for specific data subsets. Closed symbols represent a significant relationship (p_S<0.01) while open symbols represent an insignificant relationship. Markers with an asterisk (*) indicate $|\rho_S|$>0.75.

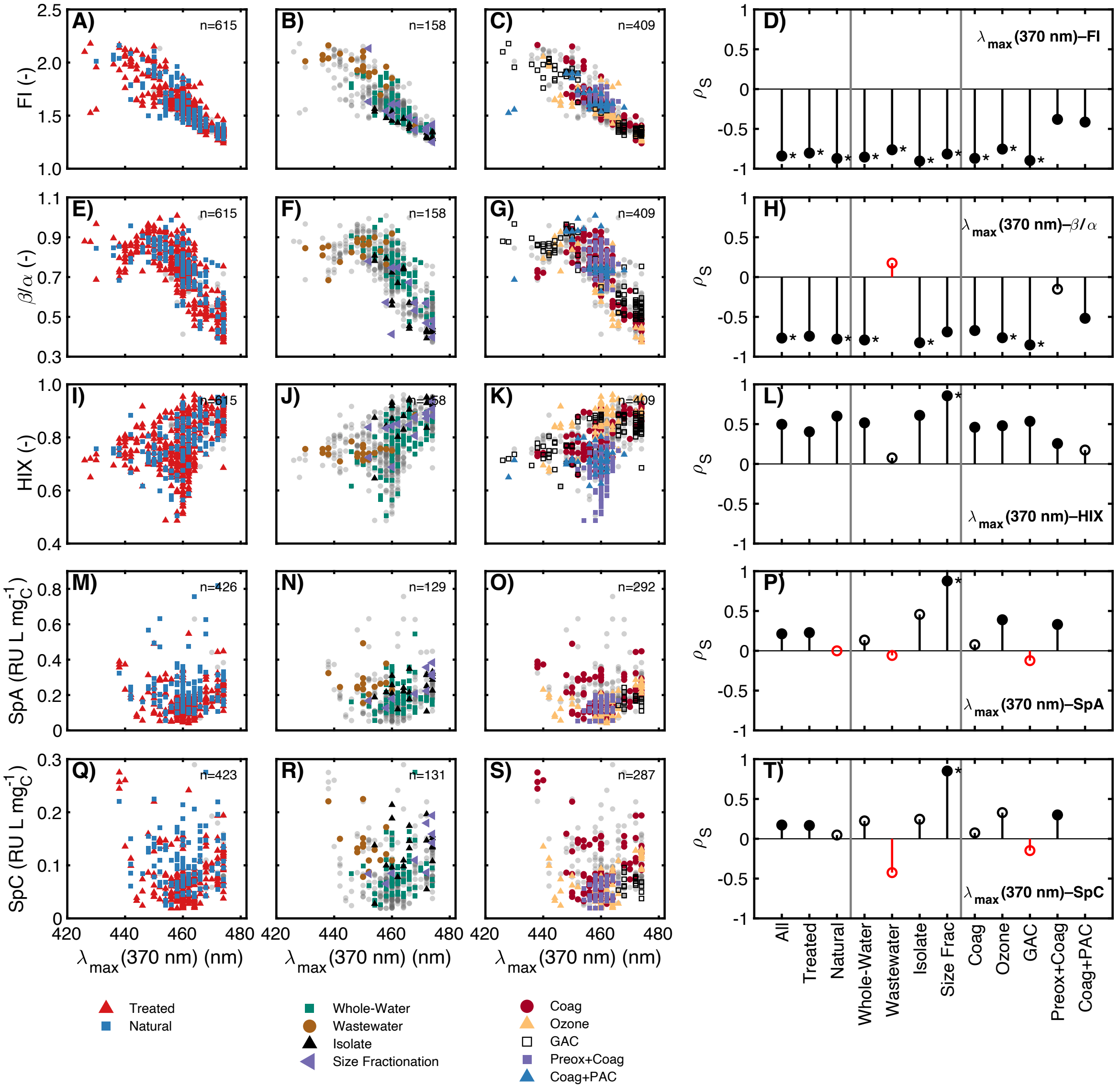

Figure S27. Relationships between the wavelength of maximum emission at 370 nm excitation ($\lambda_{max}(370nm)$) and fluorescence surrogates. Relationship between $\lambda_{max}(370nm)$ and A)–D) the fluorescence index, E)–H) biological index (β/α), I)–L) humification index (HIX), M)–P) specific peak A intensity (SpA), and Q)–T) specific peak C (SpC) intensity. The number of highlighted samples in each scatterplot is indicated by n. Lollipop plots in D), H), L), P), and T) show Spearman rho value (ρ_S) and p-values for ρ_S for correlations in specific data subsets. Closed symbols represent a significant relationship (p_S<0.01) while open symbols represent an insignificant relationship. Markers with an asterisk (*) indicate $|\rho_S|$>0.75.

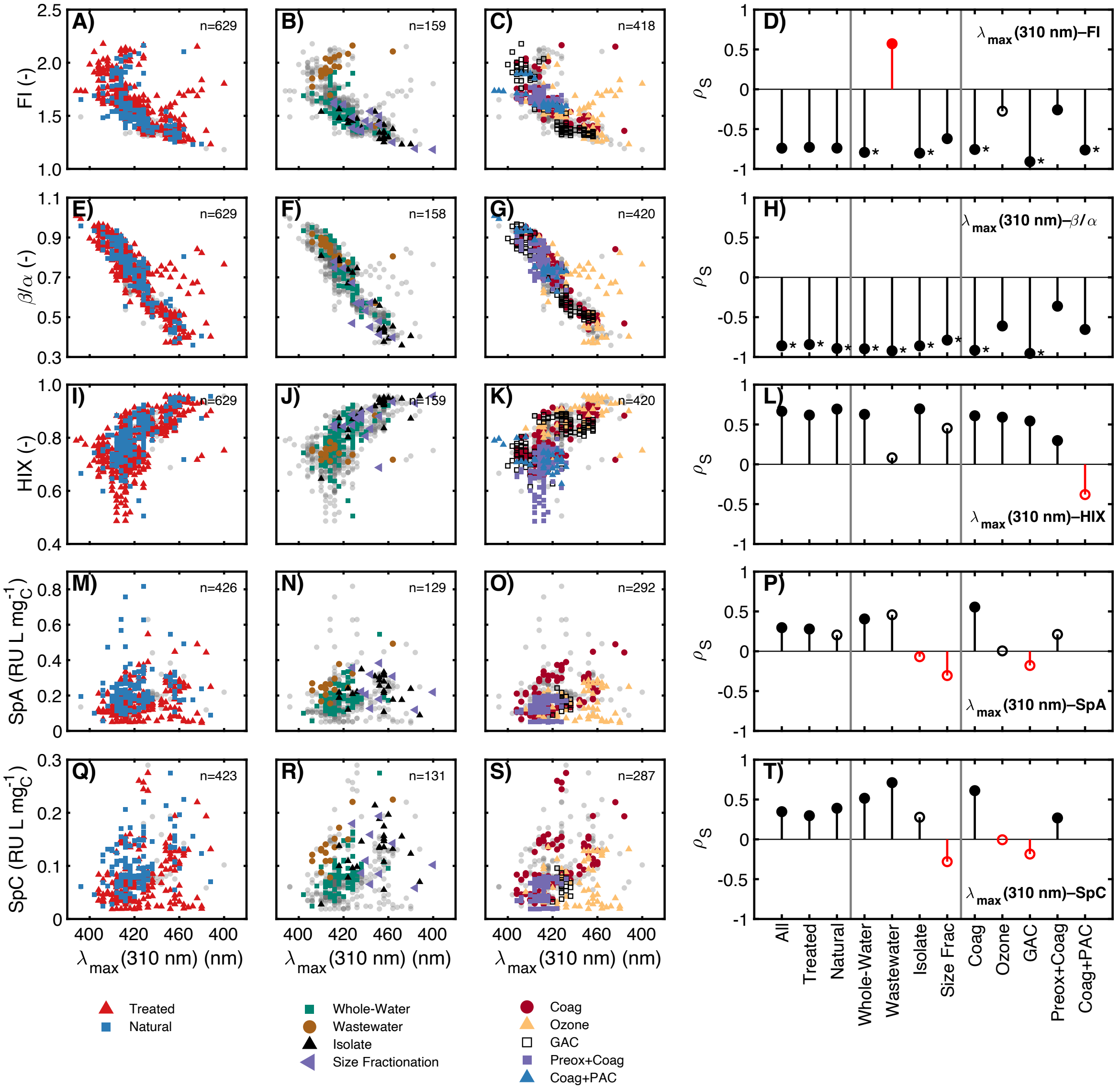

Figure S28. Relationships between the wavelength of maximum emission at 310 nm excitation ($\lambda_{max}(310nm)$) and fluorescence surrogates. Relationship between $\lambda_{max}(310nm)$ and A)–D) the fluorescence index (FI), E)–H) biological index (β/α), I)–L) humification index (HIX), M)–P) specific peak A (SpA) intensity, and Q)–T) specific peak C (SpC) intensity. The number of highlighted samples in each scatterplot is indicated by n. Lollipop plots in D), H), L), P), and T) show Spearman rho value (ρ_S) and p-values for ρ_S for correlations in specific data subsets. Closed symbols represent a significant relationship (p_S<0.01) while open symbols represent an insignificant relationship. Markers with an asterisk (*) indicate $|\rho_S|$>0.75

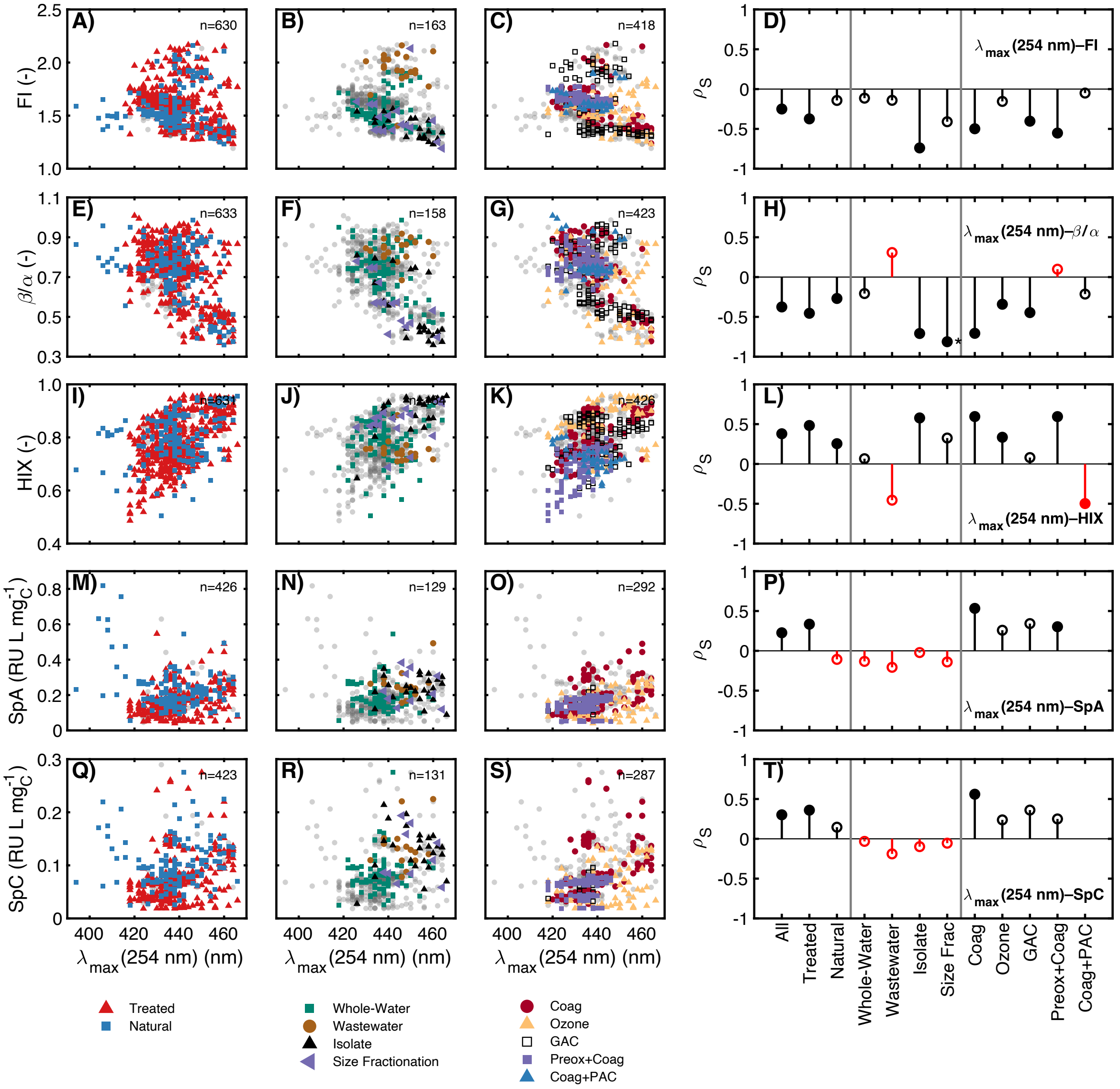

Figure S29. Relationships between the wavelength of maximum emission at 254 nm excitation ($\lambda_{max}(254nm)$) and fluorescence surrogates. Relationship between $\lambda_{max}(254nm)$ and A)–D) the fluorescence index (FI), E)–H) biological index (β/α), I)–L) humification index (HIX), M)–P) specific peak A (SpA) intensity, and Q)–T) specific peak C (SpC) intensity. The number of highlighted samples in each scatterplot is indicated by n. Lollipop plots in D), H), L), P), and T) show Spearman rho value (ρ_S) and p-values for ρ_S for correlations in specific data subsets. Closed symbols represent a significant relationship (p_S<0.01) while open symbols represent an insignificant relationship. Markers with an asterisk (*) indicate $|\rho_S|$>0.75

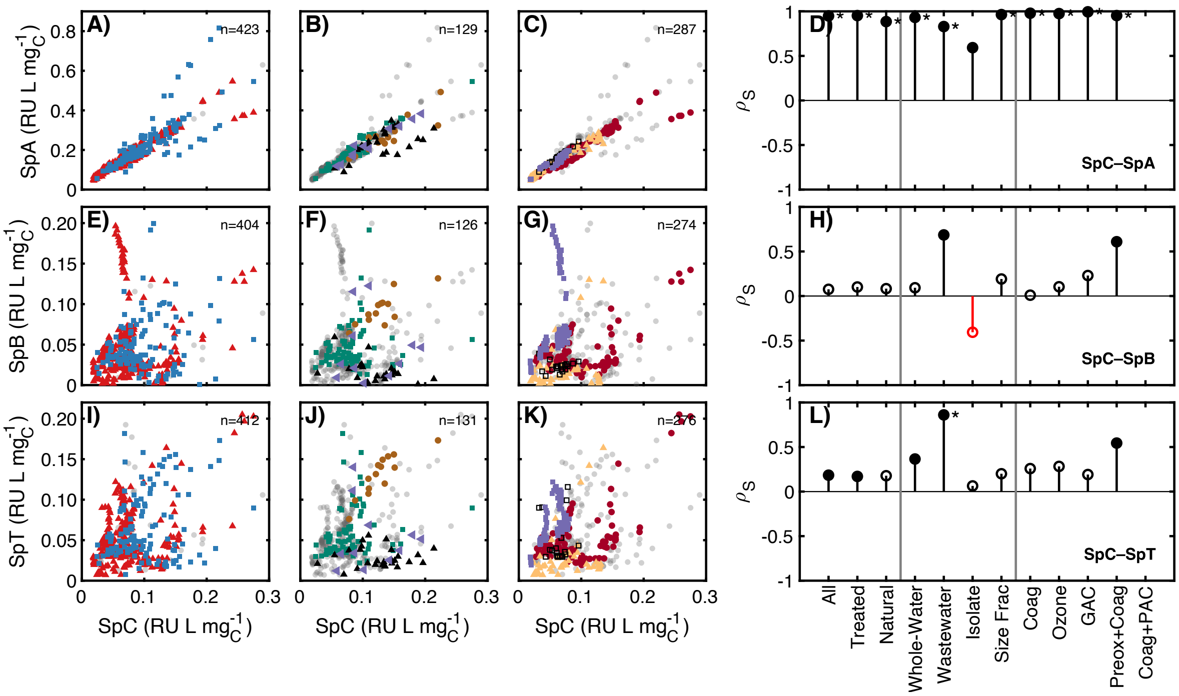

Figure S30. Relationships between specific peak intensities. Relationship between specific peak C (SpC) and A)–D) specific peak A (SpA), E)–H) specific peak B (SpB), I)–L) specific peak T (SpT). The number of highlighted samples in each scatterplot is indicated by n. Lollipop plots in D), H), and L) show Spearman rho value (ρ_S) and p-values for ρ_S for correlations in specific data subsets. Closed symbols represent a significant relationship (p_S<0.01) while open symbols represent an insignificant relationship. Markers with an asterisk (*) indicate $|\rho_S|$>0.75

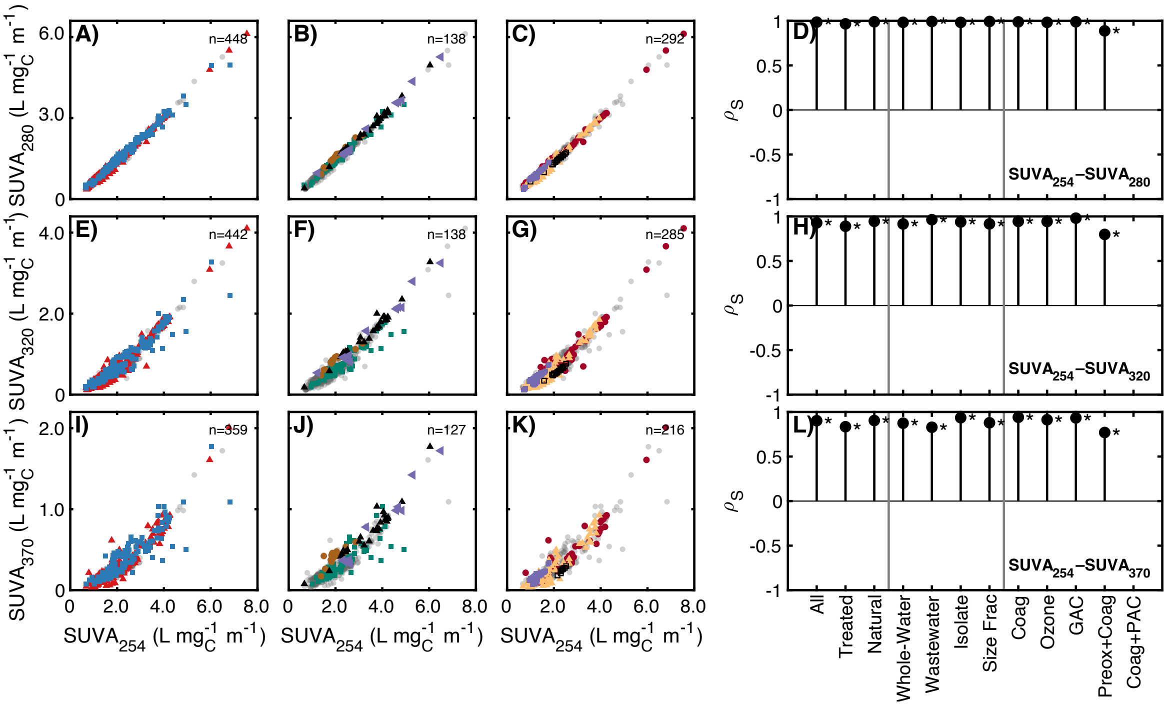

Figure S31. Relationships between the specific ultraviolet absorbance (SUVA) at different wavelengths. Relationship between SUVA at 254 nm ($SUVA_{254}$) and A)–D) specific ultraviolet absorbance at 280 nm ($SUVA_{280}$), E)–H) specific ultraviolet absorbance at 320 nm, and I)–L) specific ultraviolet absorbance at 370 nm. The number of highlighted samples in each scatterplot is indicated by n. Lollipop plots in D), H), and L) show Spearman rho value (ρ_S) and p-values for ρ_S for correlations in specific data subsets. Closed symbols represent a significant relationship (p_S<0.01) while open symbols represent an insignificant relationship. Markers with an asterisk (*) indicate $|\rho_S|$>0.75

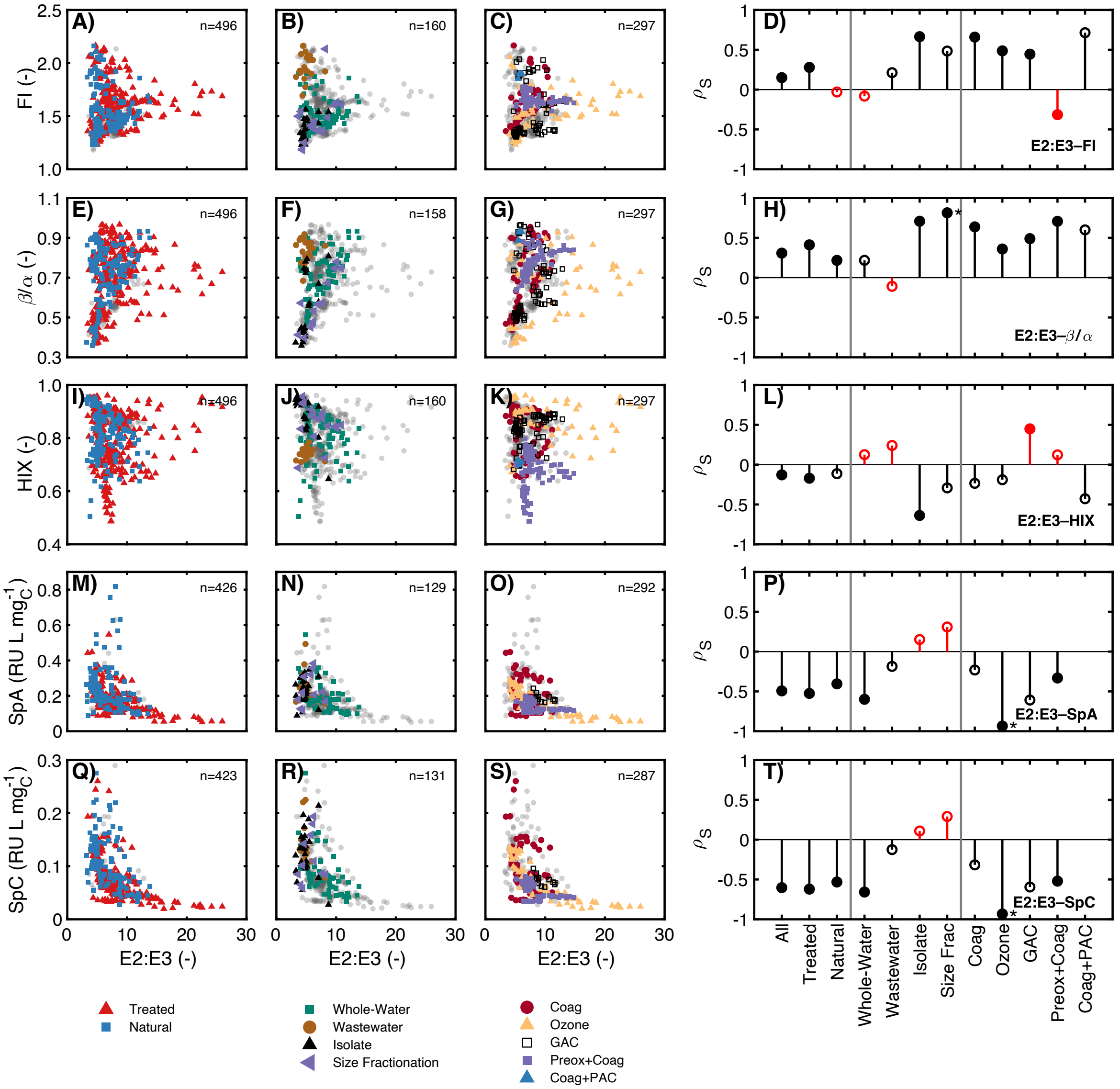

Figure S32. Relationships between E2:E3 and fluorescence surrogates. Relationship between E2:E3 and A)–D) the fluorescence index (FI), E)–H) biological index (β/α), I)–L) the humification index (HIX), M)–P) specific peak A (SpA), and Q)–T) specific peak C (SpC). The number of highlighted samples in each scatterplot is indicated by n. Lollipop plots in D), H), L), P), and T) show Spearman rho value (ρ_S) and p-values for ρ_S for correlations in specific data subsets. Closed symbols represent a significant relationship (p_S<0.01) while open symbols represent an insignificant relationship. Markers with an asterisk (*) indicate $|\rho_S|$>0.75.

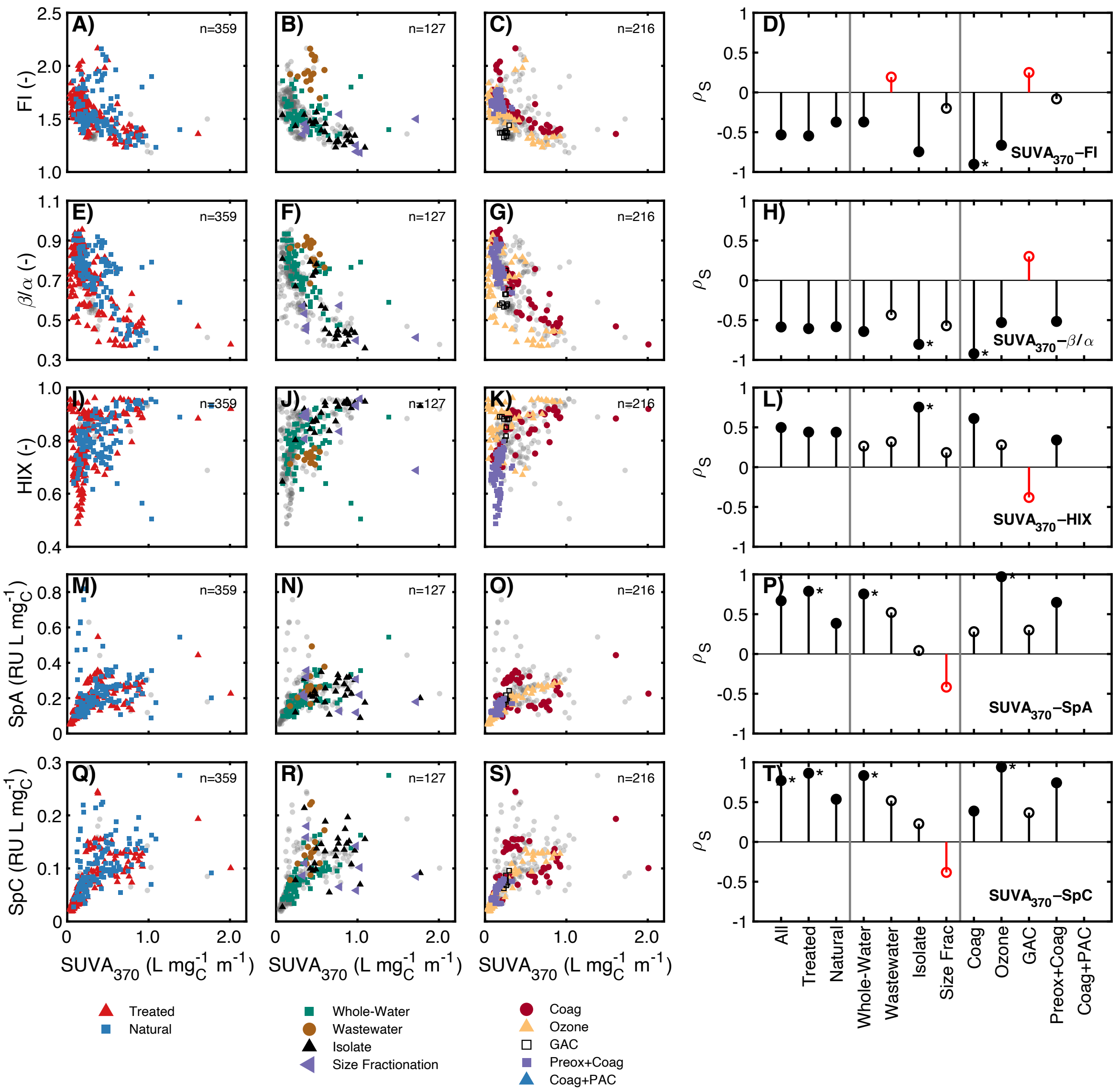

Figure S33. Relationships between the specific ultraviolet absorbance (SUVA) at 370 nm and fluorescence surrogates. Relationship between SUVA at 370 nm and A)–D) the fluorescence index (FI), E)–H) biological index (β/α), I)–L) the humification index (HIX), M)–P) specific peak A (SpA), and Q)–T) specific peak C (SpC). The number of highlighted samples in each scatterplot is indicated by n. Lollipop plots in D), H), L), P), and T) show Spearman rho value (ρ_S) and p-values for ρ_S for correlations in specific data subsets. Closed symbols represent a significant relationship (p_S<0.01) while open symbols represent an insignificant relationship. Markers with an asterisk (*) indicate $|\rho_S|$>0.75.

S4.4 Additional correlations between extrinsic optical surrogates

Although analysis of extrinsic correlations is not the focus of this study, it is of broad interest to evaluate the correlation of extrinsic surrogates (i.e., absorbance and fluorescence intensities) at different wavelengths to guide the design of *in situ* sensors for estimating DOC fluxes.[70-72] **Figure S34** to **Figure S36** show results for correlations between extrinsic optical surrogates. The strongest correlations were observed between absorbance at different wavelengths (e.g., UV_{254}–UV_{320}), between Peak A and Peak C, and between Peak C and OFI. Many of the data subsets exhibited strong correlations internally, but had a different trajectory based on original sample context. For example, correlations between different fluorescence peaks and correlations between absorbance and fluorescence peaks are weaker for isolates (**Figure S34 and S35**) relative to whole water samples. This is probably due in part to the absence of Peak B and T in many of the isolates. Treated samples underwent perturbations that resulted in concentration or absorptivity gradients during treatment. Some SI Figures are referenced in the main manuscript but are not discussed explicitly here.

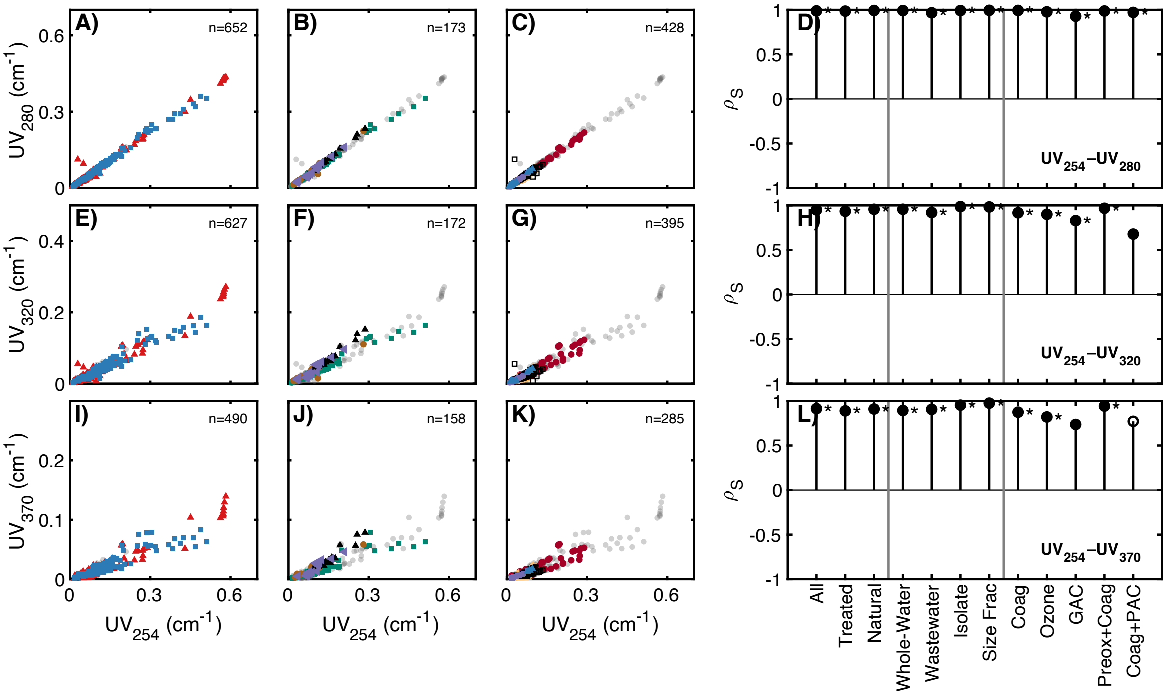

Figure S34. Relationships between UV absorbance at different wavelengths. Relationship between absorbance measured at 254 nm (UV_{254}) and A)–D) 280 nm (UV_{280}), E)–H) 320 nm (UV_{320}), and I)–L) 370 nm (UV_{370}). The number of highlighted samples in each scatterplot is indicated by n. Lollipop plots in D), H), and L) show Spearman rho value (ρ_S) and p-values for ρ_S for correlations in specific data subsets. Closed symbols represent a significant relationship (p_S<0.01) while open symbols represent an insignificant relationship. Markers with an asterisk (*) indicate $|\rho_S|$>0.75

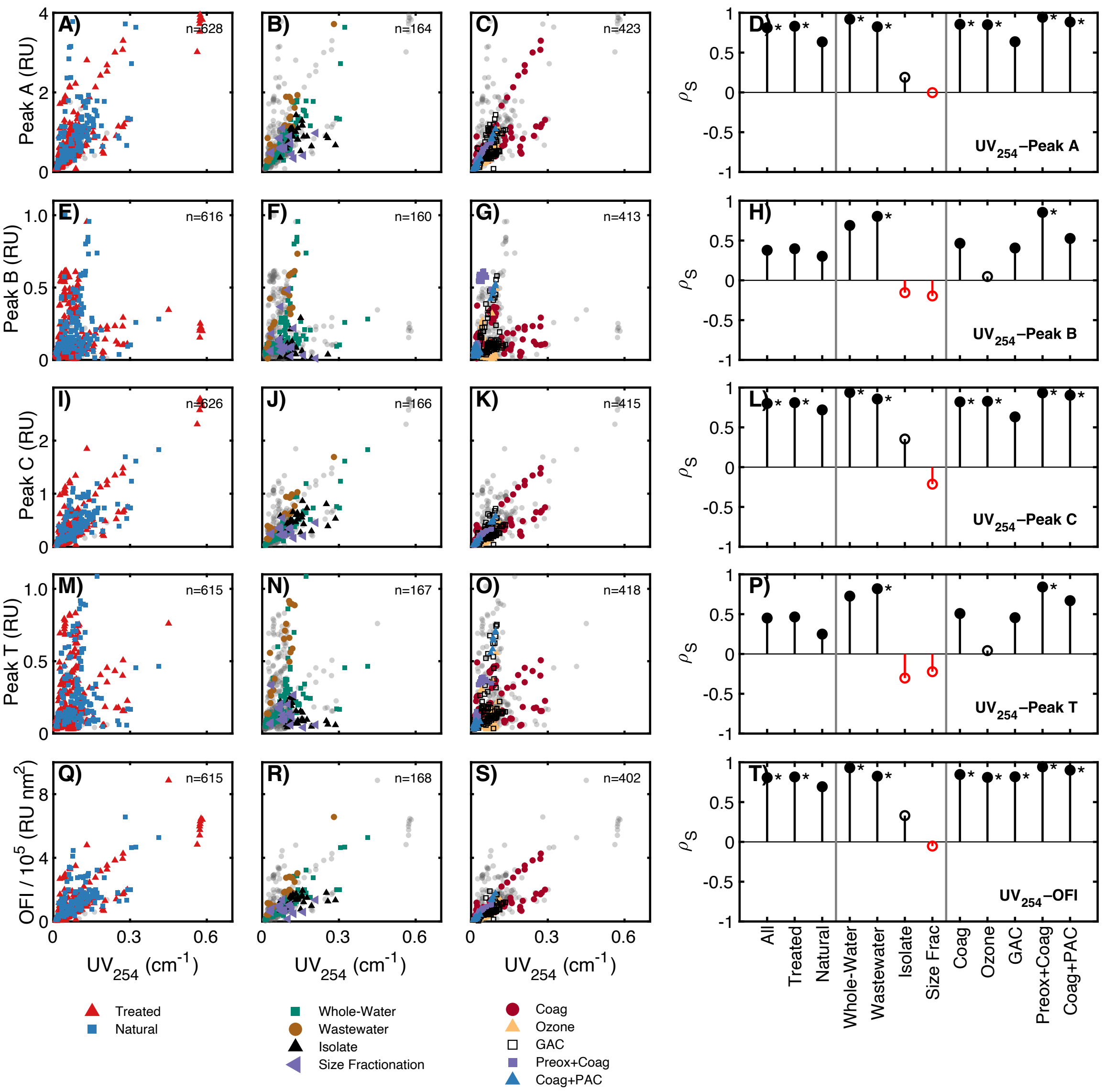

Figure S35. Relationship between UV absorbance at 254 nm and extrinsic fluorescence surrogates. Relationship between UV_{254} and A)–D) Peak A, E)–H) Peak B, I)–L) Peak C, M)–P) Peak T, and Q)–T) overall fluorescence intensity (OFI) divided by 10^5. Note samples with UV_{254} values >0.5 cm^{-1} are solutions of DOM isolate solutions; the treatment is pH titration. The number of highlighted samples in each scatterplot is indicated by n. Lollipop plots in D), H), L), P), and T) Spearman rho value (ρ_S) and p-values for ρ_S for correlations in specific data subsets. Closed symbols represent a significant relationship (p_S<0.01) while open symbols represent an insignificant relationship. Markers with an asterisk (*) indicate $|\rho_S|$>0.75.

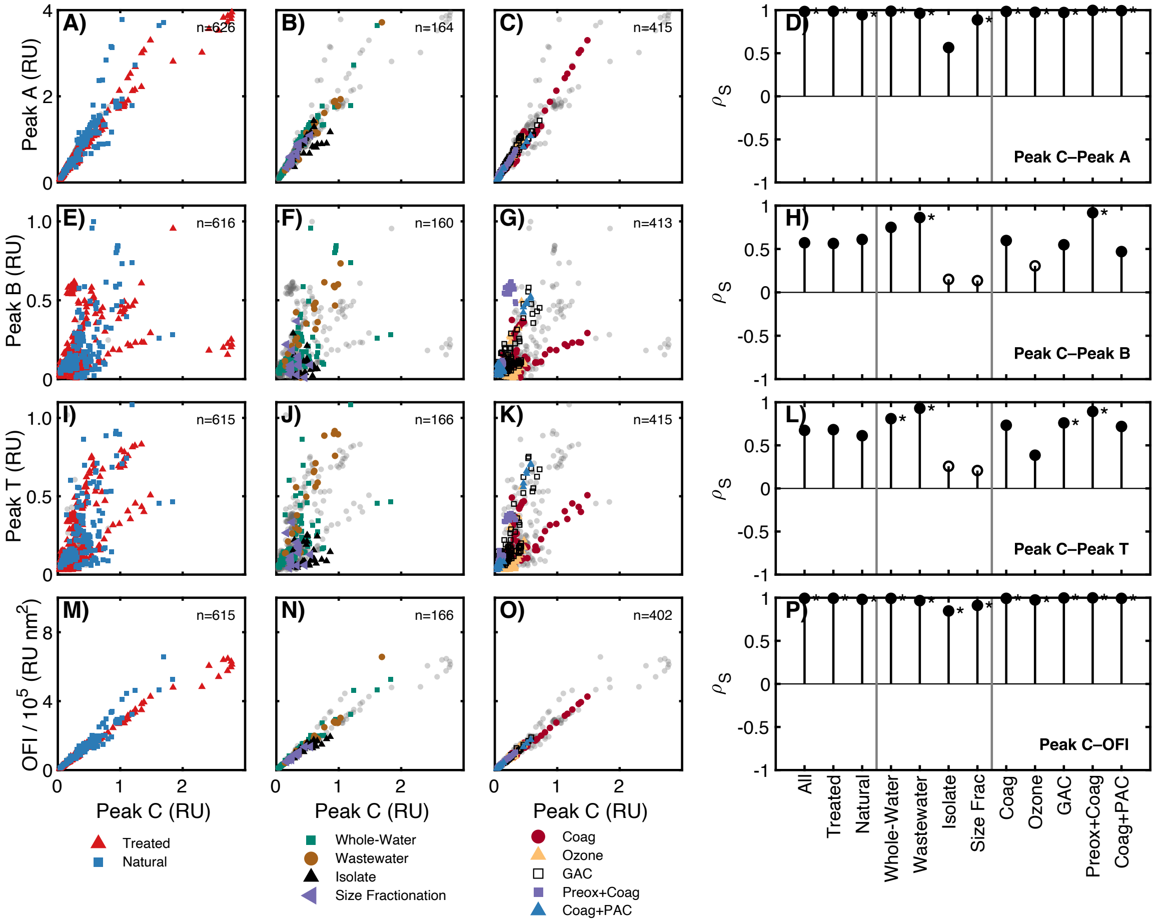

Figure S36. Relationships between fluorescence peak intensities. Relationship between Peak C intensity and A)–D) Peak A intensity, E)–H) Peak B intensity, I)–L) Peak T intensity, and M)–P) overall fluorescence intensity divided by 10^5. The number of highlighted samples in each scatterplot is indicated by n. Lollipop plots in D), H), L), and P) show Spearman rho value (ρ_S) and p-values for ρ_S for correlations in specific data subsets. Closed symbols represent a significant relationship (p_S<0.01) while open symbols represent an insignificant relationship. Markers with an asterisk (*) indicate $|\rho_S|$>0.75.

S4.5 Literature data

Figure S37 through **Figure S39** show results for correlations between intrinsic optical surrogates with literature data overlaid. Some SI Figures are referenced in the main manuscript but are not discussed explicitly here.

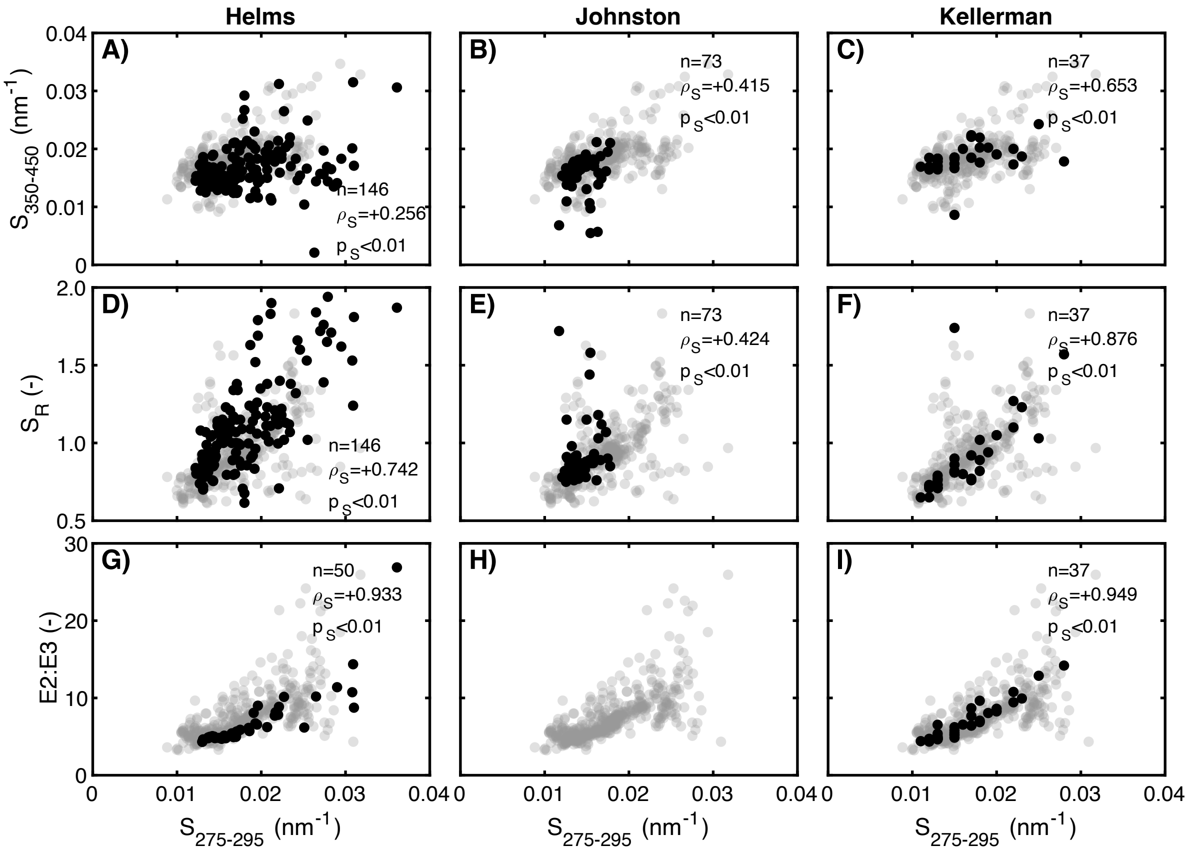

Figure S37. Relationship between literature-derived spectral slope between 275-295 ($S_{275-295}$) and other absorbance-based optical surrogates. Relationship between $S_{275-295}$ and A)–C) spectral slope between 350-400 nm ($S_{350-400}$), D)–F) spectral slope ratio (S_R), and G)–I) E2:E3. The number of samples with paired optical surrogates (n), their Spearman rho value (ρ_S), and p-value for ρ_S are shown for each correlation. The title of each column represents the literature reference: Helms = Helms et al. (2008),[8] Johnston = Johnston et al. (2021),[65] Kellerman = Kellerman et al. (2018).[54]

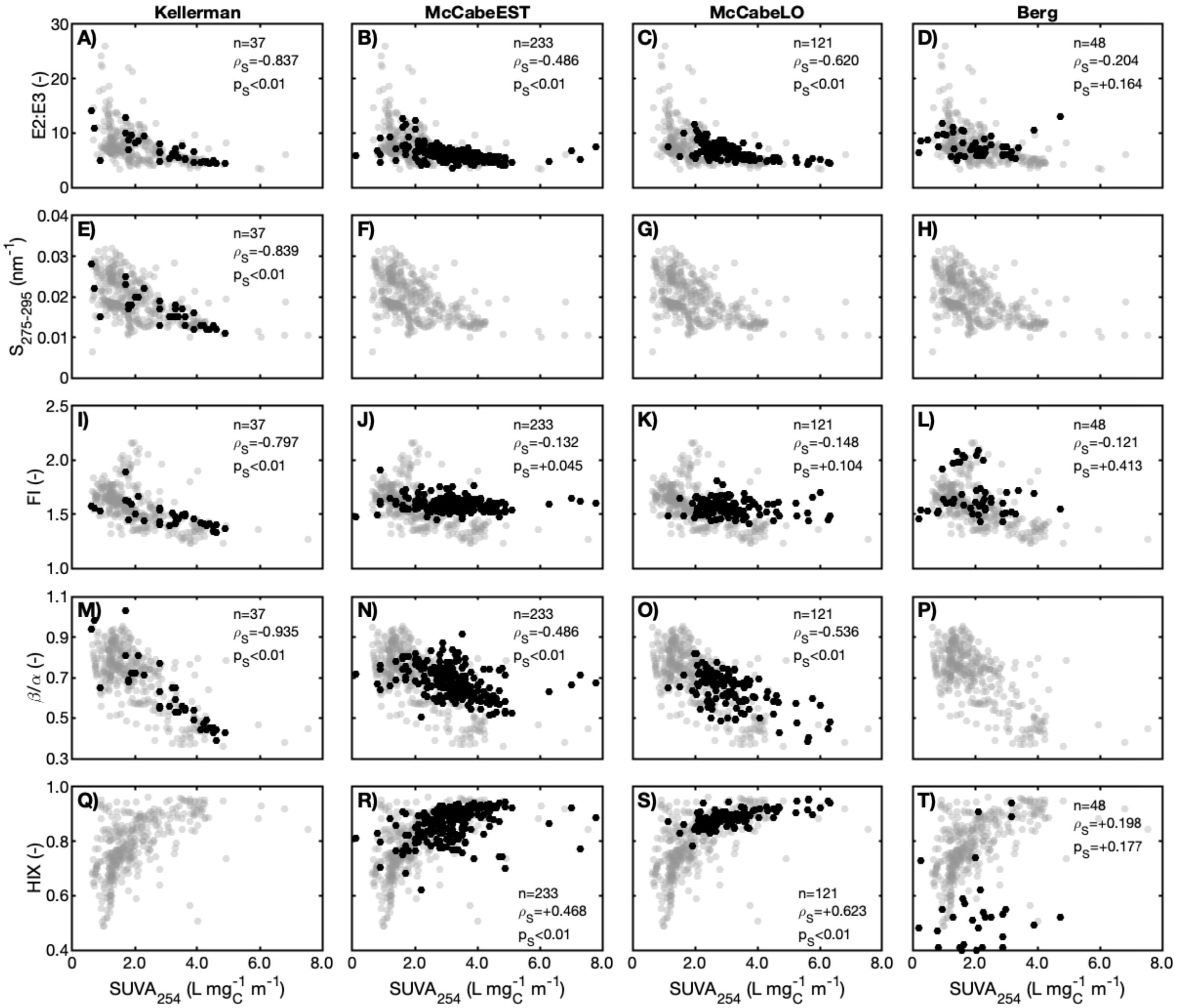

Figure S38. Relationship between literature-derived specific ultraviolet absorbance (SUVA) at 254 nm and other optical surrogates. Relationship between SUVA at 254 nm and A)–D) E2:E3, E)–H) spectral slope between 275-295 nm ($S_{275\text{-}295}$), I)–L) fluorescence index (FI), M)–P) biological index (β/α), and Q)–T) humification index (HIX). The number of samples with paired optical surrogates (n), their Spearman rho value (ρ_S), and p-value for ρ_S are shown for each correlation. The title of each column represents the literature reference: Kellerman = Kellerman et al. (2018),[54] McCabeEST = McCabe and Arnold (2017),[55] McCabeLO = McCabe and Arnold (2018),[56] and Berg = Berg et al. (2023).[57]

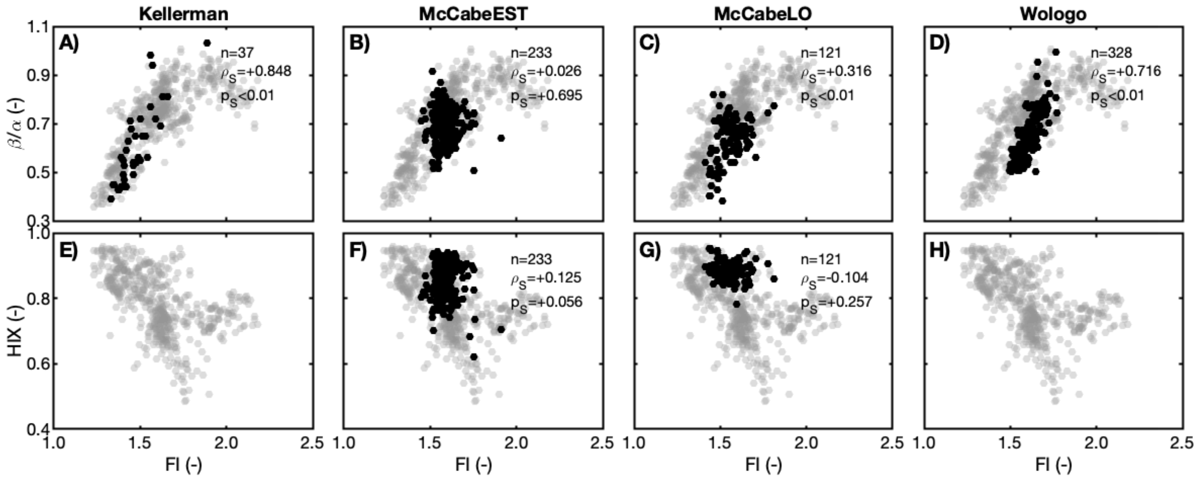

Figure S39. Relationship between literature-derived fluorescence index (FI) and other fluorescence-based surrogates. Relationships between FI and A)–D) biological index (β/α) and E)–H) humification index (HIX).The number of samples with paired optical surrogates (n), their Spearman rho value (ρ_S), and p-value for ρ_S are shown for each correlation. The title of each column represents the literature reference: Kellerman = Kellerman et al. (2018),[54] McCabeEST = McCabe and Arnold (2017),[55] McCabeLO = McCabe and Arnold (2018),[56] and Wologo = Wologo et al. (2021).[63]

S 5 References

1. Shapiro, J., Chemical and Biological Studies on the Yellow Organic Acids of Lake Water. *Limnology and Oceanography* **1957,** *2* (3), 161-179.

2. Traina, S. J.; Novak, J.; Smeck, N. E., An Ultraviolet Absorbance Method of Estimating the Percent Aromatic Carbon Content of Humic Acids. *Journal of Environmental Quality* **1990,** *19* (1), 151-153.

3. Chin, Y.-P.; Aiken, G.; O'Loughlin, E., Molecular weight, polydispersity, and spectroscopic properties of aquatic humic substances. *Environmental Science & Technology* **1994,** *26*, 1853-1858.

4. Peuravuori, J.; Pihlaja, K., Molecular size distribution and spectroscopic properties of aquatic humic substances. *Analytica Chimica Acta* **1997,** *337*, 133-149.

5. Weishaar, J. L.; Aiken, G. R.; Bergamaschi, B. A.; Fram, M. S.; Fujii, R.; Mopper, K., Evaluation of specific ultraviolet absorbance as an indicator of the chemical composition and reactivity of dissolved organic carbon. *Environmental Science & Technology* **2003,** *37* (20), 4702-4708.

6. De Haan, H.; Werlemark, G.; De Boer, T., Effect of pH on molecular weight and size of fulvic acids in drainage water from peaty grassland in NW Netherlands. *Plant and Soil* **1983,** *75* (1), 63-73.

7. Stedmon, C. A.; Markager, S.; Kaas, H., Optical Properties and Signatures of Chromophoric Dissolved Organic Matter (CDOM) in Danish Coastal Waters. *Estuarine, Coastal and Shelf Science* **2000,** *51* (2), 267-278.

8. Helms, J. R.; Stubbins, A.; Ritchie, J. D.; Minor, E. C.; Kieber, D. J.; Mopper, K., Absorption spectral slopes and slope ratios as indicators of molecular weight, source, and photobleaching of chromophoric dissolved organic matter. *Limnology and Oceanography* **2008,** *53* (3), 955-969.

9. Summers, R. S.; Cornel, P. K.; Roberts, P. V., Molecular size distribution and spectroscopic characterization of humic substances. *Science of The Total Environment* **1987,** *62*, 27-37.

10. Haan, H. D., Some structural and ecological studies on soluble humic compounds from Tjeukemeer. *Int Vereinigung Für Theor Und Angewandte Limnologie Verhandlungen* **1972,** *18* (2), 685-695.

11. Bodhipaksha, L. C.; Sharpless, C. M.; Chin, Y.-P.; Sander, M.; Langston, W. K.; MacKay, A. A., Triplet Photochemistry of Effluent and Natural Organic Matter in Whole Water and Isolates from Effluent-Receiving Rivers. *Environmental Science & Technology* **2015,** *49* (6), 3453-3463.

12. Wallin, M. B.; Weyhenmeyer, G. A.; Bastviken, D.; Chmiel, H. E.; Peter, S.; Sobek, S.; Klemedtsson, L., Temporal control on concentration, character, and export of dissolved organic carbon in two hemiboreal headwater streams draining contrasting catchments. *J Geophys Res-Biogeo* **2015,** *120* (5), 832-846.

13. Sharpless, C. M.; Aeschbacher, M.; Page, S. E.; Wenk, J.; Sander, M.; McNeill, K., Photooxidation-Induced Changes in Optical, Electrochemical, and Photochemical Properties of Humic Substances. *Environmental Science & Technology* **2014,** *48* (5), 2688-2696.

14. Helms, J. R.; Stubbins, A.; Perdue, E. M.; Green, N. W.; Chen, H.; Mopper, K., Photochemical bleaching of oceanic dissolved organic matter and its effect on absorption spectral slope and fluorescence. *Mar. Chem.* **2013,** *155*, 81-91.

15. Fichot, C. G.; Benner, R., The spectral slope coefficient of chromophoric dissolved organic matter as a tracer of terrigenous dissolved organic carbon in river-influenced ocean margins. *Limnology and Oceanography* **2012,** *57* (5), 1453-1466.

16. Stedmon, C. A.; Markager, S.; Kaas, H., Optical properties and signatures of chromophoric dissolved organic matter (CDOM) in Danish coastal waters. *Estuar Coast Shelf S* **2000,** *51* (2), 267-278.

17. Kowalczuk, P.; Ston-Egiert, J.; Cooper, W. J.; Whitehead, R. F.; Durako, M. J., Characterization of chromophoric dissolved organic matter (CDOM) in the Baltic Sea by excitation emission matrix fluorescence spectroscopy. *Marine Chemistry* **2005,** *96* (3-4), 273-292.

18. Twardowski, M. S.; Boss, E.; Sullivan, J. M.; Donaghay, P. L., Modeling the spectral shape of absorption by chromophoric dissolved organic matter. *Marine Chemistry* **2004,** *89* (1-4), 69-88.

19. Zsolnay; Baigar; Jimenez; Steinweg; Saccomandi, Differentiating with fluorescence spectroscopy the sources of dissolved organic matter in soils subjected to drying. *Chemosphere* **1999,** *38* (1), 45-50.

20. Wilson, H. F.; Xenopoulos, M. A., Effects of agricultural land use on the composition of fluvial dissolved organic matter. *Nature Geoscience* **2009,** *2* (1), 37-41.

21. Huguet, A.; Vacher, L.; Relexans, S.; Saubusse, S.; Froidefond, J. M.; Parlanti, E., Properties of fluorescent dissolved organic matter in the Gironde Estuary. *Org. Geochem.* **2009,** *40* (6), 706-719.

22. Cory, R. M.; Miller, M. P.; McKnight, D. M.; Guerard, J. J.; Miller, P. L., Effect of instrument-specific response on the analysis of fulvic acid fluorescence spectra. *Limnology and Oceanography: Methods* **2010,** *8* (2), 67-78.

23. McKnight, D. M.; Boyer, E. W.; Westerhoff, P. K.; Doran, P. T.; Kulbe, T.; Andersen, D. T., Spectrofluorometric characterization of dissolved organic matter for indication of precursor organic material and aromaticity. *Limnology and Oceanography* **2001,** *46* (1), 38-48.

24. Alberts, J. J.; Takács, M., Total luminescence spectra of IHSS standard and reference fulvic acids, humic acids and natural organic matter:: comparison of aquatic and terrestrial source terms. *Organic Geochemistry* **2004,** *35* (3), 243-256.

25. Jaffé, R.; Boyer, J. N.; Lu, X.; Maie, N.; Yang, C.; Scully, N. M.; Mock, S., Source characterization of dissolved organic matter in a subtropical mangrove-dominated estuary by fluorescence analysis. *Marine Chemistry* **2004,** *84* (3-4), 195-210.

26. Hudson, N.; Baker, A.; Reynolds, D., Fluorescence analysis of dissolved organic matter in natural, waste and polluted waters - A review. *River Res Appl* **2007,** *23* (6), 631-649.

27. Korak, J. A.; Dotson, A. D.; Summers, R. S.; Rosario-Ortiz, F. L., Critical analysis of commonly used fluorescence metrics to characterize dissolved organic matter. *Water research* **2014,** *49*, 327-338.

28. Ohno, T., Fluorescence inner-filtering correction for determining the humification index of dissolved organic matter. *Environmental Science & Technology* **2002,** *36* (4), 742-746.

29. Zepp, R. G.; Sheldon, W. M.; Moran, M. A., Dissolved organic fluorophores in southeastern US coastal waters: correction method for eliminating Rayleigh and Raman scattering peaks in excitation-emission matrices. *Marine Chemistry* **2004,** *89* (1-4), 15-36.

30. Fellman, J. B.; Hood, E.; Spencer, R. G. M., Fluorescence spectroscopy opens new windows into dissolved organic matter dynamics in freshwater ecosystems: A review. *Limnology and Oceanography* **2010,** *55* (6), 2452-2462.

31. Gabor, R. S.; Baker, A.; McKnight, D. M.; Miller, M. P., Fluorescence Indices and Their Interpretation. *Camb Env Ch* **2014**, 303-338.

32. Parlanti, E.; Wörz, K.; Geoffroy, L.; Lamotte, M., Dissolved organic matter fluorescence spectroscopy as a tool to estimate biological activity in a coastal zone submitted to anthropogenic inputs. *Organic Geochemistry* **2000,** *31* (12), 1765-1781.

33. Huguet, A.; Vacher, L.; Saubusse, S.; Etcheber, H.; Abril, G.; Relexans, S.; Ibalot, F.; Parlanti, E., New insights into the size distribution of fluorescent dissolved organic matter in estuarine waters. *Organic Geochemistry* **2010,** *41* (6), 595-610.

34. Coble, P. G., Characterization of marine and terrestrial DOM in seawater using excitation emission matrix spectroscopy. *Marine Chemistry* **1996,** *51* (4), 325-346.

35. Mostafa, S.; Korak, J. A.; Shimabuku, K.; Glover, C. M.; Rosario-Ortiz, F. L., Relation between optical properties and Formation of reactive intermediates from different size fractions of organic matter. In *Advances in the Physicochemical Characterization of Dissolved Organic Matter: Impact on Natural and Engineered Systems*, American Chemical Society: Washington, DC, 2014; Vol. 1160, pp 159-179.

36. McKay, G.; Korak, J. A.; Erickson, P. R.; Latch, D. E.; McNeill, K.; Rosario-Ortiz, F. L., The case against charge transfer interactions in dissolved organic matter photophysics. *Environmental Science & Technology* **2018,** *52* (2), 406-414.

37. Shimabuku, K. K.; Kennedy, A. M.; Mulhern, R. E.; Summers, R. S., Evaluating Activated Carbon Adsorption of Dissolved Organic Matter and Micropollutants Using Fluorescence Spectroscopy. *Environmental Science & Technology* **2017,** *51* (5), 2676-2684.

38. Li, H.; McKay, G., Relationships between the Physicochemical Properties of Dissolved Organic Matter and Its Reaction with Sodium Borohydride. *Environmental Science & Technology* **2021,** *55* (15), 10843-10851.

39. Korak, J. A.; Rosario-Ortiz, F. L.; Summers, R. S., Evaluation of optical surrogates for the characterization of DOM removal by coagulation. *Environmental Science: Water Research & Technology* **2015,** *1*, 493-506.

40. Korak, J. A.; Rosario-Ortiz, F. L.; Summers, R. S., Fluorescence Characterization of Humic Substance Coagulation: Application of New Tools to an Old Process. In *Advances in the Physicochemical Characterization of Dissolved Organic Matter: Impact on Natural and Engineered Systems*, American Chemical Society: Washington, DC, 2014; Vol. 1160, pp 281-300.

41. McKay, G.; Korak, J. A.; Rosario-Ortiz, F. L., Temperature Dependence of Dissolved Organic Matter Fluorescence. *Environmental Science & Technology* **2018,** *52* (16), 9022-9032.

42. McKay, G.; Huang, W.; Romera-Castillo, C.; Crouch, J. E.; Rosario-Ortiz, F. L.; Jaffé, R., Predicting Reactive Intermediate Quantum Yields from Dissolved Organic Matter Photolysis Using Optical Properties and Antioxidant Capacity. *Environmental Science & Technology* **2017,** *51* (10), 5404-5413.

43. McKay, G.; Couch, K. D.; Mezyk, S. P.; Rosario-Ortiz, F. L., Investigation of the coupled effects of molecular weight and charge-transfer interactions on the optical and photochemical properties of dissolved organic matter. *Environmental Science & Technology* **2016,** *50* (15), 8093-8102.

44. Önnby, L.; Salhi, E.; McKay, G.; Rosario-Ortiz, F. L.; von Gunten, U., Ozone and chlorine reactions with dissolved organic matter - Assessment of oxidant-reactive moieties by optical measurements and the electron donating capacities. *Water research* **2018,** *144*, 64-75.

45. Leresche, F.; McKay, G.; Kurtz, T.; von Gunten, U.; Canonica, S.; Rosario-Ortiz, F. L., Effects of ozone on the photochemical and photophysical properties of dissolved organic matter. *Environmental Science & Technology* **2019,** *53* (10), 5622-5632.

46. Shimabuku, K. K.; Cho, H.; Townsend, E. B.; Rosario-Ortiz, F. L.; Summers, R. S., Modeling Nonequilibrium Adsorption of MIB and Sulfamethoxazole by Powdered Activated Carbon and the Role of Dissolved Organic Matter Competition. *Environmental Science & Technology* **2014,** *48* (23), 13735-13742.

47. Hohner, A. Source Water Quality Characteristics and Implications for Disinfection Byproduct Formation in Colorado Surface Waters. University of Colorado Boulder, 2011.

48. Shimabuku, K. K. Biochar Sorbents for the Control of Organic Contaminants in Stormwater: Understanding Biochar Structure and Water Quality on Sorption Behavior. University of Colorado Boulder, 2017.

49. Korak, J. A.; Wert, E. C.; Rosario-Ortiz, F. L., Fluorescence as a Surrogate for the Release of Intracellular Material From Cyanobacteria. *Journal AWWA* **2015,** *107* (10), E523-E532.

50. Kaplan, J. Modeling Natural Organic Matter Removal Using Absorbance and Fluorescence Spectroscopy: Effect of Coagulant and Pre-Oxidant. Masters Thesis, University of Colorado Boulder, 2020.

51. Allen, A.; Cheng, K.; McKay, G., Evaluating the pH-dependence of DOM absorbance, fluorescence, and photochemical production of singlet oxygen. *Environmental Science: Processes & Impacts* **2023,** *25*, 1974-1985.

52. Poulin, B. A.; Ryan, J. N.; Aiken, G. R., Effects of iron on optical properties of dissolved organic matter. *Environmental Science & Technology* **2014,** *48* (17), 10098-10106.

53. Mack, J.; Bolton, J. R., Photochemistry of nitrite and nitrate in aqueous solution: a review. *Journal of Photochemistry and Photobiology* **1999,** *128* (1), 1-13.

54. Kellerman, A. M.; Guillemette, F.; Podgorski, D. C.; Aiken, G. R.; Butler, K. D.; Spencer, R. G. M., Unifying Concepts Linking Dissolved Organic Matter Composition to Persistence in Aquatic Ecosystems. *Environmental Science & Technology* **2018,** *52* (5), 2538-2548.

55. McCabe, A. J.; Arnold, W. A., Reactivity of Triplet Excited States of Dissolved Natural Organic Matter in Stormflow from Mixed-Use Watersheds. *Environmental Science & Technology* **2017,** *51* (17), 9718-9728.

56. McCabe, A. J.; Arnold, W. A., Multiple linear regression models to predict the formation efficiency of triplet excited states of dissolved organic matter in temperate wetlands. *Limnology and Oceanography* **2018,** *63* (5), 1992-2014.

57. Berg, S. M.; Wammer, K. H.; Remucal, C. K., Dissolved Organic Matter Photoreactivity Is Determined by Its Optical Properties, Redox Activity, and Molecular Composition. *Environmental Science & Technology* **2023,** *57* (16), 6703-6711.

58. Cawley, K. M.; Korak, J. A.; Rosario-Ortiz, F. L., Quantum Yields for the Formation of Reactive Intermediates from Dissolved Organic Matter Samples from the Suwannee River. *Environmental Engineering Science* **2015,** *32* (1), 31-37.

59. Aiken, G.; Cotsaris, E., Soil and Hydrology - Their Effect on NOM. *J Am Water Works Ass* **1995,** *87* (1), 36-45.

60. Nguyen, H. V. M.; Hur, J.; Shin, H. S., Changes in Spectroscopic and Molecular Weight Characteristics of Dissolved Organic Matter in a River During a Storm Event. *Water Air Soil Poll* **2010,** *212* (1-4), 395-406.

61. Hodgkins, S. B.; Tfaily, M. M.; Podgorski, D. C.; McCalley, C. K.; Saleska, S. R.; Crill, P. M.; Rich, V. I.; Chanton, J. P.; Cooper, W. T., Elemental composition and optical properties reveal changes in dissolved organic matter along a permafrost thaw chronosequence in a subarctic peatland. *Geochimica et Cosmochimica Acta* **2016,** *187*, 123-140.

62. Murphy, K. R.; Stedmon, C. A.; Graeber, D.; Bro, R., Fluorescence spectroscopy and multi-way techniques. PARAFAC. *Analytical Methods* **2013,** *5* (23), 6541-6882.

63. Wologo, E.; Shakil, S.; Zolkos, S.; Textor, S.; Ewing, S.; Klassen, J.; Spencer, R. G. M.; Podgorski, D. C.; Tank, S. E.; Baker, M. A.; O'Donnell, J. A.; Wickland, K. P.; Foks, S. S. W.; Zarnetske, J. P.; Lee-Cullin, J.; Liu, F.; Yang, Y.; Kortelainen, P.; Kolehmainen, J.; Dean, J. F.; Vonk, J. E.; Holmes, R. M.; Pinay, G.; Powell, M. M.; Howe, J.; Frei, R. J.; Bratsman, S. P.; Abbott, B. W., Stream Dissolved Organic Matter in Permafrost Regions Shows Surprising Compositional Similarities but Negative Priming and Nutrient Effects. *Global Biogeochemical Cycles* **2021,** *35* (1), e2020GB006719.

64. Maizel, A. C.; Li, J.; Remucal, C. K., Relationships Between Dissolved Organic Matter Composition and Photochemistry in Lakes of Diverse Trophic Status. *Environmental Science & Technology* **2017,** *51* (17), 9624-9632.

65. Johnston, S. E.; Carey, J. C.; Kellerman, A.; Podgorski, D. C.; Gewirtzman, J.; Spencer, R. G. M., Controls on Riverine Dissolved Organic Matter Composition Across an Arctic-Boreal Latitudinal Gradient. *J Geophys Res Biogeosciences* **2021,** *126* (9), e2020JG005988.

66. Wang, K.; Pang, Y.; Gao, C.; Chen, L.; Jiang, X.; Li, P.; He, C.; Shi, Q.; He, D., Hydrological management affected dissolved organic matter chemistry and organic carbon burial in the Three Gorges Reservoir. *Water Research* **2021,** *199*, 117195.

67. Maizel, A. C.; Remucal, C. K., Molecular Composition and Photochemical Reactivity of Size-Fractionated Dissolved Organic Matter. *Environmental Science & Technology* **2017,** *51* (4), 2113-2123.

68. McCabe, A. J.; Arnold, W. A., Seasonal and spatial variabilities in the water chemistry of prairie pothole wetlands influence the photoproduction of reactive intermediates. *Chemosphere* **2016,** *155*, 640-647.

69. Hansen, A. M.; Kraus, T. E. C.; Pellerin, B. A.; Fleck, J. A.; Downing, B. D.; Bergamaschi, B. A., Optical properties of dissolved organic matter (DOM): Effects of biological and photolytic degradation. *Limnol. Oceanogr.* **2016,** *61* (3), 1015-1032.

70. Lee, E. J.; Yoo, G. Y.; Jeong, Y.; Kim, K. U.; Park, J. H.; Oh, N. H., Comparison of UV–VIS and FDOM sensors for in situ monitoring of stream DOC concentrations. *Biogeosciences* **2015,** *12* (10), 3109-3118.

71. Rode, M.; Wade, A. J.; Cohen, M. J.; Hensley, R. T.; Bowes, M. J.; Kirchner, J. W.; Arhonditsis, G. B.; Jordan, P.; Kronvang, B.; Halliday, S. J.; Skeffington, R. A.; Rozemeijer, J. C.; Aubert, A. H.; Rinke, K.; Jomaa, S., Sensors in the Stream: The High-Frequency Wave of the Present. *Environmental Science & Technology* **2016,** *50* (19), 10297-10307.

72. Ruhala, S. S.; Zarnetske, J. P., Using in-situ optical sensors to study dissolved organic carbon dynamics of streams and watersheds: A review. *Science of The Total Environment* **2017,** *575* (C), 713-723.
